# Meta-Analysis of Food Effect on Oral Absorption of Efflux Transporter Substrate Drugs: Does Delayed Gastric Emptying Influence Drug Transport Kinetics?

**DOI:** 10.3390/pharmaceutics13071035

**Published:** 2021-07-07

**Authors:** Sheena Sharma, Bhagwat Prasad

**Affiliations:** Department of Pharmaceutical Sciences, Washington State University, 412 E Spokane Falls Blvd, Spokane, WA 99202, USA; sheena.sharma@wsu.edu

**Keywords:** oral absorption, food-effect prediction, intestinal transporter, efflux transport, P-gp, BCRP

## Abstract

The oral route of drug administration is the most convenient method of drug delivery, but it is associated with variable bioavailability. Food is one of the major factors that affect oral drug absorption by influencing drug properties (e.g., solubility and dissolution rate) and physiological factors (e.g., metabolism and transport across the gastrointestinal tract). The aim of this work was to investigate the effect of food on the high-affinity intestinal efflux transporter substrate drugs. We hypothesized that transport efficiency is higher in the fed state as compared to the fasted state because of the lower intestinal lumen drug concentration due to prolonged gastric emptying time. A systematic analysis of reported clinical food-effect (FE) studies on 311 drugs was performed and the association of the efflux transport efficiency was investigated on the FE magnitude, i.e., changes in maximal plasma concentration and area under the plasma concentration–time profile curve for both solubility and permeability-limited drugs. In total, 124 and 88 drugs showed positive and negative FE, respectively, whereas 99 showed no FE. As expected, the solubility-limited drugs showed positive FE, but interestingly, drugs with a high potential for efflux transport, were associated with negative FE. Moreover, a high-fat diet was associated with a higher magnitude of negative FE for high-affinity efflux transporter substrates as compared to a low-fat diet. To account for changes in drug absorption after food intake, the prolonged gastric emptying time should be considered in the physiologically based pharmacokinetic (PBPK) modeling of orally absorbed efflux transporter substrate drugs.

## 1. Introduction

Although 85% of the top 200 prescription drugs are administered orally [1], high variability in oral drug pharmacokinetics (PK) is associated with a risk of toxicity or lack of efficacy for narrow-therapeutic index drugs [2]. Oral drug absorption is influenced by multiple extrinsic and intrinsic factors that can alter systemic drug exposure [3,4]. Food is one of the major extrinsic factors that affects the absorption of oral drugs including narrow therapeutic index drugs such as amiodarone [5], phenytoin [6], rifampicin [7], and tacrolimus [8]. Food can influence several drug properties, including dissolution rate, ionization state, complexation, and chemical stability [2]. More importantly, food intake is associated with physiological factors such as prolonged gastric emptying time (GET) [9], increased luminal viscosity [10], increased pH and luminal fluid volume [11], shorter gallbladder emptying time [12,13], increased bile acid secretion [12,14], increased splanchnic blood flow (i.e., blood draining stomach, intestine, spleen, and pancreas) [15,16], and altered drug-metabolizing enzyme and transporter (DMET) activity [17]. However, the impact of the complex interplay of altered drug properties and physiological factors on drug absorption in the fed versus the fasted state is not well characterized.

The food and drug administration (FDA) recommends food-effect (FE) bioavailability and fed bioequivalence studies prior to regulatory approval of a new or abbreviated new drug application [18,19]. Although drug safety profiles are evaluated in the fasted state during clinical trials, FE prediction is required for the first-in-human fed state trials. The current practices of FE assessment during drug development typically involve dissolution studies in the fed state simulated gastric or intestinal media [20]. In general, food can influence dissolution and/or permeability. The dissolution rate can be influenced by bile salt-mediated micellar formation that can affect the type and magnitude of FE, which is predictable by in vitro dissolution tests [21]. Five-fold higher bile acid concentration in the fed state facilitates the solubilization of solubility-limited drugs through micelle formation [14].

**Figure 1:**
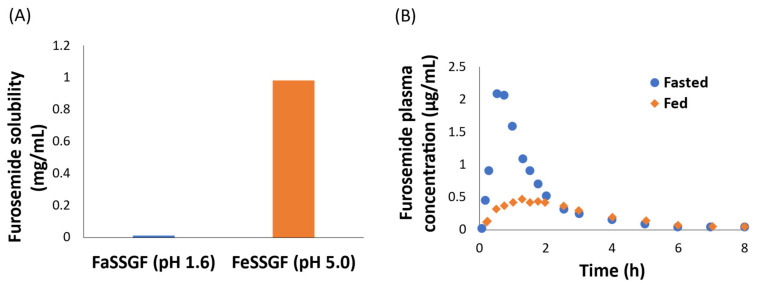
In vitro–in vivo relationship disconnect between in vitro solubility (**A**) and clinical food-effect data in furosemide (**B**). The 42% decreased AUC in fed versus fasted state is not explained by furosemide solubility data, which shows a 70-fold higher solubility in the fed state (pH 5.0) compared to fasted state (pH 1.6) in simulated gastric fluid (FeSSGF and FaSSGF, respectively).

As per the FDA, a positive or negative FE is clinically significant if the 90% confidence interval for the ratio of population geometric means of the maximum concentration (C_max_) or the area under the plasma concentration–time profile curve (AUC) in the fed state is above 125% or below 80% as compared to the fasted state. The known mechanisms of positive FE include increased bile salt-mediated solubilization [5,22], decreased first-pass metabolism due to increased splanchnic blood flow [23], and inhibition of efflux transporters [24,25] and intestinal metabolism [26,27]. In contrast, the mechanisms of negative FE include drug adsorption on food-component or bile salts [28,29], inhibition of uptake transporters [30], increased viscosity [10,31], and increased gastric pH [32]. These effects can be studied through in vitro models, utilizing dissolution studies to successfully predict the effect of pH and bile acid solubilization, and using transporter-expressing cells or vesicles to investigate the transporter inhibition [33,34]. However, physiological changes such as prolonged GET, and increased blood and bile flow cannot be simulated in dissolution studies, which is often the cause of disconnect between in vitro and in vivo data [21,35,36]. For example, the dissolution study of a breast cancer resistant protein (BCRP) substrate, furosemide, in simulated gastric fluid showed ~70-fold higher solubility in the fed versus fasted state [35], contrary to a 45% decrease in the AUC in the fed state in humans [37] (Figure 1). Considering the clinical implications of FE, it is crucial to develop a deeper understanding of the plausible mechanisms of such complex food–drug interactions.

To explain negative FE on the high-affinity efflux transporter substrates, we hypothesized that prolonged gastric emptying in the fed state results in decreased drug concentration in the intestinal lumen, which increases the transport efficiency in the fed state as compared to the fasted state. To test this, we analyzed 311 drugs with reported clinical FE studies to assess the effect of food on oral absorption of high-affinity efflux transporter substrates.

## 2. Materials and Methods

### 2.1. Collection of Physicochemical, Biochemical, and Plasma Concentration Data for Drugs with Reported Clinical Food-Effect Studies

**Figure 2:**
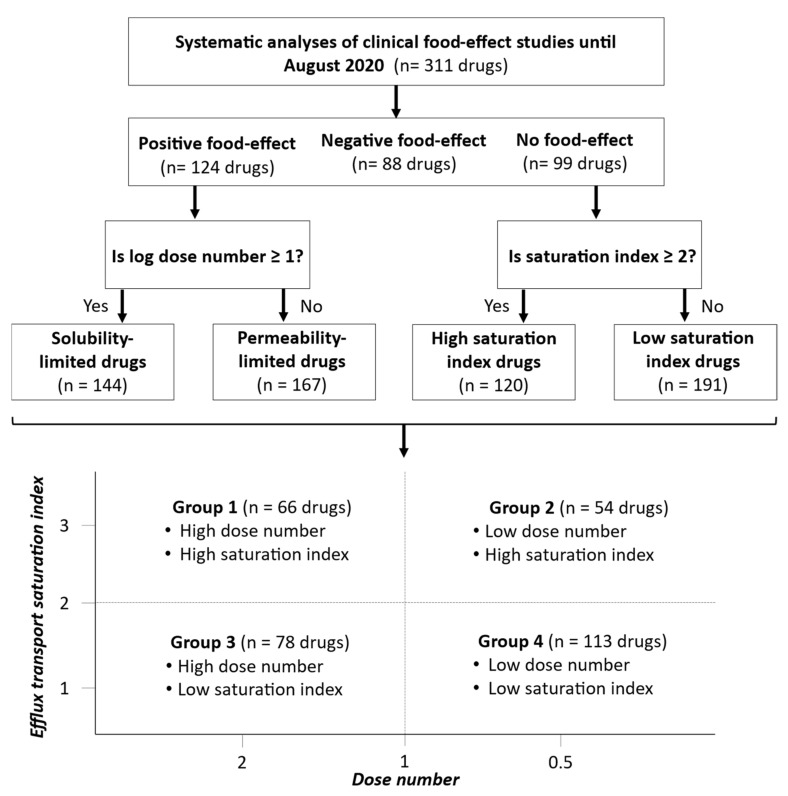
Workflow of systematic analysis of 311 drugs followed by segregation of drugs in four groups based on dose number and efflux transport saturation index. Dose number and efflux transport saturation indices were calculated based on Equations (2) and (3), respectively. Groups 1 and 3 are solubility-limited (log dose number ≥ 1), and groups 2 and 4 are permeability-limited (log dose number < 1).

A systematic literature search was conducted according to the guidelines outlined by Preferred Reporting Items for Systematic Reviews and Meta-Analysis (PRISMA) on drugs with reported clinical FE studies (n = 311 drugs) published prior to August 2020 through online search engines, i.e., PubMed, Google Scholar, and ScienceDirect (Figure 2), using the following keywords: Food and bioavailability, food and clinical pharmacokinetics, and food and drug absorption. Only human studies were included and, when more than one study was reported, the study with the greatest change in the PK parameters was considered. The C_max_, AUC, and the type of meal were compiled (Supplementary Table S1). An average American diet with a high-fat content was considered to represent the worst-case scenario.

The observed FE on drug PK parameters was calculated using Equation (1).

(1) $${\mathrm{Change}\;\mathrm{in}\;C}_{\max}\;\mathrm{or}\;\mathrm{AUC}\;\left(\%\right)=\frac{{\mathrm{Fed}}_{C_{\max}\;}\mathrm{or}\;\mathrm{AUC}-{\mathrm{Fasted}}_{C_{\max}\;}\mathrm{or}\;\mathrm{AUC}}{{\mathrm{Fasted}}_{C_{\max}}\;\mathrm{or}\;\mathrm{AUC}}\times 100$$

### 2.2. Identification of Solubility- and Permeability-Limited Drugs

Food increases the absorption of solubility-limited drugs by increasing bile micellar solubilization [5,22]. The drugs with a log dose number ≥ 1 were assumed to be solubility-limited. The dose number was calculated from drug solubility data and the respective dose strengths (Equation (2)) where the dose strength is the administered dose (mg), the volume is the total water intake (assumed 250 mL), and the solubility is in mg/mL [38].

(2) Dose number=Dose strenghtVolumeSolubility

### 2.3. Stratification of Drugs Based on Transport Saturation Index

High-affinity transporter substrate drugs (low Michaelis-Menten constant, K_m_) are prone to exhibit saturable transporter kinetics. Since the fed state delays GET from 15 min to 2 h, it is important to identify the efflux transporter substrate drugs that could likely exhibit saturable kinetics in the fasted state (shorter GET). To do so, we first determined the efficiency of efflux transport by utilizing the “saturation index” (Equation (3)), a new term, where the active transport was assumed saturable when the estimated luminal drug concentration was two-fold higher than the K_m_. The luminal drug concentration was estimated based on the administered dose strength dissolved in a typical dosing liquid volume, i.e., 250 mL [38]. If the K_m_ value for efflux transport was not reported, it was assumed 100 µM (~low-affinity) to account for the worst-case scenario.

(3) $$\mathrm{Saturation}\;\mathrm{index}=\frac{\mathrm{Luminal}\;\mathrm{drug}\;\mathrm{concentration}}{K_{m}}$$

### 2.4. Effect of Efflux Transport Saturation on Food-Effect for Permeability-Limited Drugs

The association of FE magnitude (C_max_ and AUC changes) was assessed with the log dose number and efflux transport saturation index for the studied drugs (n = 311). The drugs were stratified into four groups based on the respective thresholds of the log dose number (≥1 or <1) and the efflux transport saturation index (≥2 or <2). First, the frequency of specific FE was evaluated within each group. Then, the FE magnitude on C_max_ and AUC across four groups was analyzed using Kruskal–Wallis followed by Dunn’s multiple comparison tests using GraphPad Prism v. 8.4.3. software (San Diego, CA, USA) and RStudio version 1.2.1335 (Boston, MA, USA).

### 2.5. Impact of High- versus Low-Fat Diets on Drug Absorption

C_max_ and AUC of the drugs studied for FE in both high- and low-fat diets were compiled. The impact of diet on FE magnitude of high-affinity efflux transporter substrates was compared by the paired t-test.

## 3. Results

### 3.1. Stratification of Drugs Based on the Food-Effect Magnitude

Out of 311 studied drugs, 124 and 88 drugs were reported to have a positive and negative FE on drug absorption, respectively, whereas 99 drugs showed no FE (Supplementary Table S1). These drugs were stratified based on log dose number and efflux transport saturation indices into four groups as shown in Figure 2, i.e., (i) group 1: Log dose number ≥ 1 and efflux saturation index ≥ 2 (n = 66 drugs); (ii) group 2: Log dose number < 1 and efflux saturation index ≥ 2 (n = 54 drugs); (iii) group 3: Log dose number ≥ 1 and efflux saturation index < 2 (n = 78 drugs); and iv) group 4: Log dose number < 1 and efflux saturation index < 2 (n = 113 drugs).

#### Association of Efflux Transport Saturation on Food-Effect for Solubility- and Permeability-Limited Drugs

**Figure 3:**
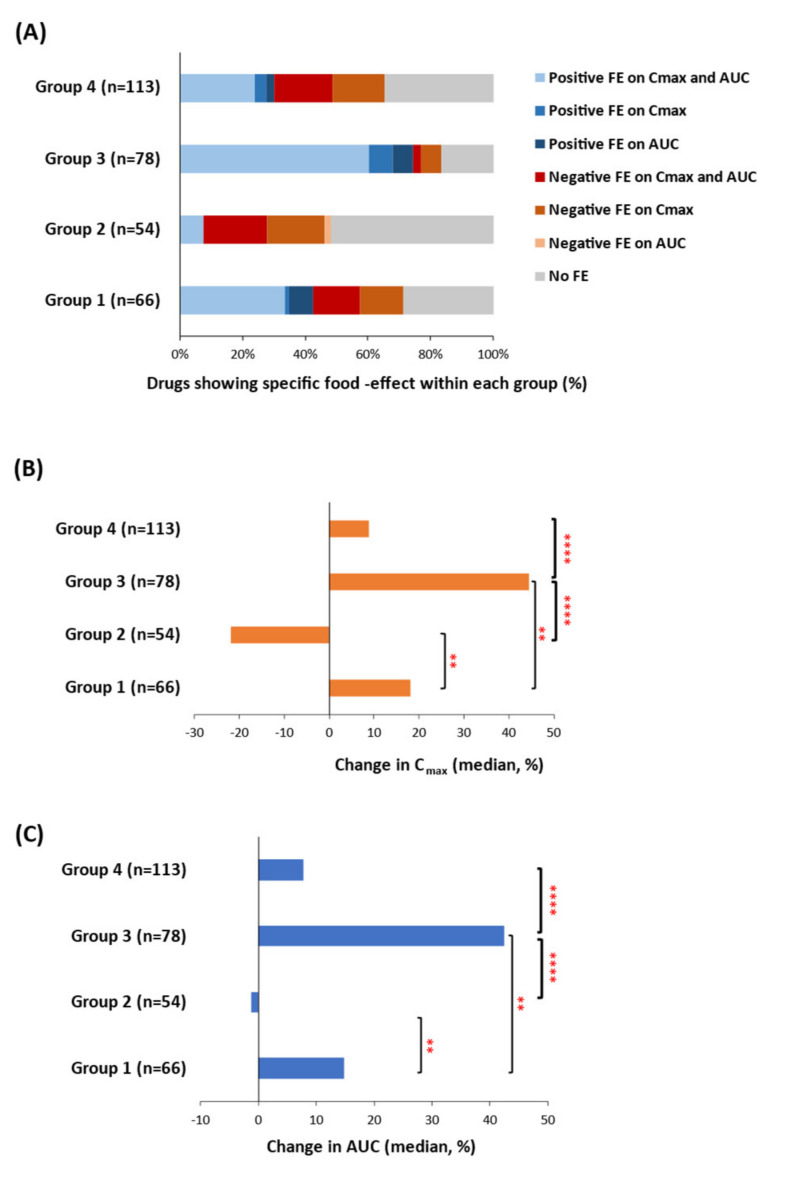
The association of solubility- and permeability-limited groups (1–4) with change in the PK endpoints (C_max_ and AUC). (**A**) Qualitative evaluation of each group to assess the frequency of drugs showing positive, negative, or no food-effect. (**B**) Changes in C_max_ (%, median) across each group. (**C**) Changes in AUC (%, median) across the four groups. Kruskal–Wallis followed by Dunn’s multiple comparison tests showed a significant difference in C_max_ (**B**) and AUC (**C**) between groups 1 vs. 2 (** *p* < 0.01), 1 vs. 3 (** *p* < 0.01), 2 vs. 3 (**** *p* < 0.0001), and 3 vs. 4 (**** *p* < 0.0001). The difference in AUC or C_max_ between all other group pairings was non-significant.

The drugs in groups 1 and 3 represent the solubility-limited drugs (log dose number ≥ 1), which, as expected, showed a higher likelihood of positive FE. The drugs in groups 2 and 4 were permeability-limited drugs (log dose number < 1), which showed a higher likelihood of having a negative or no FE (Figure 3A). In particular, significant differences (*p* < 0.05) in C_max_ and AUC (median, %) were observed between groups 1 vs. 2, 1 vs. 3, 2 vs. 3, and 3 vs. 4, as determined by Kruskal–Wallis followed by Dunn’s multiple comparison tests (Figure 3B,C and Table 1). The highest magnitude of positive FE was observed in group 3 followed by groups 1, 4 and 2, respectively, as reflected by corresponding percent changes in C_max_ (44.5 > 18 > 8.8 > −21.9), and AUC (42.4 > 14.8 > 7.8 > −1.2) (Figure 3B,C, Table 1). Consistent with our novel hypothesis, group 2 showed the highest magnitude of negative FE, calculated as C_max_ (median, −21.9%) and AUC (median, −1.2%) changes (Table 1). Asciminib, 5-fluorouracil, voriconazole, furosemide, and theophylline, are some of the high-affinity efflux transporter substrates (group 2) that show negative FE. FE is found to have a more pronounced effect on C_max_ than AUC indicating the effect of fed state on drug absorption phase.

### 3.2. Impact of High-Fat versus Low-Fat Diets on Drug Absorption

**Figure 4:**
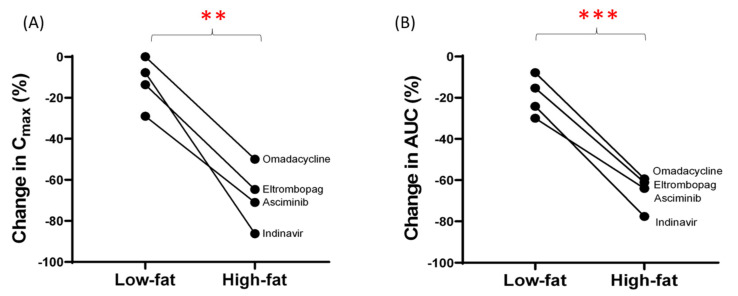
The association of diet-type on the magnitude of negative food-effect. The change in C_max_ (**A**) and AUC (**B**) were compared for the high-affinity efflux transporter substrates, i.e., omadacycline, eltrombopag, indinavir, and asciminib, with known food-effect data following a high- and low-fat diet. The longer gastric emptying time caused by the high-fat diet is a likely mechanism of the increased efficiency of efflux transport. ** and *** indicate *p* < 0.01 and *p* < 0.001, respectively (paired *t*-test).

Drugs with high-affinity efflux transport showed greater negative FE with a high-fat diet as compared to with a low-fat diet. These data also corroborate our hypothesis because a high-fat diet further delays GET. For example, omadacycline, eltrombopag, indinavir, and asciminib showed significantly decreased C_max_ (*p* < 0.01) and AUC (*p* < 0.001) after the high-fat diet as compared to the low-fat diet (Figure 4).

## 4. Discussion

The effect of food on drug absorption is one of the major causes of inter-individual variability in drug bioavailability [39,40]. Prospective prediction of FE is important for clinical trial design and dosing regimen prediction, particularly for narrow-therapeutic index drugs. In drug development, FE studies are generally conducted during the Phase II clinical trials. The potential for FE is conventionally tested based on the fed state simulated dissolution data [18,19]. However, the dissolution testing could fail to predict FE because of the complex interaction between food and gastrointestinal physiology. In particular, the interplay of efflux transporters and prolonged GET in the fed state is not well characterized.

**Figure 5:**
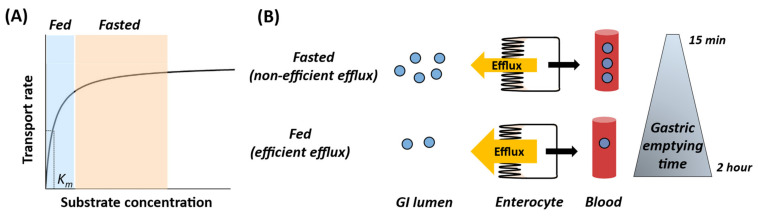
Proposed mechanisms of complex interplay of efflux transport and food-effect on drug absorption. (**A**) The phenomenon of higher efficiency of efflux transport in the fed state can be visualized by making an analogy to the classical Michaelis–Menten curve, i.e., the drug follows a linear range of transport velocity in the fed state due to the prolonged gastric emptying time (lower substrate concentration) as compared to the saturable transport in the fasted state. (**B**) The shorter gastric emptying time in the fasted state leads to a higher drug concentration relative to the Michaelis–Menten constant (K_m_). Whereas, prolonged gastric emptying time in the fed state leads to the increased efficiency of efflux transporters, resulting in a negative food-effect for efflux transporter substrate drugs. The efficiency of transport can be defined as a ratio of the rate of transport and substrate concentration, which gets increased in the fed state as compared to the fasted state.

Our data confirmed that solubility-limited drugs have a higher likelihood of positive FE, which is explained by increased solubilization due to higher bile acid secretion in the fed state as observed in the case of testosterone undecanoate [22] and amiodarone [5]. More importantly, our data analysis suggests that food associated prolonged GET affects the efficiency of apical efflux transporters in the fed state due to lower luminal drug concentrations. For example, efflux transporter substrate drugs such as asciminib [41], 5-fluorouracil [42], voriconazole [43], furosemide [37], and theophylline [44] showed negative FE. This observation can be explained by the altered transport kinetics phenomenon illustrated in Figure 5. To further support our hypothesis, we observed a higher magnitude of negative FE in the high-fat diet as compared to the low-fat diet conditions (Figure 4). Typically, a high fat diet is associated with an increased bile-micelle solubilization, which is likely a mechanism of a positive FE; however, it is contrary to our observation (Figure 4). Therefore, the negative FE in efflux transporter substrate drugs such as omadacycline, eltrombopag, indinavir and asciminib is likely due to the increased net efficiency of apical efflux transport caused by greater GET in the fed state following a high-fat meal [9]. While a high fat diet has been shown to be associated with an increased mRNA expression of Bcrp in mice [45] due to the lag time in the mRNA and protein synthesis, it is unlikely that Bcrp induction causes any clinically significant effects on the absorption phase (1–2 h) of drugs.

Our hypothesis is supported by a few reported anecdotal data. For example, consistent with our observation, Yamamoto et al. demonstrated the interplay of food and P-gp on the oral drug absorption of an investigational compound (T-3256336) in rats [46]. T-3256336 showed a three-fold lower AUC in the fed state than the fasted state (Supplementary Figure S1). However, no FE was observed when T-3256336 was co-administered with a selective P-gp inhibitor (elacridar). This suggests a P-gp dependent FE for T-3256336 that can be explained by the higher efficiency of P-gp efflux in the fed state due prolonged GET. Similarly, Sugano [47] postulated the mechanism of desaturation of apical transport and negative FE for fenoldopam in the fed state owing to lower luminal drug concentrations. These complex food and drug transport interactions are potentially more common during preclinical studies, where higher doses can likely saturate the apical transporters [48].

Recently, Xiao et al. [49] concluded that higher biliary excretion due to the increased bile flow after food is correlated with negative FE. Although the mechanism can partially explain the negative FE in drugs excreted primarily through bile, there were a few limitations of this hypothesis. First, increased bile flow typically lasts for 1–2 h, whereas biliary excretion of a drug occurs until the entire dose is eliminated from the body [12]. Since the half-life of the majority of the studied drugs is greater than 2 h, it is unlikely that the impact of greater bile flow is clinically significant on biliary excretion. Second, the threshold (>10%) used in the study for biliary clearance does not explain a clinically relevant negative FE (>25% decrease). Finally, the biliary excreted fraction was mainly calculated by fecal excretion after IV dosing, which can be confounded by intestinal elimination (basolateral uptake and apical efflux).

**Figure 6:**
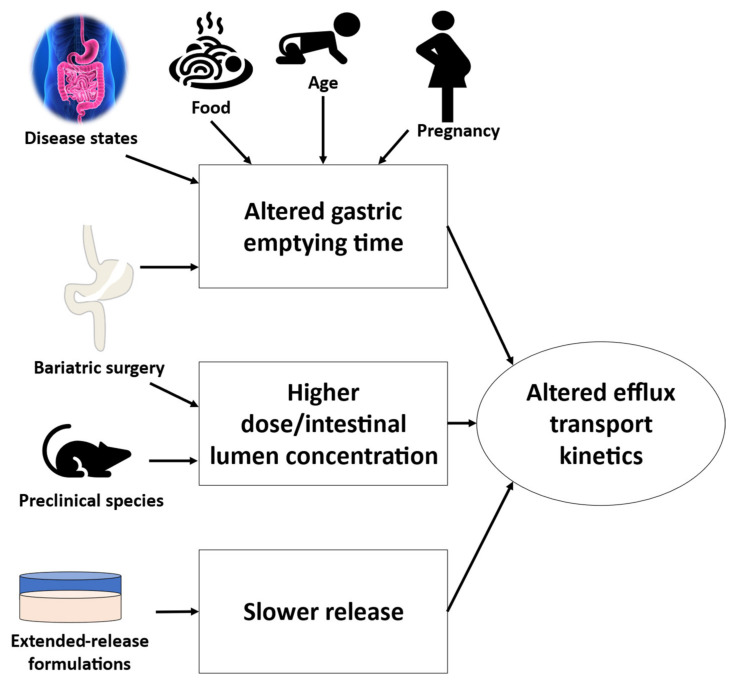
Potential applications of altered gastric emptying time-, higher dose- or intestinal lumen concentration-, and slower drug release-associated changes in efflux transport kinetics. The altered efflux transport kinetics due to the aforementioned factors can be integrated in physiologically based pharmacokinetic (PBPK) modeling of orally absorbed efflux transporter substrate drugs.

The interpretation of the impact of prolonged GET in the fed state on drug bioavailability is applicable to explain variability in the drug response in different scenarios, e.g., extended release (ER) formulations and special populations (Figure 6). For example, the saturation of transporter function in the fasted state is likely more common in the immediate release (IR) than in the ER formulation because of the slower release of the drug. Thus in the fed state, although prolonged GET leads to the higher efficiency of transporter function (Figure 5), the effect will be less significant for the ER formulation. An example that supports this hypothesis is gabapentin (L-type amino acid transporter 2, LAT2 substrate), which shows a 42% higher bioavailability in an ER formulation as compared to its IR formulation following a moderate fat diet [50].

Altered GET is also reported in children [51,52], pregnant women [53], and disease states. Drugs with GET-limiting absorption such as paracetamol, digoxin, phenobarbital, and sulfonamides, exhibit a prolonged absorption rate in children [54,55]. The apical uptake and efflux transporters are not fully mature in neonates and infants, but the net efficiency of the transport can be higher in this population due to the prolonged GET, as compared to older children (age > one year) and adults. Similarly, prolonged GET in pregnant women during 8–12 gestational weeks can affect drug absorption and transport efficiency (Figure 5). Pregnancy is associated with multifactorial changes in drug absorption and disposition including prolonged GET that can partially explain the 40% lower acetaminophen AUC in pregnancy [53]. Further, gastric surgeries and disease states such as ulcerative colitis, Crohn’s, and coeliac diseases influence gastrointestinal physiology, including the prolongation of GET, which can alter drug bioavailability [56,57,58,59,60]. Therefore, the altered GET due to food, disease, age, bariatric surgery, or pregnancy should be incorporated for reliable physiologically based pharmacokinetic (PBPK) modeling (Figure 6).

The FDA recommends clinical FE studies for investigational drugs and ER dosage forms [18,19]. The FE mechanisms such as altered gastric pH, bile secretion, and blood flow are included in the guidance. However, a systematic approach to assess the complex interplay of physiological and physicochemical factors is not provided. Understanding the effect of solubility and transport saturation on oral drug absorption can complement the guidance and provides an a *priori* outline to design clinical studies by estimating the direction of FE. The proposed mechanisms can be further tested through controlled studies.

There were a few limitations of our study as the present work is dependent on opportunistic FE data, which may be confounded by variations such as diet, co-morbidities, sample size, study population, and technical variability. Moreover, food intake primarily affects the absorption phase of a PK profile but the reported data is based on the net change in the complete AUC profile, including the elimination phase. Next, the analysis cannot test the effect of food on uptake transporter substrate drugs due to fewer drug examples being available. Lastly, the proposed threshold of log dose number and efflux transport saturation index are empirical values. These values for the studied drugs could explain the FE for the drugs under investigation but additional studies are needed to extend these thresholds to other drugs. Furthermore, the results were inconclusive about the differences in the PK endpoints of groups 1 vs. 4, and 2 vs. 4. This may be due to the wide range of FE data on C_max_ and AUC across populations. Nevertheless, our comprehensive analysis of the reported FE studies indicates that the altered interaction between GET and efflux transporters should be integrated into PBPK models to evaluate critical drug absorption parameters in the fed and fasted states.

## 5. Conclusions

This study confirmed that drugs with a higher dose number are prone to positive FE and, if these drugs are not efficiently transported by intestinal transporters, FE can be predicted by in vitro dissolution tests for these drugs. On the other hand, high-affinity efflux transport substrate drugs exhibit a higher likelihood of negative FE that is not predictable by a dissolution test. A typical PK is considered as linear first-order kinetics in the absorption, metabolism, and excretion of drugs. However, we found that the majority of transporter substrate drugs are likely saturated in the fasted state, which is an underappreciated phenomenon. We demonstrated for the first time that the efficiency of drug transporters can be increased for drugs with saturable transport kinetics in the fed state. Such effects of saturable kinetics will be more common in preclinical studies when the oral dose is much higher than the corresponding human dose. The larger magnitude of negative FE in the high-fat diet as compared to the low-fat diet supports our hypothesis. In conclusion, our proposal examines the uncharacterized effect of the interplay between active drug transporters and food on oral drug absorption. This mechanistic understanding, when validated using prospective clinical or preclinical studies, can be used to (i) design optimal FE clinical trials for investigational drugs, (ii) justify biowaivers for FE clinical studies, and (iii) explain inter-species differences in FE due to saturable kinetics.

## Figures and Tables

**Table 1:** Comparison of food-effect magnitude (change in C_max_ and AUC) across different groups.

Groups	C_max_ Change (%) *			AUC Change (%) *		
	Mean	Median	95% CI ^ⱡ^	Mean	Median	95% CI ^ⱡ^
Group 1	69.5	18	[−21.6, 48.1]	82.1	14.8	[3.9, 39.9]
Group 2	−15.4 ^ⱡⱡ^	−21.9 ^ⱡⱡ^	[−28, 10.4]	−7.4 ^ⱡⱡ^	−1.2 ^ⱡⱡ^	[−16.1, 8.2]
Group 3	619	44.5	[25.8, 84]	482.4	42.4	[25, 87]
Group 4	9.2	8.8	[−25, 19.3]	23.5	7.8	[−2, 12.5]

* C_max_ and AUC change values were estimated using Equation (1); ^ⱡ^ 95% CI, 95% confidence interval across median; ^ⱡⱡ^ Negative sign indicates negative food-effect.

## Data Availability

Data is contained within the article or Supplementary Materials.

## Supplementary Materials

The following are available online at https://www.mdpi.com/article/10.3390/pharmaceutics13071035/s1, Figure S1: P-gp-dependent food-effect due to prolonged gastric emptying time and increased efficiency of P-gp efflux in the fed state. Table S1: Drugs with reported food-effect (FE) studies and their respective physicochemical and biochemical properties.
