# Supplementary material for: Meta-Analysis of Food Effect on Oral Absorption of Efflux Transporter Substrate Drugs: Does Delayed Gastric Emptying Influence Drug Transport Kinetics?
Source: Pharmaceutics. 2021 Jul 7;13(7):1035. doi: 10.3390/pharmaceutics13071035 (PMC8309017; doi:10.3390/pharmaceutics13071035)
Supplement: Supplementary file 1 [file pharmaceutics-13-01035-s001.zip › pharmaceutics-1281739-supplementary.pdf]

pharmaceutics

Supplementary Materials: Meta−Analysis of Food Effect on Oral Absorption of Efflux Transporter Substrate Drugs: Does Delayed Gastric Emptying Influence Drug Transport Kinetics?

Sheena Sharma and Bhagwat Prasad

Table S1. Drugs with reported food−effect (FE) studies and their respective physicochemical and biochemical properties.

Drug	Food−Effect Type	Log Dose Number	Transport Saturation Index	Mechanism of Elimination	C_{max} Change (%)	AUC Change (%)	References
5−Aminosalicylic acid	Negative	−0.48	NS	Acetylation (metabolism)	−64.47	−47.75	[1,2]
5−fluorouracil	Negative	−0.86	61.50	BCRP (GI efflux), OAT2	−70.43	−34.92	[3]
6−Mercaptopurine	Negative	−0.09	NS	Aldehyde oxidase (AO), Methyltransferase, xanthine oxidase (XO), Xanthine dehydrogenase (XDH) (metabolism), MRP4, ENBT1	−25	−25	[4]
Abacavir	Negative on C_{max}	0.00	41.91	Alcohol dehydrogenase, UGTs (metabolism), P−gp, BCRP (GI efflux)	−26	−3.1	[5,6]
Abiraterone acetate	Positive	2.82	51.08	Esterase, CYP3A4, SULT2A1 (metabolism), P−gp (GI efflux)	630.88	343.29	[7]
ABT−102	None	−0.10	NS	NA	6.98	20.22	[8,9]
ABX−464	Positive	2.10	NS	UGTs (metabolism)	183.67	161.91	[10]
Acetaminophen	None	−0.02	NS	UGT1A1/1A9/2B15, SULT1A1/1A3/1E1/2A1, CYP1A2/2C9/2C19/2D6/2E1/3A4 (metabolism)	21	NA	[11,12]
Acipimox	None	−1.08	NS	Minimal metabolism	NA	NA	[13,14]
Adinazolam	Positive	−0.05	NS	Hepatic metabolism	32.6	9.24	[15]

Afatinib	Negative	0.80	1.65	P−gp/BCRP (GI efflux)	−50	−39	[16–18]
Albendazole	Positive	1.09	10.55	CYP3A4 (metabolism), P−gp (GI efflux)	550	840	[19–21]
Alectinib	Positive	2.06	24.86	CYP3A4, aldehyde dehydrogenases (ADH) (metabolism), P−gp (GI efflux)	164.12	188.42	[22,23]
Alitretinoin	Positive	1.53	NS	CYP3A4 (metabolism)	225.98	295.33	[24]
Alprazolam	None	−0.91	NS	CYP3A4 (extensive metabolism)	23	NA	[25,26]
Amifampridine phosphate	Negative on C_{max}	−3.30	NS	N−acetyl transferases (NAT2) (metabolism) (variability due to fast and slow acetylators)	−31.3	−8.85	[27–29]
Amiodarone	Positive	2.70	NS	CYP3A4/2C8 (metabolism)	268.42	135.71	[30,31]
Amitriptyline	None	1.35	NS	UGTs, SULTs (metabolism)	NA	NA	[32]
Amlodipine	None	0.43	0.49	CYP3A4 (metabolism), P−gp (GI efflux)	NA	NA	[33,34]
Amocarzine	Positive	1.84	NS	Extensive hepatic metabolism	170.21	204.55	[35,36]
Amoxycillin	None	0.32	NS	CYPs (metabolism)	NA	5.2	[37]
Ampicillin	Negative	0.52	57.24	MRP2 (GI efflux), PEPT1 (GI uptake), hydrolysis (metabolism)	−25.92	−31.03	[37,38]
Apixaban	None	−0.23	0.87	P−gp (GI efflux) and CYP3A4 (metabolism)	15	21	[39,40]
Aprepitant	Positive	1.79	NS	CYP3A4/1A2/2C19 (extensive metabolism)	314.33	303.45	[41,42]
Artemisinin	None	0.20	NS	CYPs (metabolism)	NA	NA	[43]
Asciminib	Negative	0.88	13.34	UGT2B7, CYP3A4 (metabolism), P−gp/BCRP (GI efflux)	−71.23	−63.55	[44,45]
Atazanavir	Positive	2.69	22.70	CYP3A (extensive metabolism),	57	70	[46], FDA label

				P−gp, MRP1 (GI efflux)			
Atenolol	None	−0.03	15.02	Minimal metabolism, P−gp (GI efflux)	NA	20	[47]
Atorvastatin	Negative	2.71	5.73	P−gp (GI efflux) and CYP3A4 (metabolism)	−47.83	−12.67	[48,49]
Atovaquone	Positive	3.58	81.78	CYP (metabolism), P−gp (GI efflux)	400	200	FDA label
Bendroflumethiazide	None	−0.73	NS	Extensive metabolism	NA	NA	[50,51]
Bevantolol	None	1.47	NS	Extensive metabolism	12.6	7.47	[52]
Bidisomide	Negative	0.13	NS	NA	−52.97	−49.04	[53]
Blonanserin	Positive	−0.65	0.22	P−gp (GI efflux), CYP3A4 (metabolism)	242.32	283.63	[54,55]
BMS−690514	Positive	0.42	NS	CYPs, UGTs (metabolism)	54.96	33	[56,57]
Boceprevir	Positive on AUC	2.14	61.58	Aldoketoreductase−mediated metabolic pathway, CYP3A4/5 (metabolism), P−gp (GI efflux)	NA	60	[58]
Bosentan	None	1.74	9.06	CYP2C9/3A4 (metabolism), OATP2B1 (GI uptake)	22	10	[59,60]
Bosutinib	Positive	2.23	30.16	P−gp (GI efflux), CYP3A4 (metabolism) [B:P =1.2]	80	70	[61], FDA label
Bromazepam	Negative	0.01	NS	CYPs (metabolism)	−34.74	−33.13	2273442, 30115648
Bromfenac	Negative on C_{max}	0.90	2.99	OATP1B1, BCRP (GI efflux)	−72	NA	[62,63]
Bromocriptine	None	−0.46	NS	CYP3A/2D6/2C8/2C19 (extensive metabolism)	17.65	8.77	[64,65]
Bumetanide	Negative on C_{max}	−0.51	0.22	UGTs (metabolism), OAT1, P−gp (GI efflux)	−62.89	−11.89	[66,67]
Buspirone	Positive	−0.87	NS	CYP3A4 (extensive metabolism)	116	84	FDA label
Cabozantinib	Positive	2.55	13.96	CYP3A4 (extensive metabolism),	48.13	66.14	[68,69]

				MRP2 (GI efflux)			
Canagliflozin	None	2.43	NS	UGT1A9 and UGT2B4 (metabolism)	NA	7.88	[68,70]
Candesartan	Positive on C_{max}	0.63	0.73	Intestinal ester hydrolysis, hepatic O−deethylation (metabolism), P−gp (GI efflux)	26.6	NA	[71]
Canertinib (CI−1033)	None	1.81	20.58	OATPs, BCRP (GI efflux)	NA	NA	[72]
Cannabidiol	Positive	1.50	NS	CYP2C19/3A4 enzymes, and UGT1A7, UGT1A9, and UGT2B7 (metabolism)	1400	384.9	[73]
Captopril	Negative	−1.05	18.41	Methyltransferase (metabolism), PEPT1 (GI uptake)	−48.7	−38.95	[74]
Carboxyamidotriazole	Positive	2.75	NS	CYP3A4 (metabolism)	212	166.09	[75]
Cediranib	Negative on C_{max}	1.06	4.00	[BA and UGT1A4 (major), FMO1, FMO3, CYPs (minor) (metabolism), P−gp (GI efflux)	−31.74	−19.73	[76,77]
Cefpodoxime proxetil	None	0.64	NS	Esterase (metabolism)	NA	NA	[78]
Cefuroxime axetil	Positive	0.85	39.18	Esterases (metabolism), PEPT1 (GI uptake)	42.85	44.97	[79,80]
Celecoxib	Positive	1.62	5.51	CYP2C9 (metabolism). P−gp (GI efflux)	29.28	11.49	[81]
Ceritinib	Positive	2.95	35.83	CYP3A4 (metabolism), P−gp (GI efflux)	47.79	83.79	[82,83]
Chloroquine	Positive	2.14	46.52	CYP3A4 (metabolism), P−gp (GI efflux)	51.5	41.81	[84,85]
Chlorothiazide	Positive	0.70	67.63	BCRP (GI efflux), limited metabolism	25	25	[86,87]
Cibenzoline	None	2.12	NS	CYP3A/2D6 (metabolism)	16	NA	[88]
Cilazapril	Negative	−1.72	0.48	Hydrolysis (metabolism), PEPT1 (GI	−28.9	−13.82	[89,90]

				uptake), P−gp (GI efflux)			
Cilostazol	Positive	1.09	10.83	CYP3A4/2C19 (metabolism), OATP1B1−, and OATP1B3, MRP2, BCRP, P−gp (GI efflux)	94.56	25.51	[91,92]
Cinnarizine	Positive	1.76	NS	Extensive hepatic metabolism	28.26	57.14	[93]
Ciprofloxacin	Negative on C_{max}	0.17	60.36	CYP1A2 (metabolism), BCRP (GI efflux)	−33	NA	[94,95]
Ciramadol	None	−1.17	NS	UGTs (metabolism)	18.8	3.81	[96]
Clodronate	Negative	−0.81	NS	No metabolism	−77.8	−90.2	[97,98]
Clopidogrel	Positive	1.41	9.32	Esterase, CYPs (metabolism), P−gp (GI efflux)	515.6	869.67	[99,100]
Clozapine	None	−0.57	1.53	CYP1A2, 2C, 2D6, 2E1 and 3A3/4 (extensive metabolism), OATPs, P−gp (GI efflux)	20	8.05	[101,102]
Cobicistat	None	2.68	7.73	CYP3A, 2D6 (metabolism), OCT2, P−gp (GI efflux)	NA	6.88	[103]
Cobimetinib	None	0.28	1.51	CYP3A, UGT2B7 (metabolism), P−gp (GI efflux)	7.5	10	[104,105]
Codeine	None	−0.38	NS	UGT2B7/2B4, CYP3A4/2D6 (metabolism), OCT1	NA	NA	[106,107]
Crizotinib	None	2.21	22.21	P−gp (GI efflux) and CYP3A4 (metabolism)	14.07	14.27	[108], FDA label
Cyclandelate	Positive	0.60	NS	Metabolism	25	25	[109]
Cyclosporine	Positive	1.45	NS	CYPs (metabolism)	25	25	[110]
Dabrafenib	Negative	2.26	11.55	CYP2C8, CYP3A4 (metabolism), P−gp, BCRP (GI efflux)	−50.64	−30.08	[111,112]
Danazol	Positive	1.36	NS	CYP3A4 (metabolism)	140	290.83	[113,114]
Darunavir	Positive	1.68	NS	CYP3A4 (metabolism)	62.11	38.57	[115]

Deferasirox	Positive	2.21	NS	UGT1A1/1A3, CYPs (metabolism)	43.55	31.05	[116,117]
Deflazacort	None	0.92	3.26	Esterase (metabolism), P−gp (GI efflux)	17.02	0.78	[118,119], FDA label
Deramciclane	Positive on AUC	2.07	NS	CYP2E1 (Extensive metabolism)	24	31	[120]
Desloratadine	None	0.88	0.97	UGT2B10, CYP2 C8 (metabolism), P−gp (GI efflux)	NA	NA	[121,122]
Desmopressin	Negative	−2.14	NS	Intestinal degradation	−45.91	−40.54	[123,124]
Dextropropoxy phene	None	1.79	NS	CYP3A4 (metabolism)	NA	NA	[107,125]
Diclofenac	Negative on C_{max}	1.65	6.75	CYP2C9 (metabolism), OATP1B3, P−gp/BCRP (GI efflux)	−61	−15	[126]
Dicoumarol	Positive on AUC	1.18	NS	CYP2C (metabolism)	NA	242	[127]
Didanosine	Negative	−0.64	63.50	BCRP (GI efflux), Purine nucleoside phosphorylase (PNP)	−53.71	−46.61	[128,129]
Digoxin	Negative	−1.60	0.04	P−gp (GI efflux)	−25	−25	[130]
Diprafenone	Positive on AUC	0.82	NS	CYP2D6 (metabolism)	8.78	36.16	[131,132]
Dixyrazine	Positive	0.49	NS	Degradation	25	25	[133,134]
Dolutegravir	Positive	0.34	4.77	UGT1A1, UGT1A3, UGT1A9 (metabolism), BCRP, and P−gp (GI efflux)	36.89	48.27	[135,136]
Doravirine	None	2.10	NS	CYP3A4/5 (metabolism)	15.53	17.94	[137,138]
Dovitinib	None	1.16	NS	CYP1A1/2, flavin−containin g monooxygenase (FMO) (metabolism)	5.89	8.8	[139,140]
Doxycycline	None	0.10	18.00	CYPs (metabolism), P−gp (GI efflux)	20	NA	[141–143]
Dronedarone	Positive	2.90	28.74	CYP3A4 (metabolism), P−gp (GI efflux)	25	25	[144]
Duloxetine	Positive on C_{max}	1.91	NS	CYPs 1A2, 2D6 (extensive metabolism)	31.33	4.24	[145,146]

Efavirenz	None	2.45	76.03	CYP2B6 (metabolism), BCRP (GI efflux)	16.81	13.58	[147,148]
Eltrombopag	Negative	1.30	4.52	UGT1A1, UGT1A3, CYP1A2, CYP2C8 (metabolism), BCRP (GI efflux), OATP1B1	−64.71	−59.36	[149,150]
Elvitegravir	Positive	1.96	NS	CYP3A4, UGT1A1/3 (metabolism)	56	87	[58,151]
Enoxacin	None	0.17	NS	CYPs (metabolism), MATE−1	9.6	8.6	[151,152]
Entecavir	Negative on C_{max}	−3.22	0.14	BCRP (GI efflux)	−62.97	−22.36	[153,154]
Eprosartan	Negative on C_{max}	2.14	23.05	OATP1B1 and MRP2 (GI efflux)	−25.25	−14	[155]
Erlotinib	Positive	1.83	15.25	BCRP (GI efflux)	57.16	100.25	[156,157]
Erythromycin	Negative	0.64	27.25	CYP3A4 (metabolism), P−gp (GI efflux)	−53.78	−56.45	[158,159]
Eslicarbazepine	None	0.76	107.99	Hydrolysis (metabolism), P−gp (GI efflux)	NA	NA	[160–162]
Estramustine	Negative	3.65	38.15	CYPs (metabolism), P−gp (GI efflux)	−72.68	−71.07	[163]
Ethionamide	None	0.38	NS	EthA enzyme (metabolism)	NA	NA	[164,165]
Etoposide	None	−0.39	6.80	CYP3A4/5 (metabolism), P−gp (GI efflux)	22	12.25	[166,167]
Etoricoxib	Negative on C_{max}	2.17	NS	CYPs (metabolism), MRP4	−35.8	−2.88	[168,169]
Evacetrapib	Positive	2.21	NS	CYP3A/CYP2C8 (metabolism)	51.05	44.29	[170,171]
Everolimus	Negative on C_{max}	0.69	0.08	P−gp (GI efflux) and CYP3A4 (metabolism)	−60.34	−20.49	[172,173]
Fedratinib	Positive on AUC	2.32	38.12	CYP3A4, CYP2C19, and flavin−containing monooxygenase 3 (FMO3) (metabolism), P−gp (GI efflux)	3.57	30.73	[174], FDA label

Fenofibrate	Positive	3.15	NS	Esterase, UGTs, CYPs (metabolism)	282.84	234	[175,176]
Fenoldopam	Negative	0.17	NS	Glucuronidation, sulfation, and methylation (metabolism)	−79.94	−63.25	[177,178]
Fexinidazole	Positive	1.71	NS	CYP1A2, 2B6, 2C19, 3A4, and 3A5 and, to a lesser extent, 2D6 (metabolism)	291.6	247	[179]
Fiduxosin	Positive	1.78	NS	NA	337.32	136.01	[180,181]
Flecainide	None	1.39	19.31	CYP3A4/2D6 (metabolism), P−gp (GI efflux)	4.11	13.08	[182]
Flubendazole	Positive	1.78	NS	Metabolism	25	25	[183]
Fluconazole	None	−0.54	13.06	CYP3A4 (metabolism), P−gp (GI efflux)	5.12	6.19	[184]
Fluvoxamine	None	1.44	4.60	CYPs (metabolism), P−gp (GI efflux)	NA	−7	[185,186]
Fosamprenavir (prodrug)	Negative	0.61	47.81	CYP3A4 (metabolism), P−gp (GI efflux)	−28	−46	[187]
Fosaprepitant	Positive	2.02	NS	CYP3A4 with minor metabolism by CYP1A2 and CYP2C19 (metabolism)	33	47	[188], EMEA label
Fostamatinib (R406 prodrug)	None	0.59	3.45	CYP3A4, UGT1A9 (metabolism), P−gp (GI efflux)	NA	NA	[189,190]
Furosemide	Negative	0.12	4.84	P−gp/BCRP (GI efflux), OATP2B1 (GI uptake)	−44	−33	[66]
Gabapentin	Positive	−1.62	140.16	LAT1/2 (GI uptake)	32	26	[191–193]
Ganciclovir	None	−0.46	156.72	Minimal metabolism, P−gp (GI efflux)	12.94	19.14	[194]
Gatifloxacin	None	0.40	42.62	P−gp (GI efflux)	8.57	7.01	[195]
Gefitinib	None	1.57	22.38	CYP3A4/3A5/2D6 (metabolism), P−gp, BCRP (GI efflux)	NA	NA	[196], monograph
Gemifloxacin	None	0.78	32.87	P−gp, MRP2 (GI efflux)	14	12	[197,198]
Gepirone	Positive on AUC	−1.13	NS	CYP3A4 and CYP2D6 (metabolism)	−9.34	36.97	[199]

Ginkgolide A	Positive	−0.48	NS	CYPs (metabolism)	118.11	85.64	[200,201]
Glasdegib	Negative on C_{max}	0.93	10.68	P−gp (GI efflux) and CYP3A4 (metabolism)	−25.68	−10.96	[202,203]
Glyburide	None	0.99	0.40	CYP3A4/2C9/2C19/3A7/3A5 (metabolism), P−gp, BCRP (GI efflux), MRP1	11.03	12.34	[204]
Griseofulvin	Positive	1.60	NS	Desmethylation, UGTs (metabolism)	25	25	[205,206]
GW420867X	None	0.52	6.71	CYPs (metabolism), P−gp (GI efflux)	NA	NA	[207]
Halofantrine	Positive	3.95	19.98	CYP3A4 (metabolism), P−gp (GI efflux)	561.95	189.74	[208,209]
Hydralazine	Positive	−1.12	NS	Extensive−first pass metabolism by acetylation	143.67	254	[210,211]
Hydrocodone	Positive on C_{max}	−0.35	NS	CYPS 3A4, 2D6 (metabolism)	42.07	11.18	[212–214]
Ibrutinib	Positive	1.92	38.14	CYP3A, and to a minor extent by CYP2D6 (extensive metabolism), P−gp (GI efflux)	281.82	158.9	[215,216], FDA label
Icotinib	Positive	2.10	NS	CYP3A4, CYP3A5, and CYP1A2 (metabolism)	59	79	[217,218]
Imipramine	None	0.48	7.13	CYP2C19/1A2/3A4 (extensive metabolism), P−gp (GI efflux)	NA	NA	[219,220]
Imiquimod	None	0.21	NS	NA	NA	NA	[221,222]
Indinavir	Negative	1.51	26.07	CYP3A4 (metabolism), P−gp (GI efflux)	−86	−78	[223,224]
Indomethacin	Negative on C_{max}	1.62	2.79	CYP2C9, 2C19, 2D6 (metabolism), P−gp (GI efflux)	−29	1.5	[225,226]
Ipriflavone	Positive	1.66	NS	Extensive metabolism	25	25	[227]
Isoniazid	Negative on C_{max}	−1.46	NS	NAT2, CYP2E1 (metabolism)	−56.04	−11.94	[228]
Isosorbide−5−mononitrate	None	−2.55	NS	Hepatic metabolism	−20	16.45	[229,230]
Isotretinoin	Positive	1.83	NS	2C8, 2C9, 3A4, and 2B6 (metabolism)	144.44	86.03	[231]

Itraconazole	Positive	1.62	NS	CYP3A4 (metabolism)	109	69.75	[232,233]
Ivermectin	Positive	1.78	NS	Extensive metabolism	207.19	164.72	[234,235]
Ixazomib	Negative	−0.05	0.44	CYPs (metabolism), P−gp (GI efflux)	−70.39	−32.04	[236–238]
Ketoconazole	Negative	1.90	15.05	CYP3A4 (metabolism), P−gp (GI efflux)	−43.21	−39.15	[239]
Labetalol	None	2.14	21.92	UGTs (metabolism), P−gp (GI efflux)	−21.64	19.29	[240]
Lacosamide	None	0.24	31.96	CYP2C9, CY2C19, and CYP3A4 (metabolism), P−gp (GI efflux)	NA	NA	[241]
Lanopepden (GSK1322322)	Positive on AUC	1.12	NS	UGTs (metabolism)	NA	27.22	[242]
Lapatinib	Positive	2.43	103.26	CYP3A4 and CYP3A5 (extensive metabolism), P−gp, BCRP (GI efflux)	203	325	[243,244]
Lenalidomide	Negative on C_{max}	−1.07	7.71	P−gp (GI efflux), minimal metabolism	−50	−20	[245]
Lenvatinib	None	0.81	0.94	CYP3A and aldehyde oxidase (metabolism), P−gp, BCRP (GI efflux)	−5	6	[246], FDA label
Lesinurad	None	2.31	39.57	CYP2C9 (metabolism), BCRP (GI efflux)	16.19	10.35	[247,248]
Lesogaberan	None	−2.52	NS	Hepatic metabolism	24	NA	[249,250]
Levetiracetam	None	−1.87	235.01	Enzymatic hydrolysis and hydroxylation (metabolism), P−gp (GI efflux)	NA	NA	[251]
Levofloxacin	None	−0.08	33.21	Limited metabolism, OATP1A2 (GI uptake), BCRP (GI efflux)	13.55	9.7	[252–254]
Linezolid	None	0.02	44.46	P−gp (GI efflux) and CYP3A4 (metabolism)	18.42	3.29	[255,256]

Linifanib	Negative on C_{max}	−0.11	2.05	CYP3A4 (metabolism), P−gp (GI efflux)	−37.11	−15.16	[255,257]
Lisinopril	None	−0.43	1.97	Limited metabolism, P−gp (GI efflux)	NA	NA	[258,259]
Lonafarnib	Negative on C_{max}	2.68	NS	CYP3A4, CYP3A5, CYP2C8 (extensive metabolism)	−52	−23	[260,261]
Lopinavir	Positive on AUC	2.92	25.44	P−gp, MRP2 (GI efflux), CYP3A4 (metabolism)	18	27	[262,263]
Loratadine	Positive	0.47	1.04	CYP3A4 (metabolism), P−gp (GI efflux)	52.87	75.77	[264,265]
Losartan	None	1.93	9.46	CYPs (metabolism), P−gp (GI efflux)	−16.16	14.83	[266]
Lumefantrine	Positive on C_{max}	4.19	9.07	CYP3A4 (extensive metabolism), P−gp (GI efflux)	100	NA	[267]
Lurasidone	Positive	1.01	NS	CYP3A4 (extensive metabolism)	97	103.25	[268,269]
Manidipine	Positive	1.91	NS	CYP3A4 (extensive metabolism)	25.81	42.4	[270,271]
Maraviroc	Negative	1.05	2.34	P−gp (GI efflux) and CYP3A4 (metabolism)	−33	−33	[272], FDA label
Mavoglurant	Positive	1.21	NS	CYP2C8, CYP2C9, CYP2C19 with minor to negligible contributions of CYP2D6, CYP3A4, and CYP1A1 (extensive metabolism)	25	25	[273]
Mebendazole	Positive	2.19	NS	Extensive metabolism	25	25	[274,275]
Mefloquine	Positive	1.90	79.30	CYP3A4 (metabolism), P−gp (GI efflux)	72.8	39.91	[276]
Megestrol	Positive	2.28	NS	CYP3A4, UGT2B17 (metabolism)	629.41	139	[277,278]
Melphalan	Negative	−0.95	NS	Hydrolysis to mono and dihydroxy	−42.2	−31.8	[279]

				products (metabolism)			
Menatetrenone (Vit K2)	Positive	2.09	NS	NA	25	25	[280]
Metformin	None	0.16	NS	OCTs, MATEs (mainly renal)	15.55	3.79	[281,282]
Methotrexate	None	−0.76	0.66	BCRP (GI efflux)	19.31	NA	[283]
Methylphenidate	None	−0.06	6.86	CES1A1 (metabolism), P−gp (GI efflux) (variability due to d and l enantiomers)	22.75	15.42	[284,285]
Metoclopramide	None	−0.41	4.00	CYP2D6/3A/1A2 (metabolism), P−gp and BCRP (GI efflux)	NA	NA	[286,287]
Metoprolol	Positive	0.00	NS	CYP2D6 (extensive metabolism)	123.53	132	[288,289]
Migalastat	Negative	−3.11	NS	O−glucuronidation (metabolism), SGLT−1 (minor)	−40.93	−37.93	[290,291]
Mirabegron	Negative	1.69	58.72	P−gp (GI efflux), OATP1A2 (GI uptake) and CYP (metabolism)	−72.84	−47.73	[292,293]
Mirtazapine	None	−1.26	NS	CYP1A2, CYP2D6, CYP3A4 (metabolism)	5.26	12.5	[294,295]
MMI270B	Negative on C_{max}	0.05	NS	Metabolism	−55.5	−17.34	[296]
Momelotinib	None	1.39	19.30	CYPs (metabolism), Pgp, BCRP (GI efflux)	24.17	22.44	[297,298]
Nadolol	Negative on C_{max}	−0.85	10.34	Renal excretion mainly, P−gp (GI efflux)	−34.4	−23.76	[299,300]
Nalidixic acid	Positive	−0.36	NS	CYPs, UGTs (metabolism)	91.3	87.78	[301,302]
Naloxegol	Positive	0.25	1.53	P−gp (GI efflux), CYP3A4 (metabolism)	30	45	[303], EMEA label
Nateglinide	Negative on C_{max}	1.45	NS	CYP2C9, CYP3A (metabolism), OATs, Monocarboxylate transporter (MCT) 6	−33.66	−5.63	[304,305]

Nelfinavir	Positive	3.42	NS	CYP2C19 and CYP3A (metabolism)	420	230	[306], FDA label
Nifedipine	None	1.13	6.93	CYP3A4 (metabolism), BCRP (GI efflux)	22.85	9.32	[307,308]
Niflumic acid (given as talniflumate−Prodrug)	Positive	1.53	NS	UGTs (metabolism)	417.41	386.38	[309]
Nilotinib	Positive	2.90	30.22	CYP3A4 (metabolism), P−gp/BCRP (GI efflux)	112	82	[310]
Nilvadipine	None	0.29	NS	Extensive first pass metabolism	NA	NA	[311,312]
Nitrofurantoin	Positive	−0.02	16.80	Extensive metabolism, BCRP (GI efflux)	25	25	[313]
Norfloxacin	None	0.20	50.10	CYP1A2 (limited metabolism), BCRP (GI efflux)	NA	NA	[314,315]
Omadacycline	Negative	0.75	20.20	P−gp (GI efflux)	−50	−61.11	[316,317]
Orteronel	Negative	1.17	52.07	P−gp, BCRP (GI efflux), CYP (minor) (metabolism)	−50.62	−40.18	[318], FDA monograph
Ospemifene	Positive	2.66	NS	CYP3A4, CYP2C9, CYP2C19, and CYP2B6 (metabolism)	260	180	[319]
Oxaprozin	None	2.17	NS	CYPs, UGTs (metabolism)	NA	NA	[320,321]
Oxfendazole	Positive	0.56	NS	Extensive metabolism	238.46	673.86	[322–324]
Oxybutynin	Positive on C_{max}	0.60	1.12	CYP3A4 (extensive metabolism), P−gp (GI efflux)	121.43	6.06	[325–327]
Palbociclib	Positive on C_{max}	1.46	NS	CYP3A, SULT2A1 (metabolism)	36.84	18.75	[328,329]
Panobinostat	Negative on C_{max}	1.98	2.29	P−gp (GI efflux) and CYP3A4 (metabolism)	−47.82	−18.18	[330,331], FDA label
Penicillin	Negative	0.55	29.90	PEPT1 (GI uptake)	−25	−25	[332]
Pexmetinib	Positive on C_{max}	4.51	NS	NA	49.04	20.51	[333]

PF−04449913 (Glasdegib maleate B)	None	0.93	10.68	CYP3A4/5 (metabolism), P−gp, BCRP (GI efflux)	16.56	18.66	[334]
Phenytoin	Positive	1.23	NS	CYPs (metabolism)	45.45	109	[335,336]
Pictilisib (GDC−0941)	None	0.38	3.12	P−gp and BCRP (GI efflux)	−23.08	17.6	[337]
Piperaquine	Positive	2.49	NS	CYP3A4 (metabolism)	217	172.66	[338]
Pitavastatin	Negative on C_{max}	0.61	0.38	UGT1A3, UGT2B7, CYP2C9, CYP2C8 (metabolism), P−gp/MRP2/BCRP (GI efflux), OATP1B1/1B3/2B1/NTCP	−43.1	−11	[339], FDA label
Pleconaril	Positive	1.56	NS	Extensive metabolism	147.83	122.55	[340,341]
Ponatinib	None	1.79	3.38	CYP3A (metabolism), P−gp, BCRP (GI efflux)	NA	NA	[342,343], FDA label
Posaconazole	Positive	1.82	11.42	UGTs (metabolism), P−gp (GI efflux)	287.88	290.8	[344–346]
Pradigastat	Positive	2.20	NS	NA	36.02	30.87	[347,348]
Prazosin	None	−2.24	0.10	CYPs (metabolism), BCRP (GI efflux)	NA	NA	[107]
Pregabalin	Negative on C_{max}	−1.73	37.68	LAT1/2 (GI uptake)	−25	NA	[192]
Preladenant	Negative on C_{max}	−0.34	NS	O−desmethylation, carboxylation (metabolism)	−38.16	−4.47	[349,350]
Pretomanid (PA 824)	Positive	1.23	NS	CYP3A4 (metabolism)	38.01	43.23	[351]
Primaquine	Positive on C_{max}	0.33	NS	CYP2D6, UGTs (Extensive metabolism)	26	14	[352–354]
Procainamide	None	0.00	NS	OCT3, CYP2D6 (metabolism)	NA	NA	[355]
Propafenone	Positive on C_{max}	2.20	NS	CYP2D6 (Extensive metabolism)	50.31	7.37	[356,357]
Propranolol	Positive	0.61	NS	CYP2D6, 1A2, and 2C19 (metabolism)	87.5	127.13	[288]
Pseudoephedrine	None	−0.93	NS	Extensive hepatic metabolism	7.31	−1.57	[358,359]

R1663	None	1.90	NS	CYP3A4 (extensive metabolism)	NA	NA	[360]
Raltegravir	Positive	0.31	NS	UGTs (metabolism), OAT1	96.31	112	[361,362]
Ravuconazole	Positive	−0.60	NS	NA	25	25	[363]
Repirinast	Positive on C_{max}	2.48	NS	NA	220	NA	[364]
Ribavirine	Positive	−1.62	32.76	CNT2 and ENT1 (GI uptake),	66	42	[365], FDA label
Riboflavin (Vitamin B2)	Positive	−2.61	0.04	hRFT−1, hRFT−2 and hRFT−3 (GI uptake)	25	25	[366,367]
Rifabutin	None	1.55	NS	Hepatic metabolism	16.87	NA	[368]
Rifalazil	Positive	0.94	NS	Esterase, CYP3A4 (metabolism)	101.22	44.66	[369]
Rifampicin	Negative on C_{max}	1.76	NS	Desacetylation (metabolism), Degradation, ET (P−gp)	−35.77	−6.4	[370,371]
Rilpivirine	Positive	1.41	NS	CYP3A (metabolism)	74.12	65.97	[372,373], FDA label
Riociguat	Negative on C_{max}	−0.83	0.24	CYP1A1, 2J2, 3A4 and 3A5, UGT1A1, UGT1A9 (metabolism), P−gp, BCRP (GI efflux), OCT2	−34.91	−11.63	[374]
Ritonavir	Negative on C_{max}	2.50	5.55	P−gp (GI efflux) and CYP3A4 (metabolism)	−26.67	−23.4	[375–377]
Rivaroxaban	Positive	0.90	1.84	CYP3A4/5, CYP2J2 (metabolism), P−gp, BCRP (GI efflux)	25	25	[378,379]
Rosuvastatin	Negative	−0.40	0.83	BCRP (GI efflux)	−31.03	−37.16	[380]
Rufinamide	Positive	0.57	NS	CES (metabolism)	95.89	42.83	[381,382]
Rupatadine	None	1.17	NS	CYP3A4/2C9/2C19/2D6 (High first pass metabolism)	−5.9	23.03	[383]
Sacubitril (Prodrug)	Negative on C_{max}	2.93	19.44	UGT2B17, CES1 (metabolism), P−gp (GI efflux)	−73.12	−21	[384,385]
Sacubitrilat (sacubitril metabolite)	Negative on C_{max}	2.35	20.87	OATP1B1 and OATP1B3, P−gp (GI efflux)	−27.13	−7.89	[384,385]

Safinamide	None	1.31	6.62	Cytosolic amidases, MAO−A, ALDH, CYP3A4 (metabolism), OAT3, BCRP (GI efflux)	NA	NA	[386]
Sapropterin	Positive	−0.86	11.61	P−gp (GI efflux), CYP3A4, and CYP2D1 (metabolism)	25	34.52	[387]
Saquinavir	Positive on AUC	2.99	35.78	CYP3A4 (metabolism), P−gp, MRP1/2 (GI efflux)	NA	570.83	[388]
Saroglitazar	Negative on C_{max}	1.03	0.36	P−gp (GI efflux) and CYP3A4 (metabolism)	−33.46	−3.82	[389,390]
Selegiline Hydrochloride	Positive	0.20	NS	CYP2B6 and CYP3A4, 2A6 (metabolism)	228.13	305.36	[391,392]
Selexipag (ACT−333679)	Negative on C_{max}	−0.43	0.03	CES1, CYP2C8, CYP3A4, UGT1A3, UGT2B7 (metabolism), OATP1B1, OATP1B3, P−gp/BCRP (GI efflux)	−35	1.1	[393]
Selumetinib	Negative on C_{max}	0.85	6.55	CYP2C19, UGT1A1 (metabolism), BCRP (GI efflux)	−50.28	−16.11	[394,395]
Sertindole	None	0.22	NS	CYP2D6/3A4 (metabolism)	NA	NA	[396,397]
Sildenafil	Negative on C_{max}	−0.94	NS	CYPs	−29.18	−11.27	[398]
Sofosbuvir (GS−331007)	Negative on C_{max}	0.29	30.22	CES1 (metabolism), P−gp and/or BCRP (GI efflux)	−25	NA	[399], FDA label
Solithromycin	None	1.98	18.93	CYP3A4 (metabolism), P−gp (GI efflux)	NA	NA	[400,401]
Sotalol	Negative	−0.09	NS	No metabolism	−25	−25	[402]
SRT−2104	Positive	2.48	NS	NA	249	273.53	[403]
Sulpiride	Positive	−0.13	11.72	PEPT1 (GI uptake), OCT1, OCT2, MATE1, and MATE2−K	25	25	[404]
Sunitinib maleate	None	0.81	3.76	CYP3A4 (metabolism),	9.96	18.63	[405,406]

				P−gp, BCRP (GI efflux)			
Tacrolimus	Negative	0.70	0.25	CYP3A (metabolism), P−gp/BCRP (GI efflux)	−77.03	−33.45	[407,408]
Tadalafil	None	−0.49	2.05	CYP3A4 (metabolism), P−gp (GI efflux)	16.16	8.16	16487221
Talazoparib	Negative on C_{max}	−1.70	0.05	Minimal metabolism, P−gp, BCRP (GI efflux)	−46	NA	[409]
Tedizolid	Negative on C_{max}	0.80	NS	SULT1A1, SULT1A2, SULT2A1 (metabolism)	−26.56	2.38	[410,411]
Tegafur/Ftorafur	Negative on C_{max}	−1.24	NS	CYP2A6 (metabolism), dihydropyrimidine dehydrogenase (DPD) (catabolic pathway), MRP8 (pepsinogen secretion)	−33.23	−8.76	[3]
Telaprevir	Positive on AUC	1.93	44.13	CYP3A4 (extensive metabolism), P−gp (GI efflux)	NA	330	[58,412]
Telithromycin	None	2.05	39.41	CYP3A4 (metabolism), P−gp, MRP2 (GI efflux)	NA	NA	[413,414]
Tenoxicam	None	−0.51	NS	CYP2C9/3A4 (metabolism)	NA	NA	[415]
Terbinafine	None	3.13	34.31	CYP2C9/1A2/3A4/2C8/2C19 (metabolism), P−gp (GI efflux)	21.29	22.09	[416–418]
Terfenadine	Positive	2.72	NS	CYP3A4 (metabolism)	25	25	[419]
Testosterone undecanoate	Positive	3.64	NS	Extensive metabolism, lymphatic transport	34960.24	27842.58	[420,421]
Tetracycline	Negative on C_{max}	0.18	45.00	P−gp (GI efflux)	−50	NA	[422]
Tetrahydrocannabinol	Positive	1.18	NS	CYPs 2C9, 3A4, UGTs (metabolism)	25	25	[423]
Theophylline	Negative	−0.80	199.82	CYP1A2 (extensive metabolism),	−59	−47	[424,425]

				PEPT1 (GI uptake), P−gp (GI efflux)			
Ticlopidine hydrochloride	None	1.66	NS	CYP3A4, CYP2C19, and CYP2B6 (metabolism)	21.29	20	[426–428]
Tipranavir	Positive on AUC	3.59	NS	CYP3A (extensive metabolism)	16	31	[58]
Tocotrienols (Vitamin E)	Positive	3.18	NS	CYP−mediated omega oxidation (CYP4F2) (metabolism), cholesterol transporter Niemann−Pick C1−like 1 (NPC1L1)	216	175.86	[429–431]
Tofacitinib	None	−0.83	1.41	CYP3A4/2C19 (metabolism), P−gp (GI efflux)	24.16	0.5	[432,433]64803, FDA label
Tolterodine	Positive	0.18	NS	CYP2D6 (extensive metabolism)	43	33.33	[434,435]
Topiramate	None	−1.23	11.79	CYP3A4 (metabolism), P−gp (GI efflux)	10.43	4.18	
Topotecan	None	−1.69	0.41	Hydrolysis (metabolism), P−gp/BCRP (GI efflux)	15.8	13.92	[436–438]
Torsemide	Negative on C_{max}	0.83	NS	CYP2C9 (extensive metabolism), OATP	−33	−2	[439]
Trametinib	Negative on C_{max}	−0.58	0.13	CES (metabolism), P−gp/BCRP (GI efflux)	−68.42	−15.26	[440,441], FDA label
Treprostinil	Positive on AUC	−0.26	NS	CYP2C8, UGTs (metabolism)	13	49	[442,443]
Troglitazone	Positive	3.12	36.24	Sulfation, glucuronidation (extensive metabolism), BCRP (GI efflux)	100	58.33	[444,445]
Trovafloxacin	None	1.06	19.21	UGT, CYP, SULT (metabolism), P−gp (GI efflux)	11.53	3.29	[446,447]
Tyramine	Negative	−0.85	NS	MAO−A, FMO3 , PNMT, DBH,	−78.16	−68.92	[448,449]

				and CYP2D6 (metabolism)			
Udenafil	None	1.00	15.48	CYP3A4 (metabolism) and P−gp (GI efflux)	20.26	3.9	[450,451]
Uracil	Negative	−1.52	NS	CYP2A6 (metabolism), sodium−dependent nucleobase transporter (rSNBT1)	−75.84	−34.92	[3,452]
Valsartan	Negative	1.14	7.35	OAT1, OAT3, OATP1B1, OATP1B3, MRP2 (GI efflux)	−40	−50	26225262
Vardenafil	None	−0.91	0.82	CYP3A4/3A5/2C (metabolism), P-gp, BCRP and MRP2 (GI efflux)	NA	NA	[453–455]
Veliparib	None	−0.24	6.55	CYPs (metabolism), P−gp (GI efflux)	17.64	4.08	[456–459]
Vemurafenib	Positive	4.03	78.38	CYP3A4 (metabolism), P−gp, BCRP (GI efflux)	114.28	184.48	[460,461]
Venetoclax	Positive	2.33	NS	CYP3A4 (extensive metabolism)	300	352	[462], FDA label
Venlafaxine	None	−0.06	7.21	CYP2D6 (metabolism), P−gp (GI efflux)	NA	NA	[463]
Verinurad	Negative on C_{max}	1.23	2.30	UGT2B17 (metabolism), CYP3A, P−gp (GI efflux)	−52.86	−23.14	[464–466]
Vinorelbine	Negative	1.59	6.16	CYP3A4 (extensive metabolism), MRP2 (GI efflux)	−34.99	−33.64	[467,468]
Voriconazole	Negative	0.20	45.80	CYP2C19, CYP3A4, CYP2C9 (metabolism), P−gp (GI efflux)	−60.56	−43.1	[469–471]
Zafirlukast	Negative	2.32	NS	CYP3A4, CYP2C9 (extensive metabolism)	−25	−25	[472,473]
Zalcitabine	Negative on C_{max}	−3.07	NS	Metabolism, OAT1	−38.49	−13.89	[474–476]

Zileuton	Positive on C_{max}	1.65	NS	CYP1A2, CYP2C9 and CYP3A4 (metabolism)	27	1.4	[477,478]
Zimelidine	None	−0.78	NS	Extensive first pass metabolism	NA	NA	[107,479]
Ziprasidone	Positive	1.65	NS	CYP3A4, CYP1A2 (metabolism)	84	104	[480,481]
Zolmitriptan	None	−1.28	0.35	CYP1A2 (metabolism), P−gp (GI efflux)	15.85	12	[482,483]
Zolpidem	Negative on C_{max}	−0.35	0.46	CYP3A4, CYP2C9, CYP1A2, CYP2D6, CYP2C19 (metabolism), P−gp (GI efflux)	−44.15	12.02	[484–486]
Zuclopenthixol	Positive on AUC	1.79	NS	Sulfoxidation, side chain N−dealkylation and glucuronic acid conjugation, CYP2D6 (metabolism)	NA	26	[487,488]
Olanzapine	None	−0.67	7.71	Metabolism, P−gp (GI efflux)	−19.75	−3.49	[489–491]
Warfarin	Negative on C_{max}	−0.23	NS	CYP2C9, 1A2, 2C19, 3A4, 2C8, 2C18 (metabolism)	−47.61	−7.12	[492]
Acebutolol	None	0.79	18.72	P−gp (GI efflux)	−20.73	−5.65	[493]
Pantoprazole	Negative on AUC	−0.49	4.17	Metabolism, P−gp (GI efflux)	−4.78	−33.34	[494]
Rabeprazole	None	−0.62	NS	Metabolism	21.46	7.76	[494]
Omeprazole	Negative on C_{max}	−0.35	4.63	P−gp	−26.67	−11.98	[494]
Peficitinib	Positive	0.58	NS	Metabolism	56.05	34.77	[495]

NA: not available; NS: non−substrate.

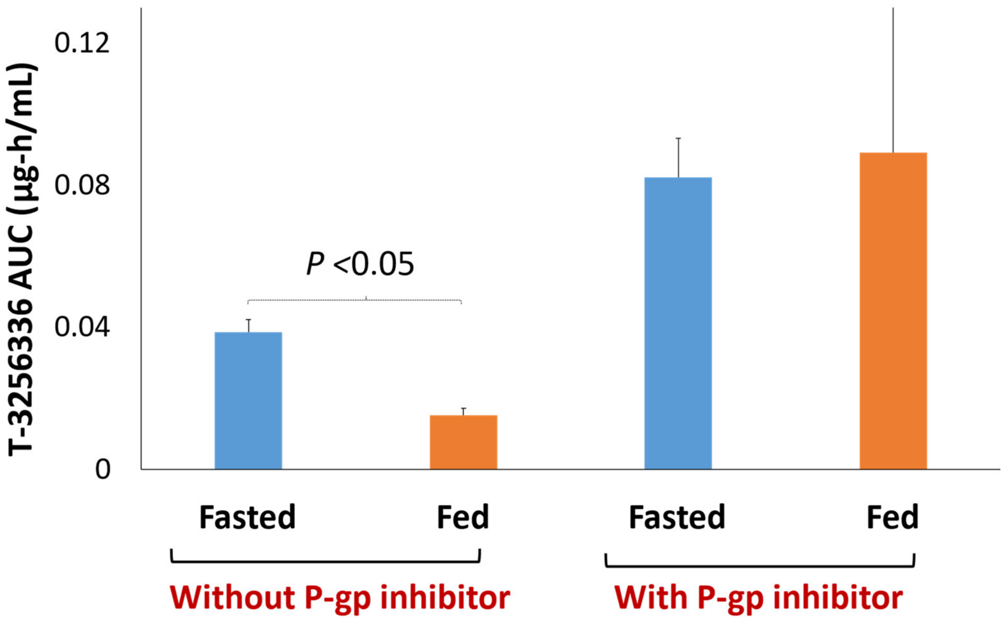

Figure S1. P-gp-dependent food-effect due to prolonged gastric emptying time and increased efficiency of P-gp efflux in the fed state. This mechanism is exemplified in the literature [496].

References

1. Yu, D.K.; Elvin, A.T.; Morrill, B.; Eichmeier, L.S.; Lanman, R.C.; Lanman, M.B.; Giesing, D.H. Effect of Food Coadministration on 5-Aminosalicylic Acid Oral Suspension Bioavailability. Clin Pharmacol Ther 1990, 48, 26–33.
2. Bondesen, S.; Hegnhøj, J.; Larsen, F.; Hansen, S.H.; Hansen, C.P.; Rasmussen, S.N. Pharmacokinetics of 5-Aminosalicylic Acid in Man Following Administration of Intravenous Bolus and per Os Slow-Release Formulation. Dig Dis Sci 1991, 36, 1735–1740.
3. Damle, B.; Ravandi, F.; Kaul, S.; Sonnichsen, D.; Ferreira, I.; Brooks, D.; Stewart, D.; Alberts, D.; Pazdur, R. Effect of Food on the Oral Bioavailability of UFT and Leucovorin in Cancer Patients. Clin Cancer Res 2001, 7, 517–523.
4. Burton, N.K.; Barnett, M.J.; Aherne, G.W.; Evans, J.; Douglas, I.; Lister, T.A. The Effect of Food on the Oral Administration of 6-Mercaptopurine. Cancer Chemother Pharmacol 1986, 18, 90–91.
5. Chittick, G.E.; Gillotin, C.; McDowell, J.A.; Lou, Y.; Edwards, K.D.; Prince, W.T.; Stein, D.S. Abacavir: Absolute Bioavailability, Bioequivalence of Three Oral Formulations, and Effect of Food. Pharmacotherapy 1999, 19, 932–942.
6. Yuen, G.J.; Weller, S.; Pakes, G.E. A Review of the Pharmacokinetics of Abacavir. Clin Pharmacokinet 2008, 47, 351–371.
7. Papangelou, A.; Olszanski, A.J.; Stein, C.A.; Bosch, B.; Nemeth, P. The Effect of Food on the Absorption of Abiraterone Acetate from a Fine Particle Dosage Form: A Randomized Crossover Trial in Healthy Volunteers. Oncology and Therapy 2017, 5, 161–170.
8. Othman, A.A.; Nothaft, W.; Awni, W.M.; Dutta, S. Pharmacokinetics of the TRPV1 Antagonist ABT-102 in Healthy Human Volunteers: Population Analysis of Data from 3 Phase 1 Trials. J Clin Pharmacol 2012, 52, 1028–1041.
9. Othman, A.A.; Cheskin, H.; Locke, C.; Nothaft, W.; Dutta, S. A Phase 1 Study to Evaluate the Bioavailability and Food Effect of 2 Solid-Dispersion Formulations of the TRPV1 Antagonist ABT-102, Relative to the Oral Solution Formulation, in Healthy Human Volunteers. Clin Pharmacol Drug Dev 2012, 1, 24–31.
10. Scherrer, D.; Rouzier, R.; Noel Barrett, P.; Steens, J.M.; Gineste, P.; Murphy, R.L.; Tazi, J.; Ehrlich, H.J. Pharmacokinetics and Tolerability of ABX464, a Novel First-in-Class Compound to Treat HIV Infection, in Healthy HIV-Uninfected Subjects. J Antimicrob Chemother 2017, 72, 820–828.
11. Gregoire, N.; Hovsepian, L.; Gualano, V.; Evene, E.; Dufour, G.; Gendron, A. Safety and Pharmacokinetics of Paracetamol Following Intravenous Administration of 5 g during the First 24 h with a 2-g Starting Dose. Clin Pharmacol Ther 2007, 81, 401–405.
12. Liu, D.J.; Collaku, A.; Youngberg, S.P. Bioavailability and Pharmacokinetic Profile of a Newly-Developed Twice-a-Day Sustained-Release Paracetamol Formulation. Int J Clin Pharmacol Ther 2015, 53, 172–181.
13. Efthymiopoulos C; Benedetti MS; Poggesi I; Ruff F; Basileo G; Musatti L Pharmacokinetics of Acipimox and of Its N-Deoxy Metabolite Following Single and Repeated Oral Administration to Healthy Volunteers. Therapie 1993, 48, 23–26.
14. Musatti, L.; Maggi, E.; Moro, E.; Valzelli, G.; Tamassia, V. Bioavailability and Pharmacokinetics in Man of Acipimox, a New Antilipolytic and Hypolipemic Agent. J Int Med Res 1981, 9, 381–386.
15. Fleishaker, J.C.; Phillips, J.P.; Lau, H.S.H. Effect of Food on the Bioavailability of Adinazolam from a Sustained Release Formulation: Effect of Meal Timing and Lack of Dose Dumping. Biopharm Drug Dispos 1990, 11, 715–727.

16. van Hoppe, S.; Sparidans, R.W.; Wagenaar, E.; Beijnen, J.H.; Schinkel, A.H. Breast Cancer Resistance Protein (BCRP/ABCG2) and P-Glycoprotein (P-Gp/ABCB1) Transport Afatinib and Restrict Its Oral Availability and Brain Accumulation. Pharmacol Res 2017, 120, 43–50.
17. Stopfer, P.; Marzin, K.; Narjes, H.; Gansser, D.; Shahidi, M.; Uttereuther-Fischer, M.; Ebner, T. Afatinib Pharmacokinetics and Metabolism after Oral Administration to Healthy Male Volunteers. Cancer Chemother Pharmacol 2012, 69, 1051–1061.
18. Wind, S.; Schnell, D.; Ebner, T.; Freiwald, M.; Stopfer, P. Clinical Pharmacokinetics and Pharmacodynamics of Afatinib. Clin Pharmacokinet 2017, 56, 235–250.
19. Ceballos, L.; Krolewiecki, A.; Juárez, M.; Moreno, L.; Schaer, F.; Alvarez, L.I.; Cimino, R.; Walson, J.; Lanusse, C.E. Assessment of Serum Pharmacokinetics and Urinary Excretion of Albendazole and Its Metabolites in Human Volunteers. PLoS Negl Trop Dis 2018, 12.
20. Nagy J; Schipper H G; Koopmans R P; Butter J J; van Boxtel C J; Kager P A Effect of Grapefruit Juice or Cimetidine Coadministration on Albendazole Bioavailability. Am J Trop Med Hyg 2002, 66, 260–263.
21. Nagy, J.; Schipper, H.G.; Koopmans, R.P.; Butter, J.J.; van Boxtel, C.J.; Kager, P.A. Effect of Grapefruit Juice or Cimetidine Coadministration on Albendazole Bioavailability. Am J Trop Med Hyg 2002, 66, 260–263.
22. Parrott, N.J.; Yu, L.J.; Takano, R.; Nakamura, M.; Morcos, P.N. Physiologically Based Absorption Modeling to Explore the Impact of Food and Gastric PH Changes on the Pharmacokinetics of Alectinib. AAPS J 2016, 18, 1464–1474.
23. Morcos, P.N.; Guerini, E.; Parrott, N.; Dall, G.; Blotner, S.; Bogman, K.; Sturm, C.; Balas, B.; Martin-Facklam, M.; Phipps, A. Effect of Food and Esomeprazole on the Pharmacokinetics of Alectinib, a Highly Selective ALK Inhibitor, in Healthy Subjects. Clin Pharmacol Drug Dev 2017, 6, 388–397.
24. Schmitt-Hoffmann, A.H.; Roos, B.; Sauer, J.; Schleimer, M.; Kovacs, P.; Stoeckel, K.; Maares, J. Influence of Food on the Pharmacokinetics of Oral Alitretinoin (9-Cis Retinoic Acid). Clin Exp Dermatol 2011, 36, 18–23.
25. Smith, R.B.; Kroboth, P.D.; Vanderlugt, J.T.; Phillips, J.P.; Juhl, R.P. Pharmacokinetics and Pharmacodynamics of Alprazolam after Oral and IV Administration. Psychopharmacology 1984, 84, 452–456.
26. Erdman, K.; Stypinski, D.; Combs, M.; Witt, P.; Stiles, M.; Pollock, S. Absence of Food Effect on the Extent of Alprazolam Absorption from an Orally Disintegrating Tablet. Pharmacotherapy 2007, 27, 1120–1124.
27. Haroldsen, P.E.; Garovoy, M.R.; Musson, D.G.; Zhou, H.; Tsuruda, L.; Hanson, B.; O'Neill, C.A. Genetic Variation in Aryl N-Acetyltransferase Results in Significant Differences in the Pharmacokinetic and Safety Profiles of Amifampridine (3,4-Diaminopyridine) Phosphate. Pharmacol Res Perspect 2015, 3, 1–13.
28. Haroldsen, P.E.; Sisic, Z.; Datt, J.; Musson, D.G.; Ingenito, G. Acetylator Status Impacts Amifampridine Phosphate (FirdapseTM) Pharmacokinetics and Exposure to a Greater Extent Than Renal Function. Clin Ther 2017, 39, 1360–1370.
29. Haroldsen, P.E.; Musson, D.G.; Hanson, B.; Quartel, A.; O'Neill, C.A. Effects of Food Intake on the Relative Bioavailability of Amifampridine Phosphate Salt in Healthy Adults. Clin Ther 2015, 37, 1555–1563.
30. Latini, R.; Tognoni, G.; Kates, R.E. Clinical Pharmacokinetics of Amiodarone. Clin Pharmacokinet 1984, 9, 136–156.
31. Meng, X.; Mojaverian, P.; Doedée, M.; Lin, E.; Weinryb, I.; Chiang, S.T.; Kowey, P.R. Bioavailability of Amiodarone Tablets Administered with and without Food in Healthy Subjects. Am J Cardiol 2001, 87, 432–435.
32. Liedholm, H.; Lidén, A. Food Intake and the Presystemic Metabolism of Single Doses of Amitriptyline and Nortriptyline. Fundam Clin Pharmacol 1998, 12, 636–642.
33. Liu, Y.; Jia, J.; Liu, G.; Li, S.; Lu, C.; Liu, Y.; Yu, C. Pharmacokinetics and Bioequivalence Evaluation of Two Formulations of 10-Mg Amlodipine Besylate: An Open-Label, Single-Dose, Randomized, Two-Way Crossover Study in Healthy Chinese Male Volunteers. Clin Ther 2009, 31, 777–783.
34. Faulkner J K; Hayden M L; Chasseaud L F; Taylor T Absorption of Amlodipine Unaffected by Food. Solid Dose Equivalent to Solution Dose. Arzneimittelforschung 1989, 39, 799–801.
35. Lecaillon, J.; Dubois, J.; Soula, G.; Pichard, E.; Poltera, A.; Ginger, C. The Influence of Food on the Pharmacokinetics of CGP 6140 (Amocarzine) after Oral Administration of a 1200 Mg Single Dose to Patients with Onchocerciasis. Br J Clin Pharmacol 1990, 30, 629–633.
36. Lecaillon, J.; Dubois, J.; Awadzi, K.; Poltera, A.; Ginger, C. Pharmacokinetics of CGP 6140 (Amocarzine) after Oral Administration of Single 100-1600 Mg Doses to Patients with Onchocerciasis. Br J Clin Pharmacol 1990, 30, 625–628.
37. Eshelman, F.N.; Spyker, D.A. Pharmacokinetics of Amoxicillin and Ampicillin. Crossover Study of the Effect of Food. Antimicrob Agents Chemother 1978, 14, 539–543.
38. Le, J.; Poindexter, B.; Sullivan, J.E.; Laughon, M.; Delmore, P.; Blackford, M.; Yogev, R.; James, L.P.; Melloni, C.; Harper, B.; et al. Comparative Analysis of Ampicillin Plasma and Dried Blood Spot Pharmacokinetics in Neonates. Ther Drug Monit 2018, 40, 103–108.
39. Byon, W.; Garonzik, S.; Boyd, R.A.; Frost, C.E. Apixaban: A Clinical Pharmacokinetic and Pharmacodynamic Review. Clin Pharmacokinet 2019, 58, 1265–1279.
40. Song, Y.; Chang, M.; Suzuki, A.; Frost, R.J.A.; Kelly, A.; LaCreta, F.; Frost, C. Evaluation of Crushed Tablet for Oral Administration and the Effect of Food on Apixaban Pharmacokinetics in Healthy Adults. Clin Ther 2016, 38, 1674-1685.e1.
41. Majumdar, A.K.; Howard, L.; Goldberg, M.R.; Hickey, L.; Constanzer, M.; Rothenberg, P.L.; Crumley, T.M.; Panebianco, D.; Bradstreet, T.E.; Bergman, A.J.; et al. Pharmacokinetics of Aprepitant after Single and Multiple Oral Doses in Healthy Volunteers. J Clin Pharmacol 2006, 46, 291–300.

42. Shono, Y.; Jantratid, E.; Kesisoglou, F.; Reppas, C.; Dressman, J.B. Forecasting in Vivo Oral Absorption and Food Effect of Micronized and Nanosized Aprepitant Formulations in Humans. Eur J Pharm Biopharm 2010, 76, 95–104.
43. Dien, T.K.; de Vries, P.J.; Khanh, N.X.; Koopmans, R.; Binh, L.N.; Duc, D.D.; Kager, P.A.; van Boxtel, C.J. Effect of Food Intake on Pharmacokinetics of Oral Artemisinin in Healthy Vietnamese Subjects. Antimicrob Agents Chemother 1997, 41, 1069–1072.
44. Schoepfer, J.; Jahnke, W.; Berellini, G.; Buonamici, S.; Cotesta, S.; Cowan-Jacob, S.W.; Dodd, S.; Drueckes, P.; Fabbro, D.; Gabriel, T.; et al. Discovery of Asciminib (ABL001), an Allosteric Inhibitor of the Tyrosine Kinase Activity of BCR-ABL1. J Med Chem 2018, 61, 8120–8135.
45. Menssen, H.D.; Quinlan, M.; Kemp, C.; Tian, X. Relative Bioavailability and Food Effect Evaluation for 2 Tablet Formulations of Asciminib in a 2-Arm, Crossover, Randomized, Open-Label Study in Healthy Volunteers. Clin Pharmacol Drug Dev 2019, 8, 385–394.
46. le Tiec, C.; Barrail, A.; Goujard, C.; Taburet, A.M. Clinical Pharmacokinetics and Summary of Efficacy and Tolerability of Atazanavir. Clin Pharmacokinet 2005, 44, 1035–1050.
47. Melander, A.; Stenberg, P.; Liedholm, H.; Scherstén, B.; Wåhlin-Boll, E. Food-Induced Reduction in Bioavailability of Atenolol. Eur J Clin Pharmacol 1979, 16, 327–330.
48. Lennernäs, H. Clinical Pharmacokinetics of Atorvastatin. Clin Pharmacokinet 2003, 42, 1141–1160.
49. Radulovic, L.L.; Cilla, D.D.; Posvar, E.L.; Sedman, A.J.; Whitfield, L.R. Effect of Food on the Bioavailability of Atorvastatin, an HMG-CoA Reductase Inhibitor. J Clin Pharmacol 1995, 35, 990–994.
50. Beermann, B.; Groschinsky-Grind, M.; Lindström, B. Pharmacokinetics of Bendroflumethiazide. Clin Pharmacol Ther 1977, 22, 385–388.
51. Beermann, B.; Groschinsky-Grind, M.; Lindström, B. Effect of Food on the Bioavailability of Bendroflumethiazide. Acta Med Scand 1978, 204, 291–293.
52. Toothaker, R.D.; Randinitis, E.J.; Nelson, C.; PhD, A.W.K.; Goulet, J.R. The Influence of Food on the Oral Absorption of Bevantolol. J Clin Pharmacol 1987, 27, 297–299.
53. Pao, L.H.; Zhou, S.Y.; Cook, C.; Kararli, T.; Kirchhoff, C.; Truelove, J.; Karim, A.; Fleisher, D. Reduced Systemic Availability of an Antiarrhythmic Drug, Bidisomide, with Meal Co-Administration: Relationship with Region-Dependent Intestinal Absorption. Pharm Res 1998, 15, 221–227.
54. Chen, X.; Wang, H.; Jiang, J.; Chen, R.; Zhou, Y.; Zhong, W.; Liu, H.; Hu, P. The Pharmacokinetic and Safety Profiles of Blonanserin in Healthy Chinese Volunteers after Single Fasting Doses and Single and Multiple Postprandial Doses. Clin Drug Investig 2014, 34, 213–222.
55. Saruwatari, J.; Yasui-Furukori, N.; Inoue, Y.; Kaneko, S. Effect of Dose Timing in Relation to Food Intake on Systemic Exposure to Blonanserin. Eur J Clin Pharmacol 2010, 66, 899–902.
56. Marathe, P.; Tang, Y.; Sleczka, B.; Rodrigues, D.; Gavai, A.; Wong, T.; Christopher, L.; Zhang, H. Preclinical Pharmacokinetics and in Vitro Metabolism of BMS-690514, a Potent Inhibitor of EGFR and VEGFR2. J Pharm Sci 2010, 99, 3579–3593.
57. Vakkalagadda, B.; Park, J.S.; Ahlers, C.M.; Dorizio, S.; Has, T.; Roongta, V.; Heller, K.N.; Derbin, G.M.; Zhang, S. Food Increased the Bioavailability of BMS-690514, an Orally Active EGFR/HER2/VEGF Receptor Kinase Inhibitor, in Healthy Subjects. J Clin Pharmacol 2012, 52, 1350–1356.
58. Impact of Food/Meal on Antiretroviral Drug Absorption Available online: https://hivclinic.ca/main/drugs_extra_files/Food%20impact%20on%20ARV%20PK.pdf (accessed on 28 May 2021).
59. Weber, C.; Schmitt, R.; Birnboeck, H.; Hopfgartner, G.; van Marie, S.P.; Peeters, P.A.M.; Jonkman, J.H.G.; Jones, C.R. Pharmacokinetics and Pharmacodynamics of the Endothelin-Receptor Antagonist Bosentan in Healthy Human Subjects. Clin Pharmacol Ther 1996, 60, 124–137.
60. Dingemanse, J.; Bodin, F.; Weidekamm, E.; Kutz, K.; van Giersbergen, P. Influence of Food Intake and Formulation on the Pharmacokinetics and Metabolism of Bosentan, a Dual Endothelin Receptor Antagonist. J Clin Pharmacol 2002, 42, 283–289.
61. Yamazaki, S.; Loi, C.M.; Kimoto, E.; Costales, C.; Varma, M. v. Application of Physiologically Based Pharmacokinetic Modeling in Understanding Bosutinib Drug-Drug Interactions: Importance of Intestinal P-Glycoprotein. Drug Metab Dispos 2018, 46, 1200–1211.
62. Skjodf, N.M.; Davies, N.M. Clinical Pharmacokinetics and Pharmacodynamics of Bromfenac. Clin Pharmacokinet 1999, 36, 399–408.
63. Fujii, J.; Inotsume, N.; Nakano, M. Effect of Food on the Bioavailability of Bromazepam Following Oral Administration in Healthy Volunteers. J Pharmacobiodyn 1990, 13, 269–271.
64. Maurer, G.; Schreier, E.; Delaborde, S.; Nufer, R.; Shukla, A.P. Fate and Disposition of Bromocriptine in Animals and Man. II: Absorption, Elimination and Metabolism. Eur J Drug Metab Pharmacokinet 1983, 8, 51–62.
65. Kopitar, Z.; Vrhovac, B.; Povšić, L.; Plavšić, F.; Francetić, I.; Urbančič, J. The Effect of Food and Metoclopramide on the Pharmacokinetics and Side Effects of Bromocriptine. Eur J Drug Metab Pharmacokinet 1991, 16, 177–181.
66. Mccrindle, J.L.; Li, T.C.; Wa, K.; Barron, W.; Prescott, L.F. Effect of Food on the Absorption of Frusemide and Bumetanide in Man. Br J Clin Pharmacol 1996, 42, 743–746.
67. Marcantonio, L.A.; Auld, W.H.R.; Skellern, G.G.; Howes, C.A.; Murdoch, W.R.; Purohit, R. The Pharmacokinetics and Pharmacodynamics of Bumetanide in Normal Subjects. J Pharmacokinet Biopharm 1982, 10, 393–409.

68. Lacy, S.; Hsu, B.; Miles, D.; Aftab, D.; Wang, R.; Nguyen, L. Metabolism and Disposition of Cabozantinib in Healthy Male Volunteers and Pharmacologic Characterization of Its Major Metabolites. Drug Metab Dispos 2015, 43, 1190–1207.
69. Nguyen, L.; Holland, J.; Mamelok, R.; Laberge, M.K.; Grenier, J.; Swearingen, D.; Armas, D.; Lacy, S. Evaluation of the Effect of Food and Gastric PH on the Single-Dose Pharmacokinetics of Cabozantinib in Healthy Adult Subjects. J Clin Pharmacol 2015, 55, 1293–1302.
70. Devineni, D.; Murphy, J.; Wang, S.S.; Stieltjes, H.; Rothenberg, P.; Scheers, E.; Mamidi, R.N.V.S. Absolute Oral Bioavailability and Pharmacokinetics of Canagliflozin: A Microdose Study in Healthy Participants. Clin Pharmacol Drug Dev 2015, 4, 295–304.
71. Gleiter, C.H.; Mörike, K.E. Clinical Pharmacokinetics of Candesartan. Clin Pharmacokinet 2002, 41, 7–17.
72. Simon, G.R.; Garrett, C.R.; Olson, S.C.; Langevin, M.; Eiseman, I.A.; Mahany, J.J.; Williams, C.C.; Lush, R.; Daud, A.; Munster, P.; et al. Increased Bioavailability of Intravenous versus Oral Cl-1033, a Pan ErbB Tyrosine Kinase Inhibitor: Results of a Phase I Pharmacokinetic Study. Clin Cancer Res 2006, 12, 4645–4651.
73. Millar, S.A.; Stone, N.L.; Yates, A.S.; O'Sullivan, S.E. A Systematic Review on the Pharmacokinetics of Cannabidiol in Humans. Front Pharmacol 2018, 9, 1365-undefined.
74. Singhvi, S.M.; McKinstry, D.N.; Shaw, J.M.; Willard, D.A.; Migdalof, B.H. Effect of Food on the Bioavailability of Captopril in Healthy Subjects. J Clin Pharmacol 1982, 22, 135–140.
75. Bauer, K.S.; Kohn, E.C.; Lush, R.M.; Steinberg, S.M.; Davis, P.; Kohler, D.; Reed, E.; Figg, W.D. Pharmacokinetics and Relative Bioavailability of Carboxyamido-Triazole with Respect to Food and Time of Administration: Use of a Single Model for Simultaneous Determination of Changing Parameters. J Pharmacokinet Biopharm 1998, 26, 673–687.
76. Tang, W.; McCormick, A.; Li, J.; Masson, E. Clinical Pharmacokinetics and Pharmacodynamics of Cediranib. Clin Pharmacokinet 2017, 56, 689–702.
77. Mitchell, C.L.; O'Connor, J.P.B.; Roberts, C.; Watson, Y.; Jackson, A.; Cheung, S.; Evans, J.; Spicer, J.; Harris, A.; Kelly, C.; et al. A Two-Part Phase II Study of Cediranib in Patients with Advanced Solid Tumours: The Effect of Food on Single-Dose Pharmacokinetics and an Evaluation of Safety, Efficacy and Imaging Pharmacodynamics. Cancer Chemother Pharmacol 2011, 68, 631–641.
78. Borin, M.T.; Forbes, K.K.; Hughes, G.S. The Bioavailability of Cefpodoxime Proxetil Tablets Relative to an Oral Solution. Biopharm Drug Dispos 1995, 16, 295–302.
79. Konishi, K.; Suzuki, H.; Hayashi, M.; Saruta, T. Pharmacokinetics of Cefuroxime Axetil in Patients with Normal and Impaired Renal Function. J Antimicrob Chemother 1993, 31, 413–420.
80. Finn, A.; Straughn, A.; Meyer, M.; Chubb, J. Effect of Dose and Food on the Bioavailability of Cefuroxime Axetil. Biopharm Drug Dispos 1987, 8, 519–526.
81. Davies, N.M.; McLachlan, A.J.; Day, R.O.; Williams, K.M. Clinical Pharmacokinetics and Pharmacodynamics of Celecoxib. A Selective Cyclo-Oxygenase-2 Inhibitor. Clin Pharmacokinet 2000, 38, 225–242.
82. Liston, D.R.; Davis, M. Clinically Relevant Concentrations of Anticancer Drugs: A Guide for Nonclinical Studies. Clin Cancer Res 2017, 23, 3489–3498.
83. Lau, Y.Y.; Gu, W.; Lin, T.; Song, D.; Yu, R.; Scott, J.W. Effects of Meal Type on the Oral Bioavailability of the ALK Inhibitor Ceritinib in Healthy Adult Subjects. J Clin Pharmacol 2016, 56, 559–566.
84. Moore, B.R.; Page-Sharp, M.; Stoney, J.R.; Ilett, K.F.; Jago, J.D.; Batty, K.T. Pharmacokinetics, Pharmacodynamics, and Allometric Scaling of Chloroquine in a Murine Malaria Model. Antimicrob Agents Chemother 2011, 55, 3899–3907.
85. Tulpule, A.; Krishnaswamy, K. Effect of Food on Bioavailability of Chloroquine. Eur J Clin Pharmacol 1982, 23, 271–273.
86. Jung, D.; Lam, H. di; Chu, M. Absorption and Disposition Kinetics of Chlorothiazide in Protein-calorie Malnutrition. Biopharm Drug Dispos 1990, 11, 53–60.
87. Welling, P.G.; Barbhaiya, R.H. Influence of Food and Fluid Volume on Chlorothiazide Bioavailability: Comparison of Plasma and Urinary Excretion Methods. J Pharm Sci 1982, 71, 32–35.
88. Massarella, J.W.; Blumenthal, H.P.; Silvestri, T.; Lin, A. Effect of Food on Cibenzoline Bioavailability. Eur J Clin Pharmacol 1986, 30, 367–369.
89. Williams, P.; Brown, A.; Rajaguru, S.; Francis, R.; Walters, G.; McEwen, J.; Durnin, C. The Pharmacokinetics and Bioavailability of Cilazapril in Normal Man. Br J Clin Pharmacol 1989, 27, 181S-188S.
90. Massarella, J.; DeFeo, T.; Brown, A.; Lin, A.; Wills, R. The Influence of Food on the Pharmacokinetics and ACE Inhibition of Cilazapril. Br J Clin Pharmacol 1989, 27, 205S-209S.
91. Bramer, S.L.; Forbes, W.P.; Mallikaarjun, S. Cilostazol Pharmacokinetics after Single and Multiple Oral Doses in Healthy Males and Patients with Intermittent Claudication Resulting from Peripheral Arterial Disease. Clin Pharmacokinet 1999, 37, 1–11.
92. Bramer, S.L.; Forbes, W.P. Relative Bioavailability and Effects of a High Fat Meal on Single Dose Cilostazol Pharmacokinetics. Clin Pharmacokinet 1999, 37, 13–23.
93. Christiansen, M.L.; Holm, R.; Abrahamsson, B.; Jacobsen, J.; Kristensen, J.; Andersen, J.R.; Müllertz, A. Effect of Food Intake and Co-Administration of Placebo Self-Nanoemulsifying Drug Delivery Systems on the Absorption of Cinnarizine in Healthy Human Volunteers. Eur J Pharm Sci 2016, 84, 77–82.
94. Hoffken, G.; Lode, H.; Prinzing, C.; Borner, K.; Koeppe, P. Pharmacokinetics of Ciprofloxacin after Oral and Parenteral Administration. Antimicrob Agents Chemother 1985, 27, 375–379.

95. Radwan, A.; Zaid, A.N.; Jaradat, N.; Odeh, Y. Food Effect: The Combined Effect of Media PH and Viscosity on the Gastrointestinal Absorption of Ciprofloxacin Tablet. Eur J Pharm Sci 2017, 101, 100–106.
96. Audet, P.R.; Chiang, S.; Maroli, A.; Locniskar, A.; Morrison, G. The Effect of Food on Ciramadol Bioavailability in Normal Subjects. Biopharm Drug Dispos 1987, 8, 299–304.
97. Conrad, K.A.; Lee, S.M. Clodronate Kinetics and Dynamics. Clin Pharmacol Ther 1981, 30, 114–120.
98. Laitinen, K.; Patronen, A.; Harju, P.; Löyttyniemi, E.; Pylkkänen, L.; Kleimola, T.; Perttunen, K. Timing of Food Intake Has a Marked Effect on the Bioavailability of Clodronate. Bone 2000, 27, 293–296.
99. Nirogi, R.V.S.; Kandikere, V.N.; Mudigonda, K. Effect of Food on Bioavailability of a Single Oral Dose of Clopidogrel in Healthy Male Subjects. Arzneimittelforschung 2006, 56, 735–739.
100. Cushing, D.J.; Souney, P.F.; Cooper, W.D.; Mosher, G.L.; Adams, M.P.; Machatha, S.; Zhang, B.; Kowey, P.R. Pharmacokinetics and Platelet Aggregation Inhibitory Effects of a Novel Intravenous Formulation of Clopidogrel in Humans. Clin Exp Pharmacol Physiol 2012, 39, 3–8.
101. Cheng, Y.F.; Lundberg, T.; Bondesson, U.; Lindström, L.; Gabrielsson, J. Clinical Pharmacokinetics of Clozapine in Chronic Schizophrenic Patients. Eur J Clin Pharmacol 1988, 34, 445–449.
102. DiSanto, A.R.; Golden, G. Effect of Food on the Pharmacokinetics of Clozapine Orally Disintegrating Tablet 12.5mg: A Randomized, Open-Label, Crossover Study in Healthy Male Subjects. Clin Drug Investig 2009, 29, 539–549.
103. Gaur, A.H.; Kizito, H.; Prasitsueubsai, W.; Rakhmanina, N.; Rassool, M.; Chakraborty, R.; Batra, J.; Kosalaraksa, P.; Luesomboon, W.; Porter, D.; et al. Safety, Efficacy, and Pharmacokinetics of a Single-Tablet Regimen Containing Elvitegravir, Cobicistat, Emtricitabine, and Tenofovir Alafenamide in Treatment-Naive, HIV-Infected Adolescents: A Single-Arm, Open-Label Trial. Lancet HIV 2016, 3, e561–e568.
104. Takahashi, R.H.; Choo, E.F.; Ma, S.; Wong, S.; Halladay, J.; Deng, Y.; Rooney, I.; Gates, M.; Hop, C.E.C.A.; Khojasteh, S.C.; et al. Absorption, Metabolism, Excretion, and the Contribution of Intestinal Metabolism to the Oral Disposition of [14C]Cobimetinib, a MEK Inhibitor, in Humans. Drug Metab Dispos 2016, 44, 28–39.
105. Musib, L.; Choo, E.; Deng, Y.; Eppler, S.; Rooney, I.; Chan, I.T.; Dresser, M.J. Absolute Bioavailability and Effect of Formulation Change, Food, or Elevated PH with Rabeprazole on Cobimetinib Absorption in Healthy Subjects. Mol Pharm 2013, 10, 4046–4054.
106. Yue, Q.; Hasselstrom, J.; Svensson, J.; Sawe, J. Pharmacokinetics of Codeine and Its Metabolites in Caucasian Healthy Volunteers: Comparisons between Extensive and Poor Hydroxylators of Debrisoquine. Br J Clin Pharmacol 1991, 31, 635–642.
107. Melander, A.; Mclean, A. Influence of Food Intake on Presystemic Clearance of Drugs. Clin Pharmacokinet 1983, 8, 286–296.
108. Xu, H.; O'Gorman, M.; Boutros, T.; Brega, N.; Kantaridis, C.; Tan, W.; Bello, A. Evaluation of Crizotinib Absolute Bioavailability, the Bioequivalence of Three Oral Formulations, and the Effect of Food on Crizotinib Pharmacokinetics in Healthy Subjects. J Clin Pharmacol 2015, 55, 104–113.
109. Kaniwa, N.; Ogata, H.; Aoyagi, N.; Ejima, A.; Takahashi, T.; Uezono, Y.; Imazato, Y. Effect of Food on the Bioavailability of Cyclandelate from Commercial Capsules. Clin Pharmacol Ther 1991, 49, 641–647.
110. Gupta, S.K.; Manfro, R.C.; Tomlanovich, S.J.; Gambertoglio, J.G.; Garovoy, M.R.; Benet, L.Z. Effect of Food on the Pharmacokinetics of Cyclosporine in Healthy Subjects Following Oral and Intravenous Administration. J Clin Pharmacol 1990, 30, 643–653.
111. Denton, C.L.; Minthorn, E.; Carson, S.W.; Young, G.C.; Richards-peterson, L.E.; Botbyl, J.; Han, C.; Morrison, R.A.; Blackman, S.C.; Ouellet, D. Concomitant Oral and Intravenous Pharmacokinetics of Dabrafenib, a BRAF Inhibitor, in Patients with BRAF V600 Mutation-Positive Solid Tumors. J Clin Pharmacol 2013, 53, 955–961.
112. Ouellet, D.; Grossmann, K.F.; Limentani, G.; Nebot, N.; Lan, K.; Knowles, L.; Gordon, M.S.; Sharma, S.; Infante, J.R.; Lorusso, P.M.; et al. Effects of Particle Size, Food, and Capsule Shell Composition on the Oral Bioavailability of Dabrafenib, a BRAF Inhibitor, in Patients with BRAF Mutation-Positive Tumors. J Pharm Sci 2013, 102, 3100–3109.
113. Hooper, W.D.; Eadie, M.J.; Dickinson, R.G. Single Oral Dose Pharmacokinetics and Comparative Bioavailability of Danazol in Humans. Biopharm Drug Dispos 1991, 12, 577–582.
114. Sunesen, V.H.; Vedelsdal, R.; Kristensen, H.G.; Christrup, L.; Müllertz, A. Effect of Liquid Volume and Food Intake on the Absolute Bioavailability of Danazol, a Poorly Soluble Drug. Eur J Pharm Sci 2005, 24, 297–303.
115. Thomas, N.K.; Anne B.; Frank L. T.; Tony V.; Tom Van De C.; Richard M. W. H. Pharmacokinetics and Pharmacodynamics of Boosted Once-Daily Darunavir - PubMed. J Antimicrob Chemother 2014, 69, 2591–2605.
116. Séchaud, R.; Robeva, A.; Belleli, R.; Balez, S. Absolute Oral Bioavailability and Disposition of Deferasirox in Healthy Human Subjects. J Clin Pharmacol 2008, 48, 919–925.
117. Galanello, R.; Piga, A.; Domenica Cappellini, M.; Luca Forni, G.; Zappu, A.; Origa, R.; Dutreix, C.; Belleli, R.; Ford, J.M.; Rivière, G.J.; et al. Effect of Food, Type of Food, and Time of Food Intake on Deferasirox Bioavailability: Recommendations for an Optimal Deferasirox Administration Regimen. J Clin Pharmacol 2008, 48, 428–435.
118. Möllmann, H.; Hochhaus, G.; Rohatagi, S.; Barth, J.; Derendorf, H. Pharmacokinetic/Pharmacodynamic Evaluation of Deflazacort in Comparison to Methylprednisolone and Prednisolone. Pharm Res 1995, 12, 1096–1100.
119. Rao, N.; Eller, M.; Arumugham, T.; Weir, S. The Effect of Food on the Relative Bioavailability of Deflazacort. Eur J Drug Metab Pharmacokinet 1996, 21, 241–245.

120. Drabant, S.; Balogh Nemes, K.; Horváth, V.; Tolokán, A.; Grézal, G.; Anttila, M.; Gachályi, B.; Kanerva, H.; Al-Behaisi, S.; Horvai, G.; et al. Influence of Food on the Oral Bioavailability of Deramciclane from Film-Coated Tablet in Healthy Male Volunteers. Eur J Pharm Biopharm 2004, 58, 689–695.
121. Yeh, G.C.; Deng, S.T.; Lo, C.Y.; Chiang, P.S.; Hsiong, C.H. Pharmacokinetics and Bioequivalence Study of a Generic Desloratadine Tablet Formulation in Healthy Male Volunteers. Arzneimittelforschung 2004, 54, 166–170.
122. Gupta, S.; Banfield, C.; Affrime, M.; Marbury, T.; Padhi, D.; Glue, P. Oral Bioavailability of Desloratadine Is Unaffected by Food. Clin Pharmacokinet 2002, 41, 7–12.
123. Agersø, H.; Larsen, L.S.; Riis, A.; Lövgren, U.; Karlsson, M.O.; Senderovitz, T. Pharmacokinetics and Renal Excretion of Desmopressin after Intravenous Administration to Healthy Subjects and Renally Impaired Patients. Br J Clin Pharmacol 2004, 58, 352–358.
124. Rittig S.; Jensen A. R.; Jensen K. T.; Pedersen E. B. Effect of Food Intake on the Pharmacokinetics and Antidiuretic Activity of Oral Desmopressin (DDAVP) in Hydrated Normal Subjects. Clin Endocrinol 1998, 48, 235–241.
125. Flanagan, R.; Johnston, A.; White, A.; Crome, P. Pharmacokinetics of Dextropropoxyphene and Nordextropropoxyphene in Young and Elderly Volunteers after Single and Multiple Dextropropoxyphene Dosage. Br J Clin Pharmacol 1989, 28, 463–469.
126. Chen, C.; Bujanover, S.; Kareht, S.; Rapoport, A.M. Differential Pharmacokinetics of Diclofenac Potassium for Oral Solution vs Immediate-Release Tablets from a Randomized Trial: Effect of Fed and Fasting Conditions. Headache 2015, 55, 265–275.
127. Melander, A.; Wåhlin, E. Enhancement of Dicoumarol Bioavailability by Concomitant Food Intake. Eur J Clin Pharmacol 1978, 14, 441–444.
128. Shyu, W.C.; Knupp, C.A.; Pittman, K.A.; Dunkle, L.; Barbhaiya, R.H. Food-induced Reduction in Bioavailability of Didanosine. Clin Pharmacol Ther 1991, 50, 503–507.
129. Singlas, E.; Taburet, A.M.; Lebas, F.B.; de Curzon, O.P.; Sobel, A.; Chauveau, P.; Viron, B.; Khayat, R.A.; Poignet, J.L.; Mignon, F.; et al. Didanosine Pharmacokinetics in Patients with Normal and Impaired Renal Function: Influence of Hemodialysis. Antimicrob Agents Chemother 1992, 36, 1519–1524.
130. Greenblatt, D.J.; Duhme, D.W.; Koch-Weser, J.; Smith Clinical, T.W. Bioavailability of Digoxin Tablets and Elixir in the Fasting and Postprandial States. Clin Pharmacol Ther 1974, 16, 444–448.
131. Trenk, D.; Wagner, F.; Sachs, W.; Jähnchen, E. Pharmacokinetic Characterization of the Antiarrhythmic Drug Diprafenone in Man. Eur J Clin Pharmacol 1989, 37, 313–316.
132. Koytchev, R.; Alken, R.G.; Mayer, O.; Smith, I.; Greenwood, M. Influence of Food on the Bioavailability and Some Pharmacokinetic Parameters of Diprafenone - A Novel Antiarrhythmic Agent. Eur J Clin Pharmacol 1996, 50, 315–319.
133. Lombardo, F.; Berellini, G.; Obach, R.S. Trend Analysis of a Database of Intravenous Pharmacokinetic Parameters in Humans for 1352 Drug Compounds. Drug Metab Dispos 2018, 46, 1466–1477.
134. Liedholm H.; Lidén A.; Kroon L.; Melander A.; Wåhlin-Boll E. Pharmacokinetics of Dixyrazine: Low Bioavailability, Improved by Food Intake. Drug Nutr Interact 1985, 3, 87–92.
135. Song, I.H.; Borland, J.; Savina, P.M.; Chen, S.; Patel, P.; Wajima, T.; Peppercorn, A.F.; Piscitelli, S.C. Pharmacokinetics of Single-Dose Dolutegravir in HIV-Seronegative Subjects With Moderate Hepatic Impairment Compared to Healthy Matched Controls. Clin Pharmacol Drug Dev 2013, 2, 342–348.
136. Weller S.; Chen S.; Borland J.; Savina P.; Wynne B.; Piscitelli S. C. Bioequivalence of a Dolutegravir, Abacavir, and Lamivudine Fixed-Dose Combination Tablet and the Effect of Food. J Acquir Immune Defic Syndr 2014, 66, 393–398.
137. Sanchez, R.I.; Fillgrove, K.L.; Yee, K.L.; Liang, Y.; Lu, B.; Tatavarti, A.; Liu, R.; Anderson, M.S.; Behm, M.O.; Fan, L.; et al. Characterisation of the Absorption, Distribution, Metabolism, Excretion and Mass Balance of Doravirine, a Non-Nucleoside Reverse Transcriptase Inhibitor in Humans. Xenobiotica 2019, 49, 422–432.
138. Behm, M.O.; Yee, K.L.; Liu, R.; Levine, V.; Panebianco, D.; Fackler, P. The Effect of Food on Doravirine Bioavailability: Results from Two Pharmacokinetic Studies in Healthy Subjects. Clin Drug Investig 2017, 37, 571–579.
139. Dubbelman, A.C.; Upthagrove, A.; Beijnen, J.H.; Marchetti, S.; Tan, E.; Krone, K.; Anand, S.; Schellens, J.H.M. Disposition and Metabolism of 14C-Dovitinib (TKI258), an Inhibitor of FGFR and VEGFR, after Oral Administration in Patients with Advanced Solid Tumors. Cancer Chemother Pharmacol 2012, 70, 653–663.
140. Sharma, S.; Britten, C.D.; Mortimer, J.; Kulkarni, S.; Quinlan, M.; Liu, A.; Scott, J.W.; George, D. The Effect of Formulation and Food Consumption on the Bioavailability of Dovitinib (TKI258) in Patients with Advanced Solid Tumors. Cancer Chemother Pharmacol 2014, 74, 867–874.
141. Deshmukh, P. v.; Badgujar, P.C.; Gatne, M.M. In-Vitro Red Blood Cell Partitioning of Doxycycline. Indian J Pharmacol 2009, 41, 173–175.
142. Agwuh, K.N.; MacGowan, A. Pharmacokinetics and Pharmacodynamics of the Tetracyclines Including Glycylcyclines. J Antimicrob Chemother 2006, 58, 256–265.
143. Welling, P.G.; Koch, P.A.; Lau, C.C.; Craig, W.A. Bioavailability of Tetracycline and Doxycycline in Fasted and Nonfasted Subjects. Antimicrob Agents Chemother 1977, 11, 462–469.
144. Naccarelli, G. v.; Wolbrette, D.L.; Levin, V.; Samii, S.; Banchs, J.E.; Penny-Peterson, E.; Gonzalez, M.D. Safety and Efficacy of Dronedarone in the Treatment of Atrial Fibrillation/Flutter. Clin Med Insights Cardiol 2011, 5, 103–119.
145. Knadler, M.P.; Lobo, E.; Chappell, J.; Bergstrom, R. Duloxetine: Clinical Pharmacokinetics and Drug Interactions. Clin Pharmacokinet 2011, 50, 281–294.

146. Rizea-Savu, S.; Duna, S.N.; Ghita, A.; Iordachescu, A.; Chirila, M. The Effect of Food on the Single-Dose Bioavailability and Tolerability of the Highest Marketed Strength of Duloxetine. Clin Pharmacol Drug Dev 2020, 9, 797–804.
147. Csajka, C.; Marzolini, C.; Fattinger, K.; Décosterd, L.A.; Fellay, J.; Telenti, A.; Biollaz, J.; Buclin, T. Population Pharmacokinetics and Effects of Efavirenz in Patients with Human Immunodeficiency Virus Infection. Clin Pharmacol Ther 2003, 73, 20–30.
148. Kaul, S.; Ji, P.; Lu, M.; Nguyen, K.L.; Shangguan, T.; Grasela, D. Bioavailability in Healthy Adults of Efavirenz Capsule Contents Mixed with a Small Amount of Food. Am J Health Syst Pharm 2010, 67, 217–222.
149. Deng, Y.; Madatian, A.; Wire, M.B.; Bowen, C.; Park, J.W.; Williams, D.; Peng, B.; Schubert, E.; Gorycki, F.; Levy, M.; et al. Metabolism and Disposition of Eltrombopag, an Oral, Nonpeptide Thrombopoietin Receptor Agonist, in Healthy Human Subjects. Drug Metab Dispos 2011, 39, 1734–1746.
150. Williams, D.D.; Peng, B.; Bailey, C.K.; Wire, M.B.; Deng, Y.; Park, J.W.; Collins, D.A.; Kapsi, S.G.; Jenkins, J.M. Effects of Food and Antacids on the Pharmacokinetics of Eltrombopag in Healthy Adult Subjects: Two Single-Dose, Open-Label, Randomized-Sequence, Crossover Studies. Clin Ther 2009, 31, 764–776.
151. Podany, A.T.; Scarsi, K.K.; Fletcher, C. v. Comparative Clinical Pharmacokinetics and Pharmacodynamics of HIV-1 Integrase Strand Transfer Inhibitors. Clin Pharmacokinet 2017, 56, 25–40.
152. Wolf, R.; Eberl, R.; Dunky, A.; Mertz, N.; Chang, T.; Goulet, J.R.; Latts, J. The Clinical Pharmacokinetics and Tolerance of Enoxacin in Healthy Volunteers. J Antimicrob Chemother 1984, 14, 63–69.
153. Zhang, Q.H.; Yang, J.; He, Y.; Liu, F.; Wang, J.P.; Davey, A.K. Food Effect on the Pharmacokinetics of Entecavir from Dispersible Tablets Following Oral Administration in Healthy Chinese Volunteers. Arzneimittelforschung 2010, 60, 640–644.
154. Yan, J.H.; Bifano, M.; Olsen, S.; Smith, R.A.; Zhang, D.; Grasela, D.M.; LaCreta, F. Entecavir Pharmacokinetics, Safety, and Tolerability after Multiple Ascending Doses in Healthy Subjects. J Clin Pharmacol 2006, 46, 1250–1258.
155. Tenero D.; Martin D.; Ilson B.; Jushchyshyn J.; Boike S.; Lundberg D.; Zariffa N.; Boyle D.; Jorkasky D. Pharmacokinetics of Intravenously and Orally Administered Eprosartan in Healthy Males: Absolute Bioavailability and Effect of Food. Biopharm Drug Dispos 1998, 19, 351–356.
156. White-Koning, M.; Civade, E.; Geoerger, B.; Thomas, F.; Deley, M.C. le; Hennebelle, I.; Delord, J.P.; Chatelut, E.; Vassal, G. Population Analysis of Erlotinib in Adults and Children Reveals Pharmacokinetic Characteristics as the Main Factor Explaining Tolerance Particularities in Children. Clin Cancer Res 2011, 17, 4862–4871.
157. Ling, J.; Fettner, S.; Lum, B.L.; Riek, M.; Rakhit, A. Effect of Food on the Pharmacokinetics of Erlotinib, an Orally Active Epidermal Growth Factor Receptor Tyrosine-Kinase Inhibitor, in Healthy Individuals. Anticancer Drugs 2008, 19, 209–216.
158. Sun, H.; Frassetto, L.A.; Huang, Y.; Benet, L.Z. Hepatic Clearance, but Not Gut Availability, of Erythromycin Is Altered in Patients with End-Stage Renal Disease. Clin Pharmacol Ther 2010, 87, 465–472.
159. Clayton D.; Leslie A. The Bioavailability of Erythromycin Stearate versus Enteric-Coated Erythromycin Base When Taken Immediately before and after Food. J Int Med Res 1981, 9, 470–477.
160. Bialer, M.; Soares-Da-Silva, P. Pharmacokinetics and Drug Interactions of Eslicarbazepine Acetate. Epilepsia 2012, 53, 935–946.
161. Fontes-Ribeiro, C.; MacEdo, T.; Nunes, T.; Neta, C.; Vasconcelos, T.; Cerdeira, R.; Lima, R.; Rocha, J.F.; Falcão, A.; Almeida, L.; et al. Dosage Form Proportionality and Food Effect of the Final Tablet Formulation of Eslicarbazepine Acetate: Randomized, Open-Label, Crossover, Single-Centre Study in Healthy Volunteers. Drugs R D 2008, 9, 447–454.
162. Maia, J.; Vaz-Da-Silva, M.; Almeida, L.; Falcão, A.; Silveira, P.; Guimarães, S.; Graziela, P.; Soares-Da-Silva, P. Effect of Food on the Pharmacokinetic Profile of Eslicarbazepine Acetate (BIA 2-093). Drugs R D 2005, 6, 201–206.
163. Gunnarsson, P.O.; Davidsson, T.; Andersson, S.B.; Backman, C.; Johansson, S.Å. Impairment of Estramustine Phosphate Absorption by Concurrent Intake of Milk and Food. Eur J Clin Pharmacol 1990, 38, 189–193.
164. Nguyen, P.T.T.; Parvez, M.M.; Kim, M.J.; Ho Lee, J.; Ahn, S.; Ghim, J.L.; Shin, J.G. Development of a Physiologically Based Pharmacokinetic Model of Ethionamide in the Pediatric Population by Integrating Flavin-Containing Monooxygenase 3 Maturational Changes Over Time. J Clin Pharmacol 2018, 58, 1347–1360.
165. Auclair, B.; Nix, D.E.; Adam, R.D.; James, G.T.; Peloquin, C.A. Pharmacokinetics of Ethionamide Administered under Fasting Conditions or with Orange Juice, Food, or Antacids. Antimicrob Agents Chemother 2001, 45, 810–814.
166. Sinkule J. A.; Hutson P.; Hayes F. A.; Etcubanas E.; Evans W. Pharmacokinetics of Etoposide (VP16) in Children and Adolescents with Refractory Solid Tumors. Cancer Res 1984, 44, 3109–3113.
167. Harvey, V.J.; Slevin, M.L.; Joel, S.P.; Johnston, A.; Wrigley, P.F.M. The Effect of Food and Concurrent Chemotherapy on the Bioavailability of Oral Etoposide. Br J Cancer 1985, 52, 363–367.
168. Takemoto, J.K.; Reynolds, J.K.; Remsberg, C.M.; Vega-Villa, K.R.; Davies, N.M. Clinical Pharmacokinetic and Pharmacodynamic Profile of Etoricoxib. Clin Pharmacokinet 2008, 47, 703–720.
169. Agrawal, N.G.B.; Porras, A.G.; Matthews, C.Z.; Rose, M.J.; Woolf, E.J.; Musser, B.J.; Dynder, A.L.; Mazina, K.E.; Lasseter, K.C.; Hunt, T.L.; et al. Single- and Multiple-Dose Pharmacokinetics of Etoricoxib, a Selective Inhibitor of Cyclooxygenase-2, in Man. J Clin Pharmacol 2003, 43, 268–276.
170. Cannady, E.A.; Aburub, A.; Ward, C.; Hinds, C.; Czeskis, B.; Ruterbories, K.; Suico, J.G.; Royalty, J.; Ortega, D.; Pack, B.W.; et al. Absolute Bioavailability of Evacetrapib in Healthy Subjects Determined by Simultaneous Administration of Oral Evacetrapib and Intravenous [13C8]-Evacetrapib as a Tracer. J Labelled Comp Radiopharm 2016, 59, 238–244.

171. Cannady, E.A.; Wang, M.D.; Friedrich, S.; Rehmel, J.L.F.; Yi, P.; Small, D.S.; Zhang, W.; Suico, J.G. Evacetrapib: In Vitro and Clinical Disposition, Metabolism, Excretion, and Assessment of Drug Interaction Potential with Strong CYP3A and CYP2C8 Inhibitors. Pharmacol Res Perspect 2015, 3.
172. Kovarik, J.M.; Hartmann, S.; Figueiredo, J.; Rordorf, C.; Golor, G.; Lison, A.; Budde, K.; Neumayer, H.H. Effect of Food on Everolimus Absorption: Quantification in Healthy Subjects and a Confirmatory Screening in Patients with Renal Transplants. Pharmacotherapy 2002, 22, 154–159.
173. Kirchner, G.I.; Meier-Wiedenbach, I.; Manns, M.P. Clinical Pharmacokinetics of Everolimus. Clin Pharmacokinet 2004, 43, 83–95.
174. Zhang, M.; Xu, C.; Ma, L.; Shamiyeh, E.; Yin, J.; von Moltke, L.L.; Smith, W.B. Effect of Food on the Bioavailability and Tolerability of the JAK2-Selective Inhibitor Fedratinib (SAR302503): Results from Two Phase I Studies in Healthy Volunteers. Clin Pharmacol Drug Dev 2015, 4, 315–321.
175. Yun, H.Y.; Eun, J.L.; Soo, Y.C.; Choi, S.O.; Hyung, K.K.; Kwon, J.T.; Kang, W.; Kwon, K. il The Effects of Food on the Bioavailability of Fenofibrate Administered Orally in Healthy Volunteers via Sustained-Release Capsule. Clin Pharmacokinet 2006, 45, 425–432.
176. Back, H. moon; Song, B.; Pradhan, S.; Chae, J. woo; Han, N.; Kang, W.; Chang, M.J.; Zheng, J.; Kwon, K. il; Karlsson, M.O.; et al. A Mechanism-Based Pharmacokinetic Model of Fenofibrate for Explaining Increased Drug Absorption after Food Consumption. BMC Pharmacol Toxicol 2018, 19.
177. Weber, R.; McCoy, C.; Ziemniak, J.; Frederickson, E.; Goldberg, L.; Murphy, M. Pharmacokinetic and Pharmacodynamic Properties of Intravenous Fenoldopam, a Dopamine1-receptor Agonist, in Hypertensive Patients. Br J Clin Pharmacol 1988, 25, 17–21.
178. Clancy, A.; Locke-Haydon, J.; Cregeen, R.J.; Ireson, M.; Ziemniak, J. Effect of Concomitant Food Intake on Absorption Kinetics of Fenoldopam (SK&F 82526) in Healthy Volunteers. Eur J Clin Pharmacol 1987, 32, 103–106.
179. Tarral, A.; Blesson, S.; Mordt, O.V.; Torreele, E.; Sassella, D.; Bray, M.A.; Hovsepian, L.; Evène, E.; Gualano, V.; Felices, M.; et al. Determination of an Optimal Dosing Regimen for Fexinidazole, a Novel Oral Drug for the Treatment of Human African Trypanosomiasis: First-in-Human Studies. Clin Pharmacokinet 2014, 53, 565–580.
180. Dutta, S.; Zhang, Y.; Daszkowski, D.J.; Granneman, G.R.; Verlinden, M. Single- and Multiple-Dose Pharmacokinetics of Fiduxosin under Nonfasting Conditions in Healthy Male Subjects. J Clin Pharmacol 2002, 42, 540–546.
181. Dutta, S.; Zhang, Y.; Granneman, G.R.; Verlinden, M. Effect of Food on the Pharmacokinetics of Fiduxosin in Healthy Male Subjects. Eur J Drug Metab Pharmacokinet 2002, 27, 49–52.
182. Tjandra-Maga, T.; Verbesselt, R.; van Hecken, A.; Mullie, A.; de Schepper, P. Flecainide: Single and Multiple Oral Dose Kinetics, Absolute Bioavailability and Effect of Food and Antacid in Man. Br J Clin Pharmacol 1986, 22, 309–316.
183. Michiels M.; Hendriks R.; Heykants J.; Bossche H. V. D. The Pharmacokinetics of Mebendazole and Flubendazole in Animals and Man. Arch Int Pharmacodyn Ther 1982, 256, 180–191.
184. Zimmermann, T.; Yeates, R.A.; Laufen, H.; Pfaff, G.; Wildfeuer, A. Influence of Concomitant Food Intake on the Oral Absorption of Two Triazole Antifungal Agents, Itraconazole and Fluconazole. Eur J Clin Pharmacol . 1994, 46, 147–150.
185. Orlando, R.; de Martin, S.; Andrighetto, L.; Floreani, M.; Palatini, P. Fluvoxamine Pharmacokinetics in Healthy Elderly Subjects and Elderly Patients with Chronic Heart Failure. Br J Clin Pharmacol 2010, 69, 279–286.
186. van Harten, J.; van Bemmel, P.; Dobrinska, M.R.; Ferguson, R.K.; Raghoebar, M. Bioavailability of Fluvoxamine given with and without Food. Biopharm Drug Dispos 1991, 12, 571–576.
187. Fosamprenavir Monograph Available online: https://pdf.hres.ca/dpd_pm/00037886.PDF (accessed on 29 May 2021).
188. Colon-Gonzalez, F.; Kraft, W.K. Pharmacokinetic Evaluation of Fosaprepitant Dimeglumine. Expert Opin Drug Metab Toxicol 2010, 6, 1277–1286.
189. Baluom, M.; Grossbard, E.B.; Mant, T.; Lau, D.T.W. Pharmacokinetics of Fostamatinib, a Spleen Tyrosine Kinase (SYK) Inhibitor, in Healthy Human Subjects Following Single and Multiple Oral Dosing in Three Phase I Studies. Br J Clin Pharmacol 2013, 76, 78–88.
190. Flanagan, T.; Martin, P.; Gillen, M.; Mathews, D.; Lisbon, E.; Kruusmägi, M. Effects of Ranitidine (Antacid), Food, and Formulation on the Pharmacokinetics of Fostamatinib: Results from Five Phase I Clinical Studies. Eur J Clin Pharmacol 2017, 73, 185–195.
191. Larsen, M.S.; Frølund, S.; Nøhr, M.K.; Nielsen, C.U.; Garmer, M.; Kreilgaard, M.; Holm, R. In Vivo and in Vitro Evaluations of Intestinal Gabapentin Absorption: Effect of Dose and Inhibitors on Carrier-Mediated Transport. Pharm Res 2015, 32, 898–909.
192. Bockbrader, H.N.; Wesche, D.; Miller, R.; Chapel, S.; Janiczek, N.; Burger, P. A Comparison of the Pharmacokinetics and Pharmacodynamics of Pregabalin and Gabapentin. Clin Pharmacokinet 2010, 49, 661–669.
193. Elwes, R.D.C.; Binnie, C.D. Clinical Pharmacokinetics of Newer Antiepileptic Drugs: Lamotrigine, Vigabatrin, Gabapentin and Oxcarbazepine. Clin Pharmacokinet 1996, 30, 403–415.
194. Lavelle, J.; Follansbee, S.; Trapnell, C.B.; Buhles, W.C.; Griffy, K.G.; Jung, D.; Dorr, A.; Connor, J. Effect of Food on the Relative Bioavailability of Oral Ganciclovir. J Clin Pharmacol 1996, 36, 238–241.
195. Mignot, A.; Guillaume, M.; Göhler, K.; Stahlberg, H.J. Oral Bioavailability of Gatifloxacin in Healthy Volunteers under Fasting and Fed Conditions. Chemotherapy 2002, 48, 111–115.

196. Swaisland, H.C.; Smith, R.P.; Laight, A.; Kerr, D.J.; Ranson, M.; Wilder-Smith, C.H.; Duvauchelle, T. Single-Dose Clinical Pharmacokinetic Studies of Gefitinib. Clin Pharmacokinet 2005, 44, 1165–1177.
197. Vadlapatla, R.K.; Vadlapudi, A.D.; Kwatra, D.; Pal, D.; Mitra, A.K. Differential Effect of P-Gp and MRP2 on Cellular Translocation of Gemifloxacin. Int J Pharm 2011, 420, 26–33.
198. Allen, A.; Bygate, E.; Vousden, M.; Oliver, S.; Johnson, M.; Ward, C.; Cheon, A.J.; Youn Sung Choo; Kim, I.C. Multiple-Dose Pharmacokinetics and Tolerability of Gemifloxacin Administered Orally to Healthy Volunteers. Antimicrob Agents Chemother 2001, 45, 540–545.
199. Tay, L.K.; Sciacca, M.A.; Sostrin, M.B.; Farmen, R.H.; Pittman, K.A. Effect of Food on the Bioavailability of Gepirone in Humans. J Clin Pharmacol 1993, 33, 631–635.
200. Aa, L.; Fei, F.; Tan, Z.; Aa, J.; Wang, G.; Liu, C. The Pharmacokinetics Study of Ginkgolide A, B and the Effect of Food on Bioavailability after Oral Administration of Ginkgolide Extracts in Beagle Dogs. Biomed Chromatogr 2018, 32.
201. Huang, P.; Zhang, L.; Chai, C.; Qian, X.C.; Li, W.; Li, J.S.; Di, L.Q.; Cai, B.C. Effects of Food and Gender on the Pharmacokinetics of Ginkgolides A, B, C and Bilobalide in Rats after Oral Dosing with Ginkgo Terpene Lactones Extract. J Pharm Biomed Anal 2014, 100, 138–144.
202. Shaik, N.; Hee, B.; Liang, Y.; LaBadie, R.R. Absolute Oral Bioavailability of Glasdegib (PF-04449913), a Smoothened Inhibitor, in Randomized Healthy Volunteers. lin Pharmacol Drug Dev 2019, 8, 895–902.
203. Giri, N.; Lam, L.H.; LaBadie, R.R.; Krzyzaniak, J.F.; Jiang, H.; Hee, B.; Liang, Y.; Shaik, M.N. Evaluation of the Effect of New Formulation, Food, or a Proton Pump Inhibitor on the Relative Bioavailability of the Smoothened Inhibitor Glasdegib (PF-04449913) in Healthy Volunteers. Cancer Chemother Pharmacol 2017, 80, 1249–1260.
204. Marathe P. H.; Arnold M. E.; Meeker J.; Greene D. S.; Barbhaiya R. H. Pharmacokinetics and Bioavailability of a Metformin/Glyburide Tablet Administered Alone and with Food. J Clin Pharmacol 2000, 40, 1494–1502.
205. Lin, C.; Lim, J.; Digiore, C.; Gural, R.; Symchowicz, B. Comparative Bioavailability of a Microsize and Ultramicrosize Griseofulvin Formulation in Man. J Int Med Res 1982, 10, 274–277.
206. Ogunbona, F.A.; Smith, I.F.; Olawoye, O.S. Fat Contents of Meals and Bioavailability of Griseofulvin in Man. J Pharm Pharmacol 1985, 37, 283–284.
207. Cass, L.M.; Moore, K.H.P.; Dallow, N.S.; Jones, A.E.; Sisson, J.R.; Prince, W.T. The Bioavailability of the Novel Nonnucleoside Reverse Transcriptase Inhibitor GW420867X Is Unaffected by Food in Healthy Male Volunteers. J Clin Pharmacol 2001, 41, 528–535.
208. Karbwang, J.; Bangchang, K.N. Clinical Pharmacokinetics of Halofantrine. Clin Pharmacokinet 1994, 27, 104–119.
209. Milton, K.; Edwards, G.; Ward, S.; Orme, M.; Breckenridge, A. Pharmacokinetics of Halofantrine in Man: Effects of Food and Dose Size. Br J Clin Pharmacol 1989, 28, 71–77.
210. Ludden, T.M.; Shepherd, A.M.M.; McNay, J.L.; Lin, M.S. Hydralazine Kinetics in Hypertensive Patients after Intravenous Administration. Clin Pharmacol Ther 1980, 28, 736–742.
211. Melander, A.; Danielson, K.; Hanson, A.; Rudell, B.; Schersten, B.; Thulin, T.; Wahlin, E. Enhancement of Hydralazine Bioavailability by Food. Clin Pharmacol Ther 1977, 22, 104–107.
212. Melhem, M.R.; Rubino, C.M.; Farr, S.J.; Robinson, C.Y. Population Pharmacokinetic Analysis for Hydrocodone Following the Administration of Hydrocodone Bitartrate Extended-Release Capsules. Clin Pharmacokinet 2013, 52, 907–917.
213. Pham, P.C.; Khaing, K.; Sievers, T.M.; Pham, P.M.; Miller, J.M.; Pham, S. v.; Pham, P.A.; Pham, P.T. 2017 Update on Pain Management in Patients with Chronic Kidney Disease. Clin Kidney J 2017, 10, 688–697.
214. Bond, M.; Rabinovich-Guilatt, L.; Selim, S.; Darwish, M.; Tracewell, W.; Robertson, P.; Yang, R.; Malamut, R.; Colucci, P.; Ducharme, M.P.; et al. Effect of Food on the Pharmacokinetics of Single- and Multiple-Dose Hydrocodone Extended Release in Healthy Subjects. Clin Drug Investig 2017, 37, 1153–1163.
215. Scheers, E.; Leclercq, L.; de Jong, J.; Bode, N.; Bockx, M.; Laenen, A.; Cuyckens, F.; Skee, D.; Murphy, J.; Sukbuntherng, J.; et al. Absorption, Metabolism, and Excretion of Oral 14C Radiolabeled Ibrutinib: An Open-Label, Phase I, Single-Dose Study in Healthy Men. Drug Metab Dispos 2015, 43, 289–297.
216. de Jong, J.; Sukbuntherng, J.; Skee, D.; Murphy, J.; O'Brien, S.; Byrd, J.C.; James, D.; Hellemans, P.; Loury, D.J.; Jiao, J.; et al. The Effect of Food on the Pharmacokinetics of Oral Ibrutinib in Healthy Participants and Patients with Chronic Lymphocytic Leukemia. Cancer Chemother Pharmacol 2015, 75, 907–916.
217. Chen, J.; Liu, D.; Zheng, X.; Zhao, Q.; Jiang, J.; Hu, P. Relative Contributions of the Major Human CYP450 to the Metabolism of Icotinib and Its Implication in Prediction of Drug-Drug Interaction between Icotinib and CYP3A4 Inhibitors/Inducers Using Physiologically Based Pharmacokinetic Modeling. Expert Opin Drug Metab Toxicol 2015, 11, 857–868.
218. Liu, D.; Jiang, J.; Zhang, L.; Tan, F.; Wang, Y.; Zhang, D.; Hu, P. Clinical Pharmacokinetics of Icotinib, an Anti-Cancer Drug: Evaluation of Dose Proportionality, Food Effect, and Tolerability in Healthy Subjects. Cancer Chemother Pharmacol 2014, 73, 721–727.
219. Ciraulo, D.A.; Barnhill, J.G.; Jaffe, J.H. Clinical Pharmacokinetics of Imipramine and Desipramine in Alcoholics and Normal Volunteers. Clin Pharmacol Ther 1988, 43, 509–518.
220. Abernethyl, D.R.; Divoll, M.; Greenblatt, D.J.; Harmatz, J.S.; Shader, R.I. Absolute Bioavailability of Imipramine: Influence of Food. Psychopharmacology 1984, 83, 104–106.
221. Engel, A.L.; Holt, G.E.; Lu, H. The Pharmacokinetics of Toll-like Receptor Agonists and the Impact on the Immune System. Expert Rev Clin Pharmacol 2011, 4, 275–289.

222. Harrison, L.I.; Astry, C.; Kumar, S.; Yunis, C. Pharmacokinetics of 852A, an Imidazoquinoline Toll-like Receptor 7-Specific Agonist, Following Intravenous, Subcutaneous, and Oral Administrations in Humans. J Clin Pharmacol 2007, 47, 962–969.
223. Hsu, A.; Granneman, G.R.; Cao, G.; Carothers, L.; Japour, A.; El-Shourbagy, T.; Dennis, S.; Berg, J.; Erdman, K.; Leonard, J.M.; et al. Pharmacokinetic Interaction between Ritonavir and Indinavir in Healthy Volunteers. Antimicrob Agents Chemother 1998, 42, 2784–2791.
224. Yeh, K.C.; Deutsch, P.J.; Haddix, H.; Hesney, M.; Hoagland, V.; Ju, W.D.; Justice, S.J.; Osborne, B.; Sterrett, A.T.; Stone, J.A.; et al. Single-Dose Pharmacokinetics of Indinavir and the Effect of Food. Antimicrob Agents Chemother 1998, 42, 332–338.
225. Lucas, S. The Pharmacology of Indomethacin. Headache 2016, 56, 436–446.
226. Ogata, H.; Aoyagi, N.; Kaniwa, N. Effects of Food on Bioavailability of Two Indomethacin Capsules Containing Different Sizes of Particles. Chem Pharm Bull 1990, 38, 1338–1340.
227. Kim, S.H.; Lee, M.G. Pharmacokinetics of Ipriflavone, an Isoflavone Derivative, after Intravenous and Oral Administration to Rats: Hepatic and Intestinal First-Pass Effects. Life Sci 2002, 70, 1299–1315.
228. Zent, C.; Smith, P. Study of the Effect of Concomitant Food on the Bioavailability of Rifampicin, Isoniazid and Pyrazinamide. Tuber Lung Dis 1995, 76, 109–113.
229. Morrison, R. Determination of the Partitioning, Stability, and Metabolite Formation of Isosorbide Dinitrate in Human and Rat Blood Using an Improved Gas - Liquid Chromatographic Assay. J Chromatogr 1998, 308, 153–164.
230. Kosoglou, T.; Kazierad, D.J.; Schentag, J.J.; Patrick, J.E.; Heimark, L.; Radwanski, E.; Christopher, D.; Flannery, B.E.; Affrime, M.B. Effect of Food on the Oral Bioavailability of Isosorbide-5-Mononitrate Administered as an Extended-Release Tablet. J Clin Pharmacol 1995, 35, 151–158.
231. Nulman, I.; Berkovitch, M.; Klein, J.; Pastuszak, A.; Lester, R.S.; Shear, N.; Koren, G. Steady-State Pharmacokinetics of Isotretinoin and Its 4-Oxo Metabolite: Implications for Fetal Safety. J Clin Pharmacol 1998, 38, 926–930.
232. Garcia, L.P.; Janzén, D.; Kanebratt, K.P.; Ericsson, H.; Lennernäs, H.; Lundahl, A. Physiologically Based Pharmacokinetic Model of Itraconazole and Two of Its Metabolites to Improve the Predictions and the Mechanistic Understanding of CYP3A4 Drug-Drug Interactions. Drug Metab Dispos 2018, 46, 1420–1433.
233. Zimmermann, T.; Yeates, R.A.; Laufen, H.; Pfaff, G.; Wildfeuer, A. Influence of Concomitant Food Intake on the Oral Absorption of Two Triazole Antifungal Agents, Itraconazole and Fluconazole. Eur J Clin Pharmacol 1994, 46, 147–150.
234. Duthaler, U.; Suenderhauf, C.; Karlsson, M.O.; Hussner, J.; Meyer zu Schwabedissen, H.; Krähenbühl, S.; Hammann, F. Population Pharmacokinetics of Oral Ivermectin in Venous Plasma and Dried Blood Spots in Healthy Volunteers. Br J Clin Pharmacol 2019, 85, 626–633.
235. Guzzo, C.A.; Furtek, C.I.; Porras, A.G.; Chen, C.; Tipping, R.; Clineschmidt, C.M.; Sciberras, D.G.; Hsieh, J.Y.K.; Lasseter, K.C. Safety, Tolerability, and Pharmacokinetics of Escalating High Doses of Ivermectin in Healthy Adult Subjects. J Clin Pharmacol 2002, 42, 1122–1133.
236. Gupta, N.; Hanley, M.J.; Xia, C.; Labotka, R.; Harvey, R.D.; Venkatakrishnan, K. Clinical Pharmacology of Ixazomib: The First Oral Proteasome Inhibitor. Clin Pharmacokinet 2019, 58, 431–449.
237. Gupta, N.; Hanley, M.J.; Venkatakrishnan, K.; Perez, R.; Norris, R.E.; Nemunaitis, J.; Yang, H.; Qian, M.G.; Falchook, G.; Labotka, R.; et al. Pharmacokinetics of Ixazomib, an Oral Proteasome Inhibitor, in Solid Tumour Patients with Moderate or Severe Hepatic Impairment. Br J Clin Pharmacol 2016, 82, 728–738.
238. Gupta, N.; Hanley, M.J.; Venkatakrishnan, K.; Wang, B.; Sharma, S.; Bessudo, A.; Hui, A.M.; Nemunaitis, J. The Effect of a High-Fat Meal on the Pharmacokinetics of Ixazomib, an Oral Proteasome Inhibitor, in Patients With Advanced Solid Tumors or Lymphoma. Journal of Clinical Pharmacology 2016, 56, 1288–1295.
239. Mannisto, P.T.; Mantyla, R.; Nykanen, S.; Lamminsivu, U.; Ottoila, P. Impairing Effect of Food on Ketoconazole Absorption. Antimicrob Agents Chemother 1982, 21, 730–733.
240. Daneshmend, T.; Roberts, C. The Influence of Food on the Oral and Intravenous Pharmacokinetics of a High Clearance Drug: A Study with Labetalol. Br J Clin Pharmacol 1982, 14, 73–78.
241. Cawello, W. Clinical Pharmacokinetic and Pharmacodynamic Profile of Lacosamide. Clin Pharmacokinet 2015, 54, 901–914.
242. Naderer, O.; Jones, L.S.; Zhu, J.; Coffin, M.D.; Kurtinecz, M.; Dumont, E. The Effect of Food and Formulation on the Pharmacokinetics, Safety, and Tolerability of GSK1322322 in Healthy Volunteers. Clin Pharmacol Drug Dev 2015, 4, 49–55.
243. Burris, H.A.; Taylor, C.W.; Jones, S.F.; Koch, K.M.; Versola, M.J.; Arya, N.; Fleming, R.A.; Smith, D.A.; Pandite, L.; Spector, N.; et al. A Phase I and Pharmacokinetic Study of Oral Lapatinib Administered Once or Twice Daily in Patients with Solid Malignancies. Clin Cancer Res 2009, 15, 6702–6708.
244. Lewis, L.D.; Koch, K.M.; Reddy, N.J.; Cohen, R.B.; Lewis, N.L.; Whitehead, B.; Mackay, K.; Stead, A.; Beelen, A.P. Effects of Food on the Relative Bioavailability of Lapatinib in Cancer Patients. J Clin Oncol 2009, 27, 1191–1196.
245. Chen, N.; Zhou, S.; Palmisano, M. Clinical Pharmacokinetics and Pharmacodynamics of Lenalidomide. Clin Pharmacokinet 2017, 56, 139–152.
246. Gupta, A.; Jarzab, B.; Capdevila, J.; Shumaker, R.; Hussein, Z. Population Pharmacokinetic Analysis of Lenvatinib in Healthy Subjects and Patients with Cancer. Br J Clin Pharmacol 2016, 81, 1124–1133.
247. Shen, Z.; Rowlings, C.; Kerr, B.; Hingorani, V.; Manhard, K.; Quart, B.; Yeh, L.T.; Storgard, C. Pharmacokinetics, Pharmacodynamics, and Safety of Lesinurad, a Selective Uric Acid Reabsorption Inhibitor, in Healthy Adult Males. Drug Des Devel Ther 2015, 9, 3423–3434.

248. Shen, Z.; Lee, C.A.; Valdez, S.; Yang, X.; Wilson, D.M.; Flanagan, T.; Gillen, M. Effects of Food and Antacids on Pharmacokinetics and Pharmacodynamics of Lesinurad, a Selective Urate Reabsorption Inhibitor. Clin Pharmacol Drug Dev 2019, 8, 647–656.
249. Kamimura, H.; Ito, S.; Chijiwa, H.; Okuzono, T.; Ishiguro, T.; Yamamoto, Y.; Nishinoaki, S.; Ninomiya, S.I.; Mitsui, M.; Kalgutkar, A.S.; et al. Simulation of Human Plasma Concentration–Time Profiles of the Partial Glucokinase Activator PF-04937319 and Its Disproportionate N-Demethylated Metabolite Using Humanized Chimeric Mice and Semi-Physiological Pharmacokinetic Modeling. Xenobiotica 2017, 47, 382–393.
250. Niazi, M.; Skrtic, S.; Ruth, M.; Holmberg, A.A. Pharmacokinetic Profile of Lesogaberan (AZD3355) in Healthy Subjects: A Novel GABAB-Receptor Agonist Reflux Inhibitor. Drugs R D 2011, 11, 77–83.
251. Patsalos, P.N. Pharmacokinetic Profile of Levetiracetam toward Ideal Characteristics. Pharmacol Ther 2000, 85, 77–85.
252. Fish, D.N.; Chow, A.T. The Clinical Pharmacokinetics of Levofloxacin. Clin Pharmacokinet 1997, 32, 101–119.
253. Kodaira, H.; Kusuhara, H.; Fujita, T.; Ushiki, J.; Fuse, E.; Sugiyama, Y. Quantitative Evaluation of the Impact of Active Efflux by P-Glycoprotein and Breast Cancer Resistance Protein at the Blood-Brain Barrier on the Predictability of the Unbound Concentrations of Drugs in the Brain Using Cerebrospinal Fluid Concentration as a Surrogate. J Pharmacol Exp Ther 2011, 339, 935–944.
254. Lee, L.J.; Hafkin, B.; Lee, I. der; Hoh, J.; Dix, R. Effects of Food and Sucralfate on a Single Oral Dose of 500 Milligrams of Levofloxacin in Healthy Subjects. Antimicrob Agents Chemother 1997, 41, 2196–2200.
255. Stalker, D.J.; Jungbluth, G.L. Clinical Pharmacokinetics of Linezolid, a Novel Oxazolidinone Antibacterial. Clin Pharmacokinet 2003, 42, 1129–1140.
256. Welshman, I.R.; Sisson, T.A.; Jungbluth, G.L.; Stalker, D.J.; Hopkins, N.K. Linezolid Absolute Bioavailability and the Effect of Food on Oral Bioavailability. Biopharm Drug Dispos 2001, 22, 91–97.
257. Xiong, H.; Chiu, Y.L.; Ricker, J.L.; LoRusso, P. Results of a Phase 1, Randomized Study Evaluating the Effects of Food and Diurnal Variation on the Pharmacokinetics of Linifanib. Cancer Chemother Pharmacol 2014, 74, 55–61.
258. Mojaverian, P.; Rocci, M.L.; Vlasses, P.H.; Hoholick, C.; Clementi, R.A.; Ferguson, R.K. Effect of Food on the Bioavailability of Lisinopril, a Nonsulfhydryl Angiotensin-converting Enzyme Inhibitor. J Pharm Sci 1986, 75, 395–397.
259. Beermann, B. Pharmacokinetics of Lisinopril. Am J Med 1988, 85, 25–30.
260. Zhu, Y.; Statkevich, P.; Cutler, D.L. Effect of Food on the Pharmacokinetics of Lonafarnib (SCH 66336) Following Single and Multiple Doses. Int J Clin Pharmacol Ther 2007, 45, 539–547.
261. Morgillo, F.; Lee, H.Y. Lonafarnib in Cancer Therapy. Expert Opin Investig Drugs 2006, 15, 709–719.
262. van Kampen, J.J.A.; Reedijk, M.L.; Burgers, P.C.; Dekker, L.J.M.; Hartwig, N.G.; van der Ende, I.E.; de Groot, R.; Osterhaus, A.D.M.E.; Burger, D.M.; Luider, T.M.; et al. Ultra-Fast Analysis of Plasma and Intracellular Levels of HIV Protease Inhibitors in Children: A Clinical Application of MALDI Mass Spectrometry. PLoS ONE 2010, 5.
263. Klein, C.E.; Chiu, Y.L.; Awni, W.; Zhu, T.; Heuser, R.S.; Doan, T.; Breitenbach, J.; Morris, J.B.; Brun, S.C.; Hanna, G.J. The Tablet Formulation of Lopinavir/Ritonavir Provides Similar Bioavailability to the Soft-Gelatin Capsule Formulation with Less Pharmacokinetic Variability and Diminished Food Effect. J Acquir Immune Defic Syndr 2007, 44, 401–410.
264. Haria, M.; Fitton, A.; Peters, D.H. Loratadine: A Reappraisal of Its Pharmacological Properties and Therapeutic Use in Allergic Disorders. Drugs 1994, 48, 617–637.
265. Nomeir, A.A.; Mojaverian, P.; Kosoglou, T.; Affrime, M.B.; Nezamis, J.; Radwanski, E.; Lin, C.C.; Cayen, M.N. Influence of Food on the Oral Bioavailability of Loratadine and Pseudoephedrine from Extended-Release Tablets in Healthy Volunteers. J Clin Pharmacol 1996, 36, 923–930.
266. Nguyen, H.Q.; Lin, J.; Kimoto, E.; Callegari, E.; Tse, S.; Obach, R.S. Prediction of Losartan-Active Carboxylic Acid Metabolite Exposure Following Losartan Administration Using Static and Physiologically Based Pharmacokinetic Models. J Pharm Sci 2017, 106, 2758–2770.
267. Borrmann, S.; Sallas, W.M.; Machevo, S.; González, R.; Björkman, A.; Mårtensson, A.; Hamel, M.; Juma, E.; Peshu, J.; Ogutu, B.; et al. The Effect of Food Consumption on Lumefantrine Bioavailability in African Children Receiving Artemether-Lumefantrine Crushed or Dispersible Tablets (Coartem®) for Acute Uncomplicated Plasmodium Falciparum Malaria. Trop Med Int Health 2010, 15, 434–441.
268. Greenberg, W.M.; Citrome, L. Pharmacokinetics and Pharmacodynamics of Lurasidone Hydrochloride, a Second-Generation Antipsychotic: A Systematic Review of the Published Literature. Clin Pharmacokinet 2017, 56, 493–503.
269. Preskorn, S.; Ereshefsky, L.; Chiu, Y.Y.; Poola, N.; Loebel, A. Effect of Food on the Pharmacokinetics of Lurasidone: Results of Two Randomized, Open-Label, Crossover Studies. Hum Psychopharmacol 2013, 28, 495–505.
270. Rosillon, D.; Stockis, A.; Poli, G.; Acerbi, D.; Lins, R.; Jeanbaptiste, B. Food Effect on the Oral Bioavailability of Manidipine: Single Dose, Randomized, Crossover Study in Healthy Male Subjects. Eur J Drug Metab Pharmacokinet 1998, 23, 197–202.
271. Stockis, A.; Gengler, C.; Goethals, F.; Jeanbaptiste, B.; Lens, S.; Poli, G.; Acerbi, D. Single Oral Dose Pharmacokinetic Interaction Study of Manidipine and Delapril in Healthy Volunteers. Arzneimittelforschung 2003, 53, 627–634.
272. Abel, S.; Russell, D.; Whitlock, L.A.; Ridgway, C.E.; Nedderman, A.N.R.; Walker, D.K. Assessment of the Absorption, Metabolism and Absolute Bioavailability of Maraviroc in Healthy Male Subjects. Br J Clin Pharmacol 2008, 65, 60–67.
273. Wendling, T.; Ogungbenro, K.; Pigeolet, E.; Dumitras, S.; Woessner, R.; Aarons, L. Model-Based Evaluation of the Impact of Formulation and Food Intake on the Complex Oral Absorption of Mavoglurant in Healthy Subjects. Pharm Res 2015, 32, 1764–1778.

274. Münst, G.J.; Karlaganis, G.; Bircher, J. Plasma Concentrations of Mebendazole during Treatment of Echinococcosis - Preliminary Results. Eur J Clin Pharmacol 1980, 17, 375–378.
275. Dawson, M.; Braithwaite, P.; Roberts, M.; Watson, T. The Pharmacokinetics and Bioavailability of a Tracer Dose of [3H]-Mebendazole in Man. Br J Clin Pharmacol 1985, 19, 79–86.
276. Crevoisier, C.; Handschin, J.; Barré, J.; Roumenov, D.; Kleinbloesem, C. Food Increases the Bioavailability of Mefloquine. European Journal of Clinical Pharmacology 1997, 53, 135–139.
277. Liston, D.R.; Davis, M. Clinically Relevant Concentrations of Anticancer Drugs: A Guide for Nonclinical Studies. Clin Cancer Res 2017, 23, 3489–3498.
278. Deschamps, B.; Musaji, N.; Gillespie, J.A. Food Effect on the Bioavailability of Two Distinct Formulations of Megestrol Acetate Oral Suspension. Int J Nanomedicine 2009, 4, 185–192.
279. Reece, P.A.; Kotasek, D.; Morris, R.G.; Dale, B.M.; Sage, R.E. The Effect of Food on Oral Melphalan Absorption. Cancer Chemother Pharmacol 1986, 16, 194–197.
280. Uematsu, T.; Nagashima, S.; Niwa, M.; Kohno, K.I.; Sassa, T.; Ishii, M.; Tomono, Y.; Yamato, C.; Kanamaru, M. Effect of Dietary Fat Content on Oral Bioavailability of Menatetrenone in Humans. J Pharm Sci 1996, 85, 1012–1016.
281. Graham, G.G.; Punt, J.; Arora, M.; Day, R.O.; Doogue, M.P.; Duong, J.K.; Furlong, T.J.; Greenfield, J.R.; Greenup, L.C.; Kirkpatrick, C.M.; et al. Clinical Pharmacokinetics of Metformin. Clin Pharmacokinet 2011, 50, 81–98.
282. Marathe P. H.; Arnold M. E.; Meeker J.; Greene D. S.; Barbhaiya R. H. Pharmacokinetics and Bioavailability of a Metformin/Glyburide Tablet Administered Alone and with Food. J Clin Pharmacol 2000, 40, 1494–1502.
283. Kozloski, G.D.; de Vito, J.M.; Kisicki, J.C.; Johnson, J.B. The Effect of Food on the Absorption of Methotrexate Sodium Tablets in Healthy Volunteers. Arthritis Rheum 1992, 35, 761–764.
284. Kimko, H.C.; Cross, J.T.; Abernethy, D.R. Pharmacokinetics and Clinical Effectiveness of Methylphenidate. Clin Pharmacokinet 1999, 37, 457–470.
285. Midha, K.K.; McKay, G.; Rawson, M.J.; Korchinski, E.D.; Hubbard, J.W. Effects of Food on the Pharmacokinetics of Methylphenidate. Pharmaceutical Research 2001, 18, 1185–1189.
286. Vergin, H.; Fisch, U.; Mahr, G.; Winterhalter, B. Analysis of Formulation and Food Effect on the Absorption of Metaclopramide. Int J Clin Pharmacol Ther 2002, 40, 169–174.
287. Bateman, D.N. Clinical Pharmacokinetics of Metoclopramide. Clin Pharmacokinet 1983, 8, 523–529.
288. Melander, A.; Danielson, K.; Scherstén, B.; Wåhlin, E. Enhancement of the Bioavailability of Propranolol and Metoprolol by Food. Clin Pharmacol Ther 1977, 22, 108–112.
289. Yoon, I.S.; Choi, M.K.; Kim, J.S.; Shim, C.K.; Chung, S.J.; Kim, D.D. Pharmacokinetics and First-Pass Elimination of Metoprolol in Rats: Contribution of Intestinal First-Pass Extraction to Low Bioavailability of Metoprolol. Xenobiotica 2011, 41, 243–251.
290. McCafferty, E.H.; Scott, L.J. Migalastat: A Review in Fabry Disease. Drugs 2019, 79, 543–554.
291. Johnson, F.K.; Mudd, P.N.; Janmohamed, S.G. Relative Bioavailability and the Effect of Meal Type and Timing on the Pharmacokinetics of Migalastat in Healthy Volunteers. Clin Pharmacol Drug Dev 2015, 4, 193–202.
292. Takusagawa, S.; Ushigome, F.; Nemoto, H.; Takahashi, Y.; Li, Q.; Kerbusch, V.; Miyashita, A.; Iwatsubo, T.; Usui, T. Intestinal Absorption Mechanism of Mirabegron, a Potent and Selective B3-Adrenoceptor Agonist: Involvement of Human Efflux and/or Influx Transport Systems. Mol Pharm 2013, 10, 1783–1794.
293. Lee, J.; Zhang, W.; Moy, S.; Kowalski, D.; Kerbusch, V.; van Gelderen, M.; Sawamoto, T.; Grunenberg, N.; Keirns, J. Effects of Food Intake on the Pharmacokinetic Properties of Mirabegron Oral Controlled-Absorption System: A Single-Dose, Randomized, Crossover Study in Healthy Adults. Clin Ther 2013, 35, 333–341.
294. Langel, K.; Gjerde, H.; Favretto, D.; Lillsunde, P.; Øiestad, E.L.; Ferrara, S.D.; Verstraete, A.G. Comparison of Drug Concentrations between Whole Blood and Oral Fluid. Drug Test Anal 2014, 6, 461–471.
295. Timmer, C.J.; Ad Sitsen, J.M.; Delbressine, L.P. Clinical Pharmacokinetics of Mirtazapine. Clin Pharmacokinet 2000, 38, 461–474.
296. Eskens F. A.; Levitt N. C.; Sparreboom A.; Choi L.; Mather R.; Verweij J.; Harris A. L. Effect of Food on the Pharmacokinetics of Oral MMI270B (CGS 27023A), a Novel Matrix Metalloproteinase Inhibitor. Clin Cancer Res 2000, 6, 431–433.
297. Zheng, J.; Xin, Y.; Zhang, J.; Subramanian, R.; Murray, B.P.; Whitney, J.A.; Warr, M.R.; Ling, J.; Moorehead, L.; Kwan, E.; et al. Pharmacokinetics and Disposition of Momelotinib Revealed a Disproportionate Human Metabolite - Resolution for Clinical Development. Drug Metab Dispos 2018, 46, 237–247.
298. Xin, Y.; Shao, L.; Maltzman, J.; Stefanidis, D.; Hemenway, J.; Tarnowski, T.; Deng, W.; Silverman, J.A. The Relative Bioavailability, Food Effect, and Drug Interaction With Omeprazole of Momelotinib Tablet Formulation in Healthy Subjects. Clin Pharmacol Drug Dev 2018, 7, 277–286.
299. Buice, R.G.; Subramanian, V.S.; Duchin, K.L.; Uko-Nne, S. Bioequivalence of a Highly Variable Drug: An Experience with Nadolol. Pharm Res 1996, 13, 1109–1115.
300. Tothfalusi, L.; Endrenyi, L.; Garcia Arieta, A. Evaluation of Bioequivalence for Highly Variable Drugs with Scaled Average Bioequivalence. Clin Pharmacokinet 2009, 48, 725–743.
301. Barbeau, G.; Belanger, P.M. Pharmacokinetics of Nalidixic Acid in Old and Young Volunteers. J Clin Pharmacol 1982, 22, 490–496.

302. Ogata, H.; Aoyagi, N.; Kaniwa, N.; Ejima, A. Effect of Food on Bioavailability of Nalidixic Acid from Uncoated Tablets Having Different Dissolution Rates. J Pharmacobiodyn 1984, 7, 760–767.
303. Bui, K.; She, F.; Hutchison, M.; Brunnström, Å.; Sostek, M. Absorption, Distribution, Metabolism, and Excretion of [14C]-Labeled Naloxegol in Healthy Subjects. Int J Clin Pharmacol Ther 2015, 53, 838–846.
304. Weaver M. L.; Orwig B. A.; Rodriguez L. C.; Graham E. D.; Chin J. A.; Shapiro M. J.; McLeod J. F.; Mangold J. B. Pharmacokinetics and Metabolism of Nateglinide in Humans. Drug Metab Dispos 2001, 29, 415–421.
305. Karara, A.H.; Dunning, B.E.; McLeod, J.F. The Effect of Food on the Oral Bioavailability and the Pharmacodynamic Actions of the Insulinotropic Agent Nateglinide in Healthy Subjects. J Clin Pharmacol 1999, 39, 172–179.
306. Regazzi, M.; Maserati, R.; Villani, P.; Cusato, M.; Zucchi, P.; Briganti, E.; Roda, R.; Sacchelli, L.; Gatti, F.; Delle Foglie, P.; et al. Clinical Pharmacokinetics of Nelfinavir and Its Metabolite M8 in Human Immunodeficiency Virus (HIV)-Positive and HIV-Hepatitis C Virus-Coinfected Subjects. Antimicrob Agents Chemother 2005, 49, 643–649.
307. Challenor, V.; Waller, D.; Renwick, A.; Gruchy, B.; George, C. The Trans-hepatic Extraction of Nifedipine. Br J Clin Pharmacol 1987, 24, 473–477.
308. Schug, B.S.; Brendel, E.; Wonnemann, M.; Wolf, D.; Wargenau, M.; Dingler, A.; Blume, H.H. Dosage Form-Related Food Interaction Observed in a Marketed Once-Daily Nifedipine Formulation after a High-Fat American Breakfast. Eur J Clin Pharmacol 2002, 49, 119–125.
309. Kang, W.; Kim, K.; Kim, E.Y.; Kwon, K. il; Bang, J.S.; Yoon, Y.R. Effect of Food on Systemic Exposure to Niflumic Acid Following Postprandial Administration of Talniflumate. Eur J Clin Pharmacol 2008, 64, 1027–1030.
310. Tian, X.; Zhang, H.; Heimbach, T.; He, H.; Buchbinder, A.; Aghoghovbia, M.; Hourcade-Potelleret, F. Clinical Pharmacokinetic and Pharmacodynamic Overview of Nilotinib, a Selective Tyrosine Kinase Inhibitor. J Clin Pharmacol 2018, 58, 1533–1540.
311. Terakawa, M.; Tokuma, Y.; Shishido, A.; Noguchi, H. Pharmacokinetics of Nilvadipine in Healthy Volunteers. J Clin Pharmacol 1987, 27, 111–117.
312. Terakawa, M.; Tokuma, Y.; Shishido, A.; Yasuda, K.; Noguchi, H. Effect of Two Different Meals on Bioavailability of Nilvadipine in Healthy Volunteers. J Clin Pharmacol 1987, 27, 293–296.
313. Bates, T.R.; Sequeira, J.A.; Tembo, A. v. Effect of Food on Nitrofurantoin Absorption. Clin Pharmacol Ther 1974, 16, 63–68.
314. Nada, A.H.; Sharaf, M.A.; el Gholmy, Z.A.; Khalafallah, N.M. Comparative Bioavailability of Norfloxacin Tablets Based on Blood and Urine Data. Med Princ Pract 2007, 16, 426–431.
315. Stein, G.E. Review of the Bioavailability and Pharmacokinetics of Oral Norfloxacin. Am J Med 1987, 82, 18–21.
316. Rodvold, K.A.; Pai, M.P. Pharmacokinetics and Pharmacodynamics of Oral and Intravenous Omadacycline. Clin Infect Dis 2019, 69, S16–S22.
317. Tzanis, E.; Manley, A.; Villano, S.; Tanaka, S.K.; Bai, S.; Loh, E. Effect of Food on the Bioavailability of Omadacycline in Healthy Participants. J Clin Pharmacol 2017, 57, 321–327.
318. Suri, A.; Pham, T.; Maclean, D.B. A Phase 1, Randomized, Single-Dose Crossover Pharmacokinetic Study to Investigate the Effect of Food Intake on Absorption of Orteronel (TAK-700) in Healthy Male Subjects. Clin Pharmacol Drug Dev 2016, 5, 188–195.
319. Koskimies, P.; Katila, K.; Lammintausta, R.; Aaltonen, A.M.; Vuorinen, J.; Saarni, O.; Scheinin, M. Oral Bioavailability of Ospemifene Improves with Food Intake. Int J Clin Pharmacol Ther 2013, 51, 787–794.
320. Davies, N.M. Clinical Pharmacokinetics of Oxaprozin. Clin Pharmacokinet 1998, 35, 425–436.
321. Chiang S. T.; Knowles J. A.; Hubsher J. A.; Ruelius H. W.; Walker B. R. Effects of Food on Oxaprozin Bioavailability. J Clin Pharmacol 1984, 24, 381–385.
322. Bach, T.; Galbiati, S.; Kennedy, J.K.; Deye, G.; Nomicos, E.Y.H.; Codd, E.E.; Garcia, H.H.; Horton, J.; Gilman, R.H.; Gonzalez, A.E.; et al. Pharmacokinetics, Safety, and Tolerability of Oxfendazole in Healthy Adults in an Open-Label Phase 1 Multiple Ascending Dose and Food Effect Study. Antimicrob Agents Chemother 2020, 64, 1018–1020.
323. An, G.; Murry, D.J.; Gajurel, K.; Bach, T.; Deye, G.; Stebounova, L. v.; Codd, E.E.; Horton, J.; Gonzalez, A.E.; Garcia, H.H.; et al. Pharmacokinetics, Safety, and Tolerability of Oxfendazole in Healthy Volunteers: A Randomized, Placebo-Controlled First-Inhuman Single-Dose Escalation Study. Antimicrob Agents Chemother 2019, 63, 2255-undefined.
324. McKellar Q. A.; Galbraith E. A.; Baxter P. Oral Absorption and Bioavailability of Fenbendazole in the Dog and the Effect of Concurrent Ingestion of Food. J Vet Pharmacol Ther 1993, 16, 189–198.
325. Olivares-Morales, A.; Ghosh, A.; Aarons, L.; Rostami-Hodjegan, A. Development of a Novel Simplified PBPK Absorption Model to Explain the Higher Relative Bioavailability of the OROS® Formulation of Oxybutynin. AAPS J 2016, 18, 1532–1549.
326. Sathyan, G.; Chancellor, M.B.; Gupta, S.K. Effect of OROS® Controlled-Release Delivery on the Pharmacokinetics and Pharmacodynamics of Oxybutynin Chloride. Br J Clin Pharmacol 2001, 52, 409–417.
327. Lukkari, E.; Castrèn-Kortekangas, P.; Juhakoski, A.; Löyttyniemi, E.; Aranko, K.; Neuvonen, P.J. Effect of Food on the Bioavailability of Oxybutynin from a Controlled Release Tablet. Eur J Clin Pharmacol 1996, 50, 221–223.
328. Yu, Y.; Loi, C.M.; Hoffman, J.; Wang, D. Physiologically Based Pharmacokinetic Modeling of Palbociclib. J Clin Pharmacol 2017, 57, 173–184.
329. Ruiz-Garcia, A.; Plotka, A.; O'Gorman, M.; Wang, D.D. Effect of Food on the Bioavailability of Palbociclib. Cancer Chemother Pharmacol 2017, 79, 527–533.

330. Savelieva, M.; Woo, M.M.; Schran, H.; Mu, S.; Nedelman, J.; Capdeville, R. Population Pharmacokinetics of Intravenous and Oral Panobinostat in Patients with Hematologic and Solid Tumors. Eur J Clin Pharmacol 2015, 71, 663–672.
331. Shapiro, G.I.; Frank, R.; Dandamudi, U.B.; Hengelage, T.; Zhao, L.; Gazi, L.; Porro, M.G.; Woo, M.M.; Lewis, L.D. The Effect of Food on the Bioavailability of Panobinostat, an Orally Active Pan-Histone Deacetylase Inhibitor, in Patients with Advanced Cancer. Cancer Chemother Pharmacol 2012, 69, 555–562.
332. Finkel, Y.; Bolme, P.; Eriksson, M. The Effect of Food on the Oral Absorption of Penicillin V Preparations in Children. Acta Pharmacol Toxicol 1981, 49, 301–304.
333. Wollenberg, L.A.; Corson, D.T.; Nugent, C.A.; Peterson, F.L.; Ptaszynski, A.M.; Arrigo, A.; Mannila, C.G.; Litwiler, K.S.; Bell, S.J. An Exploratory, Randomized, Parallel-Group, Open-Label, Relative Bioavailability Study with an Additional Two-Period Crossover Food-Effect Study Exploring the Pharmacokinetics of Two Novel Formulations of Pexmetinib (ARRY-614). Cin Pharmacol 2015, 7, 87–95.
334. Giri, N.; Lam, L.H.; LaBadie, R.R.; Krzyzaniak, J.F.; Jiang, H.; Hee, B.; Liang, Y.; Shaik, M.N. Evaluation of the Effect of New Formulation, Food, or a Proton Pump Inhibitor on the Relative Bioavailability of the Smoothened Inhibitor Glasdegib (PF-04449913) in Healthy Volunteers. Cancer Chemother Pharmacol 2017, 80, 1249–1260.
335. Kong, S.T.; Lim, S.H.; Lee, W.B.; Kumar, P.K.; Wang, H.Y.S.; Ng, Y.L.S.; Wong, P.S.; Ho, P.C. Clinical Validation and Implications of Dried Blood Spot Sampling of Carbamazepine, Valproic Acid and Phenytoin in Patients with Epilepsy. PLoS ONE 2014, 9.
336. Cranford, R.E.; Leppik, I.E.; Patrick, B.; Anderson, C.B.; Kostick, B. Intravenous Phenytoin: Clinical and Pharmacokinetic Aspects. Neurology 1978, 28, 874–880.
337. Lu T.; Fraczkiewicz G.; Salphati L.; Budha N.; Dalziel G.; Smelick G. S.; Morrissey K. M.; Davis J. D.; Jin J. Y.; Ware J. A. Combining “Bottom-up” and “Top-down” Approaches to Assess the Impact of Food and Gastric PH on Pictilisib (GDC-0941) Pharmacokinetics. CPT Pharmacometrics Syst Pharmacol 2017, 6, 747–755.
338. Reuter, S.E.; Evans, A.M.; Shakib, S.; Lungershausen, Y.; Francis, B.; Valentini, G.; Bacchieri, A.; Ubben, D.; Pace, S. Effect of Food on the Pharmacokinetics of Piperaquine and Dihydroartemisinin. Clin Drug Investig 2015, 35, 559–567.
339. Schachter, M. Chemical, Pharmacokinetic and Pharmacodynamic Properties of Statins: An Update. Fundam Clin Pharmacol 2005, 19, 117–125.
340. Cherkaoui-Rbati, M.H.; Paine, S.W.; Littlewood, P.; Rauch, C. A Quantitative Systems Pharmacology Approach, Incorporating a Novel Liver Model, for Predicting Pharmacokinetic Drug-Drug Interactions. PLoS ONE 2017, 12.
341. Abdel-Rahman, S.M.; Kearns, G.L. Single-Dose Pharmacokinetics of a Pleconaril (VP63843) Oral Solution and Effect of Food. Antimicrob Agents Chemother 1998, 42, 2706–2709.
342. Narasimhan, N.I.; Dorer, D.J.; Niland, K.; Haluska, F.; Sonnichsen, D. Effects of Ketoconazole on the Pharmacokinetics of Ponatinib in Healthy Subjects. J Clin Pharmacol 2013, 53, 974–981.
343. Narasimhan, N.I.; Dorer, D.J.; Niland, K.; Haluska, F.; Sonnichsen, D. Effects of Food on the Pharmacokinetics of Ponatinib in Healthy Subjects. J Clin Pharm Ther 2013, 38, 440–444.
344. Wiederhold, N.P. Pharmacokinetics and Safety of Posaconazole Delayed-Release Tablets for Invasive Fungal Infections. Clin Pharmacol 2015, 8, 1–8.
345. Li, Y.; Theuretzbacher, U.; Clancy, C.J.; Nguyen, M.H.; Derendorf, H. Pharmacokinetic/Pharmacodynamic Profile of Posaconazole. Clin Pharmacokinet 2010, 49, 379–396.
346. Courtney, R.; Wexler, D.; Radwanski, E.; Lim, J.; Laughlin, M. Effect of Food on the Relative Bioavailability of Two Oral Formulations of Posaconazole in Healthy Adults. Br J Clin Pharmacol 2004, 57, 218–222.
347. Meyers, C.D.; Amer, A.; Majumdar, T.; Chen, J. Pharmacokinetics, Pharmacodynamics, Safety, and Tolerability of Pradigastat, a Novel Diacylglycerol Acyltransferase 1 Inhibitor in Overweight or Obese, but Otherwise Healthy Human Subjects. J Clin Pharmacol 2015, 55, 1031–1041.
348. Ayalasomayajula, S.P.; Meyers, C.D.; Yu, J.; Kagan, M.; Matott, R.; Pal, P.; Majumdar, T.; Su, Z.; Crissey, A.; Rebello, S.; et al. Evaluation of Food Effect on the Oral Bioavailability of Pradigastat, a Diacylglycerol Acyltransferase 1 Inhibitor. Biopharm Drug Dispos 2015, 36, 452–461.
349. Cutler, D.L.; Tendolkar, A.; Grachev, I.D. Safety, Tolerability and Pharmacokinetics after Single and Multiple Doses of Preladenant (SCH420814) Administered in Healthy Subjects. J Clin Pharm Ther 2012, 37, 578–587.
350. Cutler, D.L.; Tendolkar, A.; Grachev, I.D. Evaluation of the Effects of a High-Fat Meal on the Oral Bioavailability of a Single Dose of Preladenant in Healthy Subjects. J Clin Pharmacol 2012, 52, 1698–1703.
351. Winter, H.; Ginsberg, A.; Egizi, E.; Erondu, N.; Whitney, K.; Pauli, E.; Everitt, D. Effect of a High-Calorie, High-Fat Meal on the Bioavailability and Pharmacokinetics of PA-824 in Healthy Adult Subjects. Antimicrob Agents Chemother 2013, 57, 5516–5520.
352. Nair, A.; Abrahamsson, B.; Barends, D.M.; Groot, D.W.; Kopp, S.; Polli, J.E.; Shah, V.P.; Dressman, J.B. Biowaiver Monographs for Immediate-Release Solid Oral Dosage Forms: Primaquine Phosphate. J Pharm Sci 2012, 101, 936–945.
353. Mihaly, G.; Ward, S.; Edwards, G.; Nicholl, D.; Orme, M.; Breckenridge, A. Pharmacokinetics of Primaquine in Man. I. Studies of the Absolute Bioavailability and Effects of Dose Size. Br J Clin Pharmacol 1985, 19, 745–750.
354. Cuong, B.T.; Binh, V.Q.; Dai, B.; Duy, D.N.; Lovell, C.M.; Rieckmann, K.H.; Edstein, M.D. Does Gender, Food or Grapefruit Juice Alter the Pharmacokinetics of Primaquine in Healthy Subjects? Br J Clin Pharmacol 2006, 61, 682–689.

355. Rocci, M.L.; Mojaverian, P.; Davis, R.J.; Ferguson, R.K.; Vlasses, P.H. Food-Induced Gastric Retention and Absorption of Sustained-Release Procainamide. Clin Pharm Ther 1987, 42, 45–49.
356. Siddoway, L.A.; Roden, D.M.; Woosley, R.L. Clinical Pharmacology of Propafenone: Pharmacokinetics, Metabolism and Concentration-Response Relations. Am J Cardiol 1984, 54, 9D-12D.
357. Axelson, J.; Chan, G.; Kirsten, E.; Mason, W.; Lanman, R.; Kerr, C. Food Increases the Bioavailability of Propafenone. Br J Clin Pharmacol 1987, 23, 735–741.
358. Launiainen, T.; Ojanperä, I. Drug Concentrations in Post-Mortem Femoral Blood Compared with Therapeutic Concentrations in Plasma. Drug Test Anal 2014, 6, 308–316.
359. Chao, S.T.; Prather, D.; Pinson, D.; Coen, P.; Pruitt, B.; Knowles, M.; Place, V. Effect of Food on Bioavailability of Pseudoephedrine and Brompheniramine Administered from a Gastrointestinal Therapeutic System. J Pharm Sci 1991, 80, 432–435.
360. Schmitt, C.; Charoin-Pannier, A.; McIntyre, C.; Zandt, H.; Ciorciaro, C.; Zweigler, L.; Winters, K.; Pepper, T. Effect of Food on the Pharmacokinetics and Pharmacodynamics of R1663, an Oral Factor Xa Inhibitor, in Healthy Male Volunteers. Int J Clin Pharmacol Ther 2012, 50, 566–572.
361. Brainard, D.M.; Friedman, E.J.; Jin, B.; Breidinger, S.A.; Tillan, M.D.; Wenning, L.A.; Stone, J.A.; Chodakewitz, J.A.; Wagner, J.A.; Iwamoto, M. Effect of Low-, Moderate-, and High-Fat Meals on Raltegravir Pharmacokinetics. J Clin Pharmacol 2011, 51, 422–427.
362. Laufer, R.; Paz, O.G.; di Marco, A.; Bonelli, F.; Monteagudo, E.; Summa, V.; Rowley, M. Quantitative Prediction of Human Clearance Guiding the Development of Raltegravir (MK-0518, Isentress) and Related HIV Integrase Inhibitors. Drug Metab Dispos 2009, 37, 873–883.
363. Watt K.; Manzoni P.; Cohen-Wolkowiez M.; Rizzollo S.; Boano E.; Jacqz-Aigrain E.; Benjamin D. K. Triazole Use in the Nursery: Fluconazole, Voriconazole, Posaconazole, and Ravuconazole. Curr Drug Metab 2013, 14, 193–202.
364. Beermann, D.; Schaefer, H.G.; Wargenau, M.; Heibel, B.; Sturm, Y.; Kuhlmann, J. Pharmacokinetics of the Active Metabolite of the Prodrug Repirinast in Healthy Caucasian Volunteers after a Single Oral Dose. Eur J Clin Pharmacol 1992, 42, 307–312.
365. Preston, S.L.; Drusano, G.L.; Glue, P.; Nash, J.; Gupta, S.K.; McNamara, P. Pharmacokinetics and Absolute Bioavailability of Ribavirin in Healthy Volunteers as Determined by Stable-Isotope Methodology. Antimicrob Agents Chemother 1999, 43, 2451–2456.
366. Zempleni, J.; Galloway, J.R.; McCormick, D.B. Pharmacokinetics of Orally and Intravenously Administered Riboflavin in Healthy Humans. Am J Clin Nutr 1996, 63, 54–66.
367. Subramanian, V.S.; Ghosal, A.; Kapadia, R.; Nabokina, S.M.; Said, H.M. Molecular Mechanisms Mediating the Adaptive Regulation of Intestinal Riboflavin Uptake Process. PLoS ONE 2015, 10.
368. Skinner, M.H.; Hsieh, M.; Torseth, J.; Pauloin, D.; Bhatia, G.; Harkonen, S.; Merigan, T.C.; Blaschke, T.F. Pharmacokinetics of Rifabutin. Antimicrob Agents Chemother 1989, 33, 1237–1241.
369. Chen, Y.X.; Cabana, B.; Kivel, N.; Michaelis, A. Effect of Food on the Pharmacokinetics of Rifalazil, a Novel Antibacterial, in Healthy Male Volunteers. J Clin Pharmacol 2007, 47, 841–849.
370. Loos, U.; Musch, E.; Jensen, J.C.; Mikus, G.; Schwabe, H.K.; Eichelbaum, M. Pharmacokinetics of Oral and Intravenous Rifampicin during Chronic Administration. Klin Wochenschr 1985, 63, 1205–1211.
371. Zent, C.; Smith, P. Study of the Effect of Concomitant Food on the Bioavailability of Rifampicin, Isoniazid and Pyrazinamide. Tuber Lung Dis 1995, 76, 109–113.
372. Aouri, M.; Barcelo, C.; Guidi, M.; Rotger, M.; Cavassini, M.; Hizrel, C.; Buclin, T.; Decosterd, L.A.; Csajk, C. Population Pharmacokinetics and Pharmacogenetics Analysis of Rilpivirine in HIV-1-Infected Individuals. Antimicrob Agents Chemother 2017, 61.
373. Crauwels, H.M.; van Heeswijk, R.P.G.; Buelens, A.; Stevens, M.; Boven, K.; Hoetelmans, R.M.W. Impact of Food and Different Meal Types on the Pharmacokinetics of Rilpivirine. J Clin Pharmacol 2013, 53, 834–840.
374. Frey, R.; Becker, C.; Saleh, S.; Unger, S.; van der Mey, D.; Mück, W. Clinical Pharmacokinetic and Pharmacodynamic Profile of Riociguat. Clin Pharmacokinet 2018, 57, 647–661.
375. Arora, S.; Pansari, A.; Kilford, P.; Jamei, M.; Gardner, I.; Turner, D.B. Biopharmaceutic in Vitro in Vivo Extrapolation (IVIVE) Informed Physiologically-Based Pharmacokinetic Model of Ritonavir Norvir Tablet Absorption in Humans under Fasted and Fed State Conditions. Mol Pharm 2020, 17, 2329–2344.
376. van Kampen, J.J.A.; Reedijk, M.L.; Burgers, P.C.; Dekker, L.J.M.; Hartwig, N.G.; van der Ende, I.E.; de Groot, R.; Osterhaus, A.D.M.E.; Burger, D.M.; Luider, T.M.; et al. Ultra-Fast Analysis of Plasma and Intracellular Levels of HIV Protease Inhibitors in Children: A Clinical Application of MALDI Mass Spectrometry. PLoS ONE 2010, 5.
377. Hsu, A.; Granneman, G.R.; Witt, G.; Locke, C.; Denissen, J.; Molla, A.; Valdes, J.; Smith, J.; Erdman, K.; Lyons, N.; et al. Multiple-Dose Pharmacokinetics of Ritonavir in Human Immunodeficiency Virus-Infected Subjects. Antimicrob Agents Chemother 1997, 41, 898–905.
378. Stampfuss, J.; Kubitza, D.; Becka, M.; Mueck, W. The Effect of Food on the Absorption and Pharmacokinetics of Rivaroxaban. Int J Clin Pharmacol Ther 2013, 51, 549–561.
379. Mueck, W.; Stampfuss, J.; Kubitza, D.; Becka, M. Clinical Pharmacokinetic and Pharmacodynamic Profile of Rivaroxaban. Clin Pharmacokinet 2014, 53, 1–16.

380. McLean, C.C.; Teft, W.A.; Morse, B.L.; Gryn, S.E.; Hegele, R.A.; Kim, R.B. Food Effect on Rosuvastatin Disposition and Low-Density Lipoprotein Cholesterol. Clin Pharmacol Ther 2018, 104, 525–533.
381. Perucca, E.; Cloyd, J.; Critchley, D.; Fuseau, E. Rufinamide: Clinical Pharmacokinetics and Concentration-Response Relationships in Patients with Epilepsy. Epilepsia 2008, 49, 1123–1141.
382. Cardot J. M.; Lecaillon J. B.; Czendlik C.; Godbillon J. The Influence of Food on the Disposition of the Antiepileptic Rufinamide in Healthy Volunteers. Biopharm Drug Dispos 1998, 19, 259–262.
383. Solans, A.; Carbó, M.; Peña, J.; Nadal, T.; Izquierdo, I.; Merlos, M. Influence of Food on the Oral Bioavailability of Rupatadine Tablets in Healthy Volunteers: A Single-Dose, Randomized, Open-Label, Two-Way Crossover Study. Clin Ther 2007, 29, 900–908.
384. Akahori, M.; Ayalasomayajula, S.; Langenickel, T.; Pal, P.; Zhou, W.; Sunkara, G. Pharmacokinetics After Single Ascending Dose, Food Effect, and Safety of Sacubitril/Valsartan (LCZ696), an Angiotensin Receptor and Neprilysin Inhibitor, in Healthy Japanese Subjects. Eur J Drug Metab Pharmacokinet 2017, 42, 407–416.
385. Ayalasomayajula, S.; Langenickel, T.; Pal, P.; Boggarapu, S.; Sunkara, G. Clinical Pharmacokinetics of Sacubitril/Valsartan (LCZ696): A Novel Angiotensin Receptor-Neprilysin Inhibitor. Clin Pharmacokinet 2017, 56, 1461–1478.
386. Seithel-Keuth, A.; Johne, A.; Freisleben, A.; Kupas, K.; Lissy, M.; Krösser, S. Absolute Bioavailability and Effect of Food on the Disposition of Safinamide Immediate Release Tablets in Healthy Adult Subjects. Clin Pharmacol Drug Dev 2013, 2, 79–89.
387. Musson, D.G.; Kramer, W.G.; Foehr, E.D.; Bieberdorf, F.A.; Hornfeldt, C.S.; Kim, S.S.; Dorenbaum, A. Relative Bioavailability of Sapropterin from Intact and Dissolved Sapropterin Dihydrochloride Tablets and the Effects of Food: A Randomized, Open-Label, Crossover Study in Healthy Adults. Clin Ther 2010, 32, 338–346.
388. FDA Label Available online: https://www.accessdata.fda.gov/drugsatfda_docs/label/2010/020628s032,021785s009lbl.pdf (accessed on 30 May 2021).
389. Jani, R.H.; Kansagra, K.; Jain, M.R.; Patel, H. Pharmacokinetics, Safety, and Tolerability of Saroglitazar (ZYH1), a Predominantly PPARα Agonist with Moderate PPARγ Agonist Activity in Healthy Human Subjects. Clin Drug Investig 2013, 33, 809–816.
390. Patel, M.R.; Kansagra, K.A.; Parikh, D.P.; Parmar, D. v.; Patel, H.B.; Soni, M.M.; Patil, U.S.; Patel, H. v.; Patel, J.A.; Gujarathi, S.S.; et al. Effect of Food on the Pharmacokinetics of Saroglitazar Magnesium, a Novel Dual PPAR$\alpha\gamma$ Agonist, in Healthy Adult Subjects. Clin Drug Investig 2018, 38, 57–65.
391. Mahmood, I. Clinical Pharmacokinetics and Pharmacodynamics of Selegiline: An Update. Clin Pharmacokinet 1997, 33, 91–102.
392. Barrett, J.S.; Rohatagi, S.; DeWitt, K.E.; Morales, R.J.; DiSanto, A.R. The Effect of Dosing Regimen and Food on the Bioavailability of the Extensively Metabolized, Highly Variable Drug Eldepryl® (Selegiline Hydrochloride). Am J Ther 1996, 3, 298–313.
393. Kaufmann, P.; Okubo, K.; Bruderer, S.; Mant, T.; Yamada, T.; Dingemanse, J.; Mukai, H. Pharmacokinetics and Tolerability of the Novel Oral Prostacyclin IP Receptor Agonist Selexipag. Am J Cardiovasc Drugs 2015, 15, 195–203.
394. Tomkinson, H.; McBride, E.; Martin, P.; Lisbon, E.; Dymond, A.W.; Cantarini, M.; So, K.; Holt, D. Comparison of the Pharmacokinetics of the Phase II and Phase III Capsule Formulations of Selumetinib and the Effects of Food on Exposure: Results From Two Randomized Crossover Trials in Healthy Male Subjects. Clin Ther 2017, 39, 2260-2275.e1.
395. Dymond, A.W.; Howes, C.; Pattison, C.; So, K.; Mariani, G.; Savage, M.; Mair, S.; Ford, G.; Martin, P. Metabolism, Excretion, and Pharmacokinetics of Selumetinib, an MEK1/2 Inhibitor, in Healthy Adult Male Subjects. Clin Ther 2016, 38, 2447–2458.
396. Wong S. L.; Linnen P.; Mack R.; Granneman G. R. Effects of Food, Antacid, and Dosage Form on the Pharmacokinetics and Relative Bioavailability of Sertindole in Healthy Volunteers. Biopharm Drug Dispos 1997, 18, 533–541.
397. Wong, S.L.; Cao, G.; Mack, R.J.; Granneman, G.R. Pharmacokinetics of Sertindole in Healthy Young and Elderly Male and Female Subjects. Clin Pharmacol Ther 1997, 62, 157–164.
398. Nichols, D.J.; Muirhead, G.J.; Harness, J.A. Pharmacokinetics of Sildenafil Citrate after Single Oral Doses in Healthy Male Subjects: Absolute Bioavailability, Food Effects and Dose Proportionality. Br J Clin Pharmacol 2002, 53.
399. German, P.; Mathias, A.; Brainard, D.; Kearney, B.P. Clinical Pharmacokinetics and Pharmacodynamics of Ledipasvir/Sofosbuvir, a Fixed-Dose Combination Tablet for the Treatment of Hepatitis C. Clin Pharmacokinet 2016, 55, 1337–1351.
400. MacLauchlin, C.; Schneider, S.E.; Keedy, K.; Fernandes, P.; Jamieson, B.D. Metabolism, Excretion, and Mass Balance of Solithromycin in Humans. Antimicrob Agents Chemother 2018, 62.
401. Still, J.G.; Schranz, J.; Degenhardt, T.P.; Scott, D.; Fernandes, P.; Gutierrez, M.J.; Clark, K. Pharmacokinetics of Solithromycin (CEM-101) after Single or Multiple Oral Doses and Effects of Food on Single-Dose Bioavailability in Healthy Adult Subjects. Antimicrob Agents Chemother 2011, 55, 1997–2003.
402. Kahela, P.; Anttila, M.; Tikkanen, R.; Sundquist, H. Effect of Food, Food Constituents and Fluid Volume on the Bioavailability of Sotalol. Acta Pharmacol Toxicol 1979, 44, 7–12.
403. Hoffmann, E.; Wald, J.; Lavu, S.; Roberts, J.; Beaumont, C.; Haddad, J.; Elliott, P.; Westphal, C.; Jacobson, E. Pharmacokinetics and Tolerability of SRT2104, a First-in-Class Small Molecule Activator of SIRT1, after Single and Repeated Oral Administration in Man. Br J Clin Pharmacol 2013, 75, 186–196.

404. Shinkuma D.; Hamaguchi T.; Kobayashi M.; Yamanaka Y.; Mizuno N. Effects of Food Intake on the Bioavailability of Sulpiride from AEA Film-Coated Tablet Having a PH-Dependent Dissolution Characteristic in Normal or Drug-Induced Achlorhydric Subjects. Int J Clin Pharmacol Ther Toxicol 1991, 29, 303–309.
405. Bello, C.L.; Sherman, L.; Zhou, J.; Verkh, L.; Smeraglia, J.; Mount, J.; Klamerus, K.J. Effect of Food on the Pharmacokinetics of Sunitinib Malate (SU11248), a Multi-Targeted Receptor Tyrosine Kinase Inhibitor: Results from a Phase I Study in Healthy Subjects. Anti-Cancer Drugs 2006, 17, 353–358.
406. Speed, B.; Bu, H.Z.; Pool, W.F.; Peng, G.W.; Wu, E.Y.; Patyna, S.; Bello, C.; Kang, P. Pharmacokinetics, Distribution, and Metabolism of [14C]Sunitinib in Rats, Monkeys, and Humans. Drug Metab Dispos 2012, 40, 539–555.
407. Möller A.; Iwasaki K.; Kawamura A.; Teramura Y.; Shiraga T.; Hata T.; Schäfer A.; Undre N. A. The Disposition of 14C-Labeled Tacrolimus after Intravenous and Oral Administration in Healthy Human Subjects. Drug Metab Dispos 1999, 27, 633–636.
408. Bekersky, I.; Dressler, D.; Mekki, Q.A. Effect of Low- and High-Fat Meals on Tacrolimus Absorption Following 5 Mg Single Oral Doses to Healthy Human Subjects. J Clin Pharmacol 2001, 41, 176–182.
409. FDA Label Available online: https://www.accessdata.fda.gov/drugsatfda_docs/label/2018/211651s000lbl.pdf (accessed on 30 May 2021).
410. Flanagan, S.D.; Bien, P.A.; Muñoz, K.A.; Minassian, S.L.; Prokocimer, P.G. Pharmacokinetics of Tedizolid Following Oral Administration: Single and Multiple Dose, Effect of Food, and Comparison of Two Solid Forms of the Prodrug. Pharmacotherapy 2014, 34, 240–250.
411. Park, A. young J.; Wang, J.; Jayne, J.; Fukushima, L.; Rao, A.P.; D'Argenio, D.Z.; Beringer, P.M. Pharmacokinetics of Tedizolid in Plasma and Sputum of Adults with Cystic Fibrosis. Antimicrob Agents Chemother 2018, 62.
412. Yamada, I.; Suzuki, F.; Kamiya, N.; Aoki, K.; Sakurai, Y.; Kano, M.; Matsui, H.; Kumada, H. Safety, Pharmacokinetics and Resistant Variants of Telaprevir Alone for 12 Weeks in Hepatitis C Virus Genotype 1b Infection. J Viral Hepat 2012, 19.
413. Namour, F.; Wessels, D.H.; Pascual, M.H.; Reynolds, D.; Sultan, E.; Lenfant, B. Pharmacokinetics of the New Ketolide Telithromycin (HMR 3647) Administered in Ascending Single and Multiple Doses. Antimicrob Agents Chemother 2001, 45, 170–175.
414. Bhargava, V.; Lenfant, B.; Perret, C.; Pascual, M.H.; Sultan, E.; Montay, G. Lack of Effect of Food on the Bioavailability of a New Ketolide Antibacterial, Telithromycin. Scand J Infect Dis 2002, 34, 823–826.
415. Day, R.; Lam, S.; Paull, P.; Wade, D. Effect of Food and Various Antacids on the Absorption of Tenoxicam. British Journal of Clinical Pharmacology 1987, 24, 323–328.
416. Hosseini-Yeganeh, M.; McLachlan, A.J. Physiologically Based Pharmacokinetic Model for Terbinafine in Rats and Humans. Antimicrob Agents Chemother 2002, 46, 2219–2228.
417. Jensen, J.C. Clinical Pharmacokinetics of Terbinafine (Lamisil). Clin Exp Dermatol 1989, 14, 110–113.
418. Nedelman J.; Cramer J. A.; Robbins B.; Gibiansky E.; Chang C. T.; Gareffa S.; Cohen A.; Meligen J. The Effect of Food on the Pharmacokinetics of Multiple-Dose Terbinafine in Young and Elderly Healthy Subjects. Biopharm Drug Dispos 1997, 18, 127–138.
419. Dahan, A.; Altman, H. Food-Drug Interaction: Grapefruit Juice Augments Drug Bioavailability - Mechnism, Extent and Relevance. Eur J Clin Nutr 2004, 58, 1–9.
420. Tax L Absolute Bioavailability of Testosterone after Oral Administration of Testosterone-Undecanoate and Testosterone. Eur J Drug Metab Pharmacokinet 1987, 12, 225–226.
421. Bagchus, W.M.; Hust, R.; Maris, F.; Schnabel, P.G.; Houwing, N.S. Important Effect of Food on the Bioavailability of Oral Testosterone Undecanoate. Pharmacotherapy 2003, 23, 319–325.
422. Agwuh, K.N.; MacGowan, A. Pharmacokinetics and Pharmacodynamics of the Tetracyclines Including Glycylcyclines. J Antimicrob Chemother 2006, 58, 256–265.
423. Zgair, A.; Lee, J.B.; Wong, J.C.M.; Taha, D.A.; Aram, J.; di Virgilio, D.; McArthur, J.W.; Cheng, Y.K.; Hennig, I.M.; Barrett, D.A.; et al. Oral Administration of Cannabis with Lipids Leads to High Levels of Cannabinoids in the Intestinal Lymphatic System and Prominent Immunomodulation. Sci Rep 2017, 7.
424. Karim, A. Effects of Food on the Bioavailability of Theophylline from Controlled-Release Products in Adults. J Allergy Clin Immunol 1986, 78, 695–703.
425. Cavett, C.L.; Li, Z.; McKiernan, B.C.; Reinhart, J.M. Pharmacokinetics of a Modified, Compounded Theophylline Product in Dogs. J Vet Pharmacol Ther 2019, 42, 593–601.
426. Desager, J.P. Clinical Pharmacokinetics of Ticlopidine. Clin Pharmacokinet 1994, 26, 347–355.
427. Shah, J.; Fratis, A.; Ellis, D.; Murakami, S.; Teitelbaum, P. Effect of Food and Antacid on Absorption of Orally Administered Ticlopidine Hydrochloride. J Clin Pharmacol 1990, 30, 733–736.
428. Ashraf, M.W.; Peltoniemi, M.A.; Olkkola, K.T.; Neuvonen, P.J.; Saari, T.I. Semimechanistic Population Pharmacokinetic Model to Predict the Drug–Drug Interaction Between S-Ketamine and Ticlopidine in Healthy Human Volunteers. CPT Pharmacometrics Syst Pharmacol 2018, 7, 687–697.
429. Fu, J.Y.; Che, H.L.; Tan, D.M.Y.; Teng, K.T. Bioavailability of Tocotrienols: Evidence in Human Studies. Nutr Metab 2014, 11.
430. Yap, S.P.; Yuen, K.H.; Wong, J.W. Pharmacokinetics and Bioavailability of α-, γ- and δ-Tocotrienols under Different Food Status. J Pharm Pharmacol 2010, 53, 67–71.

431. Mahipal, A.; Klapman, J.; Vignesh, S.; Yang, C.S.; Neuger, A.; Chen, D.T.; Malafa, M.P. Pharmacokinetics and Safety of Vitamin E δ-Tocotrienol after Single and Multiple Doses in Healthy Subjects with Measurement of Vitamin E Metabolites. Cancer Chemother Pharmacol 2016, 78, 157–165.
432. Dowty, M.E.; Lin, J.; Ryder, T.F.; Wang, W.; Walker, G.S.; Vaz, A.; Chan, G.L.; Krishnaswami, S.; Prakash, C. The Pharmacokinetics, Metabolism and Clearance Mechanisms of Tofacitinib, a Janus Kinase Inhibitor, in Humans. Drug Metab Dispos 2014, 42, 759–773.
433. Lamba, M.; Wang, R.; Fletcher, T.; Alvey, C.; Kushner, J.; Stock, T.C. Extended-Release Once-Daily Formulation of Tofacitinib: Evaluation of Pharmacokinetics Compared With Immediate-Release Tofacitinib and Impact of Food. J Clin Pharmacol 2016, 56, 1362–1371.
434. Olsson, B.; Brynne, N.; Johansson, C.; Arnberg, H. Food Increases the Bioavailability of Tolterodine but Not Effective Exposure. J Clin Pharmacol 2001, 41, 298–304.
435. Påhlman I.; Gozzi P. Serum Protein Binding of Tolterodine and Its Major Metabolites in Humans and Several Animal Species. Biopharm Drug Dispos 1999, 20, 91–99.
436. Rosenfeld, W.E. Topiramate: A Review of Preclinical, Pharmacokinetic, and Clinical Data. Clin Ther 1997, 19, 1294–1308.
437. Doose, D.R.; Walker, S.A.; Gisclon, L.G.; Nayak, R.K. Single-Dose Pharmacokinetics and Effect of Food on the Bioavailability of Topiramate, a Novel Antiepileptic Drug. J Clin Pharmacol 1996, 36, 884–891.
438. Nakanishi, H.; Yonezawa, A.; Matsubara, K.; Yano, I. Impact of P-Glycoprotein and Breast Cancer Resistance Protein on the Brain Distribution of Antiepileptic Drugs in Knockout Mouse Models. Eur J Pharmacol 2013, 710, 20–28.
439. Kramer, W.G. Effect of Food on the Pharmacokinetics and Pharmacodynamics of Torsemide. Am J Ther 1995, 2, 499–503.
440. Cox, D.S.; Papadopoulos, K.; Fang, L.; Bauman, J.; Lorusso, P.; Tolcher, A.; Patnaik, A.; Pendry, C.; Orford, K.; Ouellet, D. Evaluation of the Effects of Food on the Single-Dose Pharmacokinetics of Trametinib, a First-in-Class MEK Inhibitor, in Patients with Cancer. J Clin Pharmacol 2013, 53, 946–954.
441. Leonowens, C.; Pendry, C.; Bauman, J.; Young, G.C.; Ho, M.; Henriquez, F.; Fang, L.; Morrison, R.A.; Orford, K.; Ouellet, D. Concomitant Oral and Intravenous Pharmacokinetics of Trametinib, a MEK Inhibitor, in Subjects with Solid Tumours. Br J Clin Pharmacol 2014, 78, 524–532.
442. Kumar, P.; Thudium, E.; Laliberte, K.; Zaccardelli, D.; Nelsen, A. A Comprehensive Review of Treprostinil Pharmacokinetics via Four Routes of Administration. Clin Pharmacokinet 2016, 55, 1495–1505.
443. Lim, A.; Wang-Smith, L.; Kates, J.; Laurent, A.; Kumar, P.; Laliberte, K. The Effect of Different Meal Compositions on the Oral Bioavailability of Treprostinil Diolamine in Healthy Volunteers. J Clin Pharm Ther 2013, 38, 450–455.
444. Loi, C.M.; Young, M.; Randinitis, E.; Vassos, A.; Koup, J.R. Clinical Pharmacokinetics of Troglitazone. Clin Pharmacokinet 1999, 37, 91–104.
445. Young, M.A.; Lettis, S.; Eastmond, R. Improvement in the Gastrointestinal Absorption of Troglitazone When Taken with, or Shortly after, Food. Br J Clin Pharmacol 1998, 45, 31–35.
446. Teng, R.; Dogolo, L.C.; Willavize, S.A.; Friedman, H.L.; Vincent, J. Oral Bioavailability of Trovafloxacin with and without Food in Healthy Volunteers. J Antimicrob Chemother 1997, 39, 87–92.
447. Teng, R.; Liston, T.E.; Harris, S.C. Multiple-Dose Pharmacokinetics and Safety of Trovafloxacin in Healthy Volunteers. J Antimicrob Chemother 1996, 37, 955–963.
448. VanDenBerg C. M.; Blob L. F.; Kemper E. M.; Azzaro A. J. Tyramine Pharmacokinetics and Reduced Bioavailability with Food. J Clin Pharmacol 2003, 43, 604–609.
449. Yamamoto, Y.; Danhof, M.; de Lange, E.C.M. Microdialysis: The Key to Physiologically Based Model Prediction of Human CNS Target Site Concentrations. AAPS J 2017, 19, 891–909.
450. Kim, B.H.; Lim, H.S.; Chung, J.Y.; Kim, J.R.; Lim, K.S.; Sohn, D.R.; Cho, J.Y.; Yu, K.S.; Shin, S.G.; Paick, J.S.; et al. Safety, Tolerability and Pharmacokinetics of Udenafil, a Novel PDE-5 Inhibitor, in Healthy Young Korean Subjects. Br J Clin Pharmacol 2008, 65, 848–854.
451. Kim, T.E.; Kim, B.H.; Kim, J.R.; Lim, K.S.; Hong, J.H.; Kim, K.P.; Kim, H.S.; Shin, S.G.; Jang, I.J.; Yu, K.S. Effect of Food on the Pharmacokinetics of the Oral Phosphodiesterase 5 Inhibitor Udenafil for the Treatment of Erectile Dysfunction. Br J Clin Pharmacol 2009, 68, 43–46.
452. Yamamoto, S.; Inoue, K.; Murata, T.; Kamigaso, S.; Yasujima, T.; Maeda, J.Y.; Yoshida, Y.; Ohta, K.Y.; Yuasa, H. Identification and Functional Characterization of the First Nucleobase Transporter in Mammals: Implication in the Species Difference in the Intestinal Absorption Mechanism of Nucleobases and Their Analogs between Higher Primates and Other Mammals. J Biol Chem 2010, 285, 6522–6531.
453. Gupta, M.; Kovar, A.; Meibohm, B. The Clinical Pharmacokinetics of Phosphodiesterase-5 Inhibitors for Erectile Dysfunction. J Clin Pharmacol 2005, 45, 987–1003.
454. Mano, Y.; Sugiyama, Y.; Ito, K. Use of a Physiologically Based Pharmacokinetic Model for Quantitative Prediction of Drug-Drug Interactions via CYP3A4 and Estimation of the Intestinal Availability of CYP3A4 Substrates. J Pharm Sci 2015, 104, 3183–3193.
455. Heinig, R.; Weimann, B.; Dietrich, H.; Böttcher, M.F. Pharmacokinetics of a New Orodispersible Tablet Formulation of Vardenafil: Results of Three Clinical Trials. Clin Drug Investig 2011, 31, 27–41.

456. Mostafa, N.M.; Chiu, Y.L.; Rosen, L.S.; Bessudo, A.; Kovacs, X.; Giranda, V.L. A Phase 1 Study to Evaluate Effect of Food on Veliparib Pharmacokinetics and Relative Bioavailability in Subjects with Solid Tumors. Cancer Chemother Pharmacol 2014, 74, 583–591.
457. Nuthalapati, S.; Munasinghe, W.; Giranda, V.; Xiong, H. Clinical Pharmacokinetics and Mass Balance of Veliparib in Combination with Temozolomide in Subjects with Nonhematologic Malignancies. Clin Pharmacokinet 2018, 57, 51–58.
458. Li, J.; Kim, S.; Sha, X.; Wiegand, R.; Wu, J.; Lorusso, P. Complex Disease-, Gene-, and Drug-Drug Interactions: Impacts of Renal Function, CYP2D6 Phenotype, and OCT2 Activity on Veliparib Pharmacokinetics. Clin Cancer Res 2014, 20, 3931–3944.
459. Li, X.; Delzer, J.; Voorman, R.; de Morais, S.M.; Lao, Y. Disposition and Drug-Drug Interaction Potential of Veliparib (ABT-888), a Novel and Potent Inhibitor of Poly(ADP-Ribose) Polymerase. Drug Metab Dispos 2011, 39, 1161–1169.
460. Goldinger, S.M.; Rinderknecht, J.; Dummer, R.; Kuhn, F.P.; Yang, K.H.; Lee, L.; Ayala, R.C.; Racha, J.; Geng, W.; Moore, D.; et al. A Single-Dose Mass Balance and Metabolite-Profiling Study of Vemurafenib in Patients with Metastatic Melanoma. Pharmacol Res Perspect 2015, 3.
461. Ribas, A.; Zhang, W.; Chang, I.; Shirai, K.; Ernstoff, M.S.; Daud, A.; Cowey, C.L.; Daniels, G.; Seja, E.; O'Laco, E.; et al. The Effects of a High-Fat Meal on Single-Dose Vemurafenib Pharmacokinetics. J Clin Pharmacol 2014, 54, 368–374.
462. Salem, A.H.; Agarwal, S.K.; Dunbar, M.; Nuthalapati, S.; Chien, D.; Freise, K.J.; Wong, S.L. Effect of Low- and High-Fat Meals on the Pharmacokinetics of Venetoclax, a Selective First-in-Class BCL-2 Inhibitor. J Clin Pharmacol 2016, 56, 1355–1361.
463. Troy, S.M.; Parker, V.P.; Hicks, D.R.; Pollack, G.M.; Chiang, S.T. Pharmacokinetics and Effect of Food on the Bioavailability of Orally Administered Venlafaxine. J Clin Pharmacol 1997, 37, 954–961.
464. Shen, Z.; Gillen, M.; Miner, J.N.; Bucci, G.; Wilson, D.M.; Hall, J.W. Pharmacokinetics, Pharmacodynamics, and Tolerability of Verinurad, a Selective Uric Acid Reabsorption Inhibitor, in Healthy Adult Male Subjects. Drug Des Devel Ther 2017, 11, 2077–2086.
465. Smith, W.B.; Hall, J.; Berg, J.K.; Kazimir, M.; Yamamoto, A.; Walker, S.; Lee, C.A.; Shen, Z.; Wilson, D.M.; Zhou, D.; et al. Effect of Renal Impairment on the Pharmacokinetics and Pharmacodynamics of Verinurad, a Selective Uric Acid Reabsorption Inhibitor. Clin Drug Investig 2018, 38, 703–713.
466. Lee, C.A.; Yang, C.; Shah, V.; Shen, Z.; Wilson, D.M.; Ostertag, T.M.; Girardet, J.L.; Hall, J.; Gillen, M. Metabolism and Disposition of Verinurad, a Uric Acid Reabsorption Inhibitor, in Humans. Drug Metab Dispos 2018, 46, 532–541.
467. Rowinsky, E.K.; Lucas, V.S.; Hsieh, A.L.Y.; Wargin, W.A.; Hohneker, J.A.; Lubejko, B.; Sartorius, S.E.; Donehower, R.C. The Effects of Food and Divided Dosing on the Bioavailability of Oral Vinorelbine. Cancer Chemother Pharmacol 1996, 39, 9–16.
468. Gauvin, A.; Pinguet, F.; Culine, S.; Astre, C.; Cupissol, D.; Bressolle, F. Blood and Plasma Pharmacokinetics of Vinorelbine in Elderly Patients with Advanced Metastatic Cancer. Cancer Chemother Pharmacol 2002, 49, 48–56.
469. Purkins, L.; Wood, N.; Kleinermans, D.; Greenhalgh, K.; Nichols, D. Effect of Food on the Pharmacokinetics of Multiple-Dose Oral Voriconazole. Br J Clin Pharmacol 2003, 56, 17–23.
470. Li, M.X.; Zhu, L.Q.; Chen, L.; Li, N.; Qi, F. Assessment of Drug-Drug Interactions between Voriconazole and Glucocorticoids. J Chemother 2018, 30, 296–303.
471. Theuretzbacher, U.; Ihle, F.; Derendorf, H. Pharmacokinetic/Pharmacodynamic Profile of Voriconazole. Clin Pharmacokinet 2006, 45, 649–663.
472. Dekhuijzen, P.N.R.; Koopmans, P.P. Pharmacokinetic Profile of Zafirlukast. Clin Pharmacokinet 2002, 41, 105–114.
473. Madsen, C.M.; Boyd, B.; Rades, T.; Müllertz, A. Supersaturation of Zafirlukast in Fasted and Fed State Intestinal Media with and without Precipitation Inhibitors. Eur J Pharm Sci 2016, 91, 31–39.
474. Devineni, D.; Gallo, J.M. Zalcitabine: Clinical Pharmacokinetics and Efficacy. Clin Pharmacokinet 1995, 28, 351–360.
475. Taylor, S.; Pereira, A.S. Antiretroviral Drug Concentrations in Semen of HIV-1 Infected Men. Sex Transm Infect 2001, 77, 4–11.
476. Nazareno, L.A.; Holazo, A.A.; Limjuco, R.; Passe, S.; Twardy, S.K.; Min, B.; Massarella, J.W. The Effect of Food on Pharmacokinetics of Zalcitabine in HIV-Positive Patients. Pharm Res 1995, 12, 1462–1465.
477. Awni, W.M.; Braeckman, R.A.; Granneman, G.R.; Witt, G.; Dubé, L.M. Pharmacokinetics and Pharmacodynamics of Zileuton after Oral Administration of Single and Multiple Dose Regimens of Zileuton 600mg in Healthy Volunteers. Clin Pharmacokinet 1995, 29, 22–33.
478. Awni, W.M.; Cavanaugh, J.H.; Witt, G.; Granneman, G.R.; Dubé, L.M. The Effect of Food on the Pharmacokinetics of Zileuton. Clin Pharmacokinet 1995, 29, 62–66.
479. Brown, D.; Scott, D.H.T.; Scott, D.B.; Meyer, M.; Westerlund, D.; Lundström, J. Pharmacokinetics of Zimelidine - Systemic Availability of Zimelidine and Norzimelidine in Human Volunteers. Eur J Clin Pharmacol 1980, 17, 111–116.
480. Preskorn S. H. The Effect of Food on the Absorption of Oral Ziprasidone. Clin Pharmacokinet 2005, 44, 1117–1133.
481. Preskorn, S.H. Pharmacokinetics and Therapeutics of Acute Intramuscular Ziprasidone. Clin Pharmacokinet 2005, 44, 1117–1133.
482. Dixon, R.; Warrander, A. The Clinical Pharmacokinetics of Zolmitriptan. Cephalalgia 1997, 17, 15–20.

483. Seaber, E.J.; Peck, R.W.; Smith, D.A.; Allanson, J.; Hefting, N.R.; van Lier, J.J.; Sollie, F.A.E.; Wemer, J.; Jonkman, J.H.G. The Absolute Bioavailability and Effect of Food on the Pharmacokinetics of Zolmitriptan in Healthy Volunteers. Br J Clin Pharmacol 1998, 46, 433–439.
484. Paraiso, R.L.M.; Watanabe, A.; Andreas, C.J.; Turner, D.; Zane, P.; Dressman, J. In-Vitro-in-Silico Investigation of the Negative Food Effect of Zolpidem When Administered as Immediate-Release Tablets. J Pharm Pharmacol 2019, 71, 1663–1676.
485. Salvà, P.; Costa, J. Clinical Pharmacokinetics and Pharmacodynamics of Zolpidem: Therapeutic Implications. Clin Pharmacokinet 1995, 29, 142–153.
486. Greenblatt, D.J.; Harmatz, J.S.; Singh, N.N.; Roth, T.; Harris, S.C.; Kapil, R.P. Influence of Food on Pharmacokinetics of Zolpidem from Fast Dissolving Sublingual Zolpidem Tartrate Tablets. J Clin Pharmacol 2013, 53, 1194–1198.
487. Jerling, M.; Dahl, M.L.; Åberg-Wistedt, A.; Liljenberg, B.; Landell, N.E.; Bertilsson, L.; Sjöqvist, F. The CYP2D6 Genotype Predicts the Oral Clearance of the Neuroleptic Agents Perphenazine and Zuclopenthixol. Clin Pharmacol Ther 1996, 59, 423–428.
488. Aaes-Jørgensen T.; Liedholm H.; Melander A. Influence of Food Intake on the Bioavailability of Zuclopenthixol. Drug Nutr Interact 1987, 5, 157–160.
489. Boulton, D.W.; DeVane, C.L.; Liston, H.L.; Markowitz, J.S. In Vitro P-Glycoprotein Affinity for Atypical and Conventional Antipsychotics. Life Sci 2002, 71, 163–169.
490. Du, P.; Li, P.; Liu, H.; Zhao, R.; Zhao, Z.; Yu, W.; Zhou, X.; Liu, L. Open-Label, Randomized, Single-Dose, 2-Period, 2-Sequence Crossover, Comparative Pharmacokinetic Study to Evaluate Bioequivalence of 2 Oral Formulations of Olanzapine Under Fasting and Fed Conditions. Clin Pharmacol Drug Dev 2020, 9, 621–628.
491. Tóth, K.; Sirok, D.; Kiss, Á.; Mayer, A.; Pátfalusi, M.; Hirka, G.; Monostory, K. Utility of in Vitro Clearance in Primary Hepatocyte Model for Prediction of in Vivo Hepatic Clearance of Psychopharmacons. Microchemical J 2018, 136, 193–199.
492. Hu, C.; Chen, X.; Zhao, Z.; Gao, D.; Gong, S.; Zhang, L.; Xu, Y.; Li, L.; Zhang, L. A Two-Sequence, Four-Period, Crossover, Replicate Study to Demonstrate Bioequivalence of Warfarin Sodium Tablet in Healthy Chinese Subjects Under Fasting and Fed Conditions. Clin Pharmacol Drug Dev 2020, 9, 527–536.
493. Zaman, R.; Wilkins, M.R.; Kendall, M.J.; Jack, D.B. The Effect of Food and Alcohol on the Pharmacokinetics of Acebutolol and Its Metabolite, Diacetolol. Biopharm Drug Dispos 1984, 5, 91–95.
494. Ochoa, D.; Román, M.; Cabaleiro, T.; Saiz-Rodríguez, M.; Mejía, G.; Abad-Santos, F. Effect of Food on the Pharmacokinetics of Omeprazole, Pantoprazole and Rabeprazole. BMC Pharmacol Toxicol 2020, 21.
495. Shibata, M.; Toyoshima, J.; Kaneko, Y.; Oda, K.; Kiyota, T.; Kambayashi, A.; Nishimura, T. The Bioequivalence of Two Peficitinib Formulations, and the Effect of Food on the Pharmacokinetics of Peficitinib: Two-Way Crossover Studies of a Single Dose of 150 Mg Peficitinib in Healthy Volunteers. Clin Pharmacol Drug Dev 2021, 10, 283–290.
496. Yamamoto, S.; Kosugi, Y.; Hirabayashi, H.; Moriwaki, T. Impact of P-Glycoprotein on Intestinal Absorption of an Inhibitor of Apoptosis Protein Antagonist in Rats: Mechanisms of Nonlinear Pharmacokinetics and Food Effects. Pharm Res 2018, 35, 1–8.
